# The Importance of Stereochemistry in 5-HT_7_R Modulation—A Case Study of Hydantoin Derivatives

**DOI:** 10.1021/acschemneuro.4c00152

**Published:** 2024-10-21

**Authors:** Katarzyna Kucwaj-Brysz, Sebastian Baś, Ewa Żesławska, Sabina Podlewska, Magdalena Jastrzębska-Więsek, Anna Partyka, Wojciech Nitek, Grzegorz Satała, Anna Wesołowska, Jadwiga Handzlik

**Affiliations:** † Department of Technology and Biotechnology of Drugs, Jagiellonian University Medical College, Medyczna 9, 30-688 Kraków, Poland; ‡ Faculty of Chemistry, Jagiellonian University, Gronostajowa 2, 30-387 Kraków, Poland; § Institute of Biology and Earth Sciences, University of the National Education Commission, Krakow, Podchorążych 2, 30-084 Kraków, Poland; ∥ Maj Institute of Pharmacology, Polish Academy of Sciences, Smętna 12, 31-343 Kraków, Poland; ⊥ Department of Clinical Pharmacy, Jagiellonian University, Medical College, Medyczna 9, 30-688 Kraków, Poland

**Keywords:** 5-HT_7_ receptor, stereochemistry, depression, anxiety, hydantoin, piperazine

## Abstract

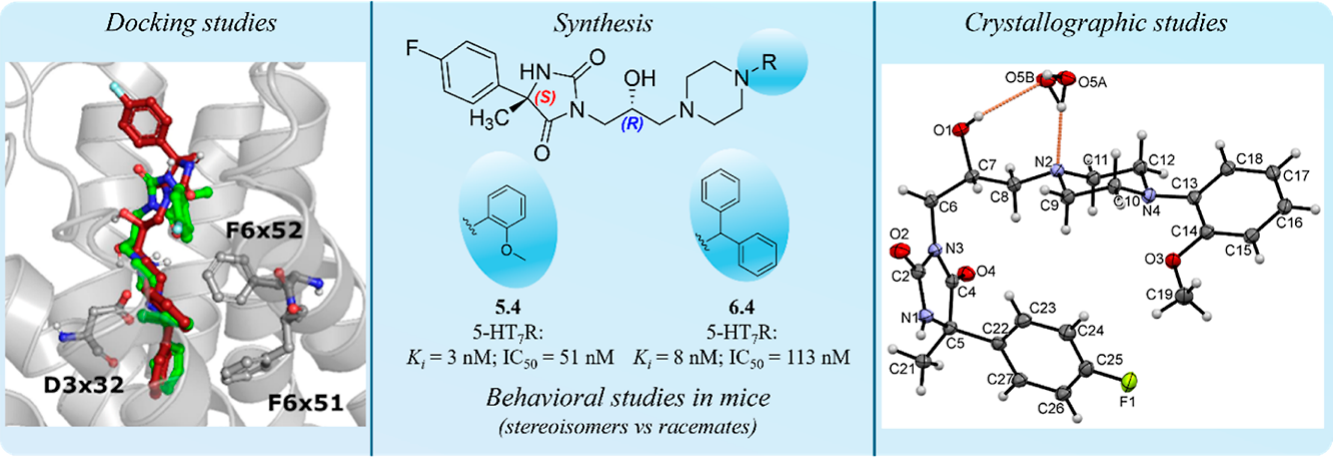

Serotonin 5-HT_7_ receptor (5-HT_7_R), one of the most recently discovered members of the serotonergic system, has become a promising target in the search for central nervous system disorders. Despite the number of preclinical results, none of the selective 5-HT_7_R agents has been approved; therefore, the clinical significance of this protein has not been confirmed yet. Recently, we described very promising, selective, and highly potent hydantoin-derived 5-HT_7_R antagonists with confirmed antidepressant activity *in vivo* and a very good ADMET profile; however, they have been tested in behavioral studies as racemates. In this work, the synthesis of optically pure hydantoin-derived 5-HT_7_R agents using cost-effective, classical methods has been presented for the first time. X-ray crystallographic analysis confirmed the absolute configuration on both stereogenic centers and allowed for the elucidation of the mechanism of introduction of epichlorohydrin into the hydantoin N3-position. The radioligand binding results showed a clear configuration preference for 5-HT_7_R affinity. The molecular modeling results further indicated the key interaction responsible for lower affinity (with amino acid I3 × 29). Finally, the comparison of the antidepressant and anxiolytic effects of racemates versus stereoisomers suggests an influence of additional, apart from the action on 5HT_7_R, factors responsible for the activity *in vivo*, which is worthy of deeper insight within further studies.

## 1 Introduction

The serotonin 5-HT_7_ receptor (5-HT_7_R) is one of the most recently discovered members of the serotonin (5-HT) family, which has been under deep investigation for decades as a potential therapeutic target for central nervous system dysfunctions. Many preclinical studies with the use of selective 5-HT_7_R ligands have indicated its role in such processes as memory, cognition, thermoregulation, cardiac rhythm, and emotional processes.^1−5^ Moreover, it has been postulated that the antidepressant (mood stabilizing) effect of well-known antipsychotics is a result of antagonism for 5-HT_7_R. However, these compounds do not act selectively, and they bind even more strongly to other 5-HT receptors. Among already approved drugs only lurasidone, a second-generation antipsychotic with antidepressant and anxiolytic activity, shows relatively high 5-HT_7_R affinity.^6^ The evaluation of clinical efficacy suggests the influence of 5-HT_7_R blocking on overall mood stabilization of lurasidone.^7−9^ Additionally, very recent studies have shown that 5-HT_7_R may also be considered as a new target in the search for prostate cancer,^10^ breast cancer,^11^ and neuroblastoma therapy.^12^ Finally, the latest study with compound LP-211 indicated 5-HT_7_R as a potential therapeutic target in searching for neuroinflammation modulators.^13^

It is worth noting that highly selective 5-HT_7_R ligands are still missing from the pharmaceutical market. Hence, for advanced clinical trials to confirm the real pharmacological role of this receptor, searching for novel chemical agents with such pharmacodynamic profiles and beneficial ADMETox properties is absolutely needed.

The biomolecules that are considered therapeutic targets (enzymes, receptors) can recognize stereoisomers as different chemical entities, thus leading to differences in binding affinity, and consequently, different biological effects.^14−16^ Stereoselectivity has been observed in all the pharmacokinetic processes, i.e., absorption, distribution, metabolism, and excretion (ADME properties).^17,18^ Toxicity may also be drastically different for enantiomers, e.g., (*S*)-naproxen is used for arthritis pain, whereas (*R*)-naproxen causes liver poisoning.^19^ Similarly, thalidomide,^20^ a drug for the treatment of morning sickness in pregnant women, was withdrawn as racemate due to the teratogenicity of the (*S*)-enantiomer.^21^ In summary, the isolation of a proper stereoisomer may lead to increased desired pharmacological activity, a beneficial pharmacokinetic profile, and elimination of undesired toxic effects. However, the enantioselective synthesis or chiral resolution of racemates is very often highly challenging or expensive, which complicates an evaluation of the biological properties of the individual stereoisomers.

For the time being, stereochemical considerations among 5-HT_7_R ligands resulted in optically pure 5-HT_7_R ligands that do not differentiate in terms of 5-HT_7_R affinity^22^ and those that differentiate significantly,^23^ thereby suggesting that 5-HT_7_R enantioselectivity may depend on a particular class of chemical compounds. Although the *in vivo* studies on optically pure 5-HT_7_R agents are limited, very interesting results have been reported for enantiomers of amisulpride, the second-generation antipsychotic, where the *R*-enantiomer targeted 5-HT_7_R while the *S*-enantiomer acted on D_2_R. Hence, that study highlighted the high probability of a 5-HT_7_R-mediated antidepressant effect of racemic amisulpride.^24^

For the past few years, our group has concentrated on the search for selective very strong 5-HT_7_R antagonists among hydantoin-derived phenylpiperazines.^25−28^ The two most promising compounds found (**1** and **2**, Figure 2) showed a high 5-HT_7_R affinity (with *K*_*i*_ < 100 nM), a very good ADMETox profile *in vitro*, and antidepressant-like effects in mice.^25,27−29^ Worthy of note is that the previously discovered “hit” compounds contain two stereogenic centers located on carbon atoms (Figure 2), but initially, they were tested as a mixture of two pairs of diastereoisomers.

**Figure 1:**
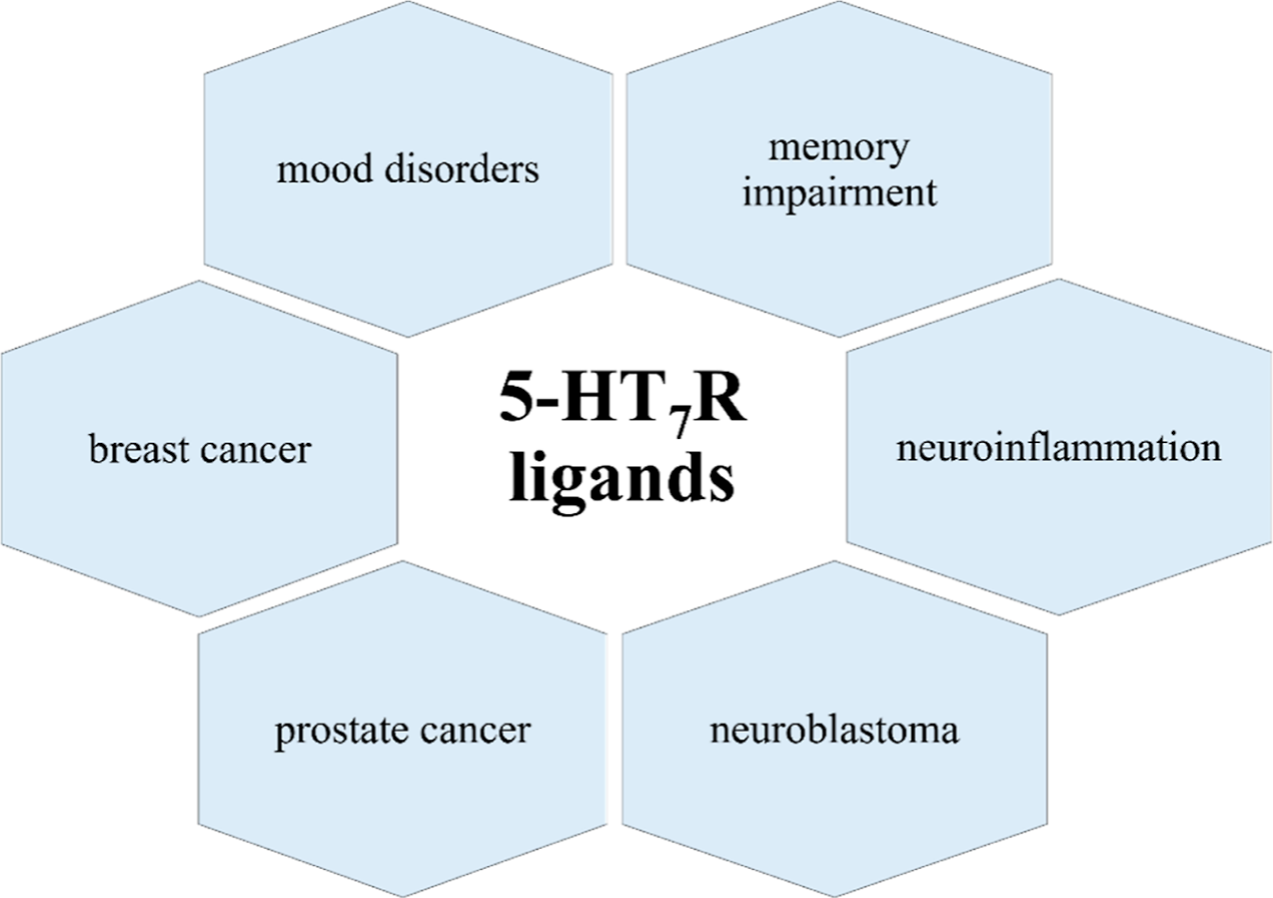
Possible applications of the 5-HT_7_R ligands.

**Figure 2:**
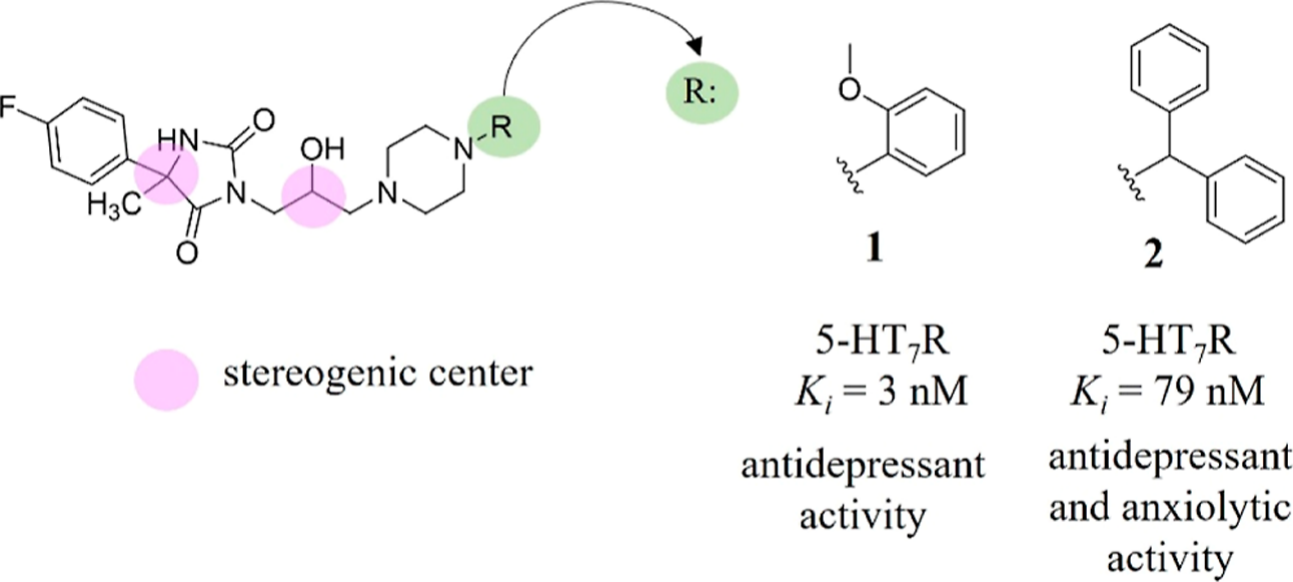
Chemical structure of reference “hit” compounds **1** and **2** with marked stereogenic centers.

Recently, we succeeded in the separation of the diastereoisomers for compound **1** with the use of the superfluid chromatography technique.^30^ We observed with great interest significant differences in 5-HT_7_R affinities and ADMET properties depending on the absolute configurations. However, those absolute configurations were only predicted by theoretical calculations (using DFT—density functional theory). Moreover, separation with the aforementioned chromatographical resolution technique by superfluid chromatography was expensive and not general enough to enable the procedure either for compound **2** or for larger amounts of both compounds in order to perform *in vivo* studies.

Thus, the optically pure stereoisomers of compounds **1** and **2**, by the use of a simple, cost-effective, and importantly, enabling definition of the stereochemistry, synthetic method, have been synthesized for the first time within the present study. The compounds were investigated *in vitro* for 5-HT_7_R, 5-HT_1A_R, and 5-HT_2A_, D_2_R affinities, then, *in vivo* for potential antidepressant- and anxiolytic-like action in mice. Additionally, X-ray crystallography was performed to finally confirm the absolute configurations, while molecular modeling studies were used to support the elucidation of the occurring qualitative structure–activity relationship (SAR) analysis.

## 2 Results and Discussion

### 2.1 Chemical Synthesis

**Scheme 1:**
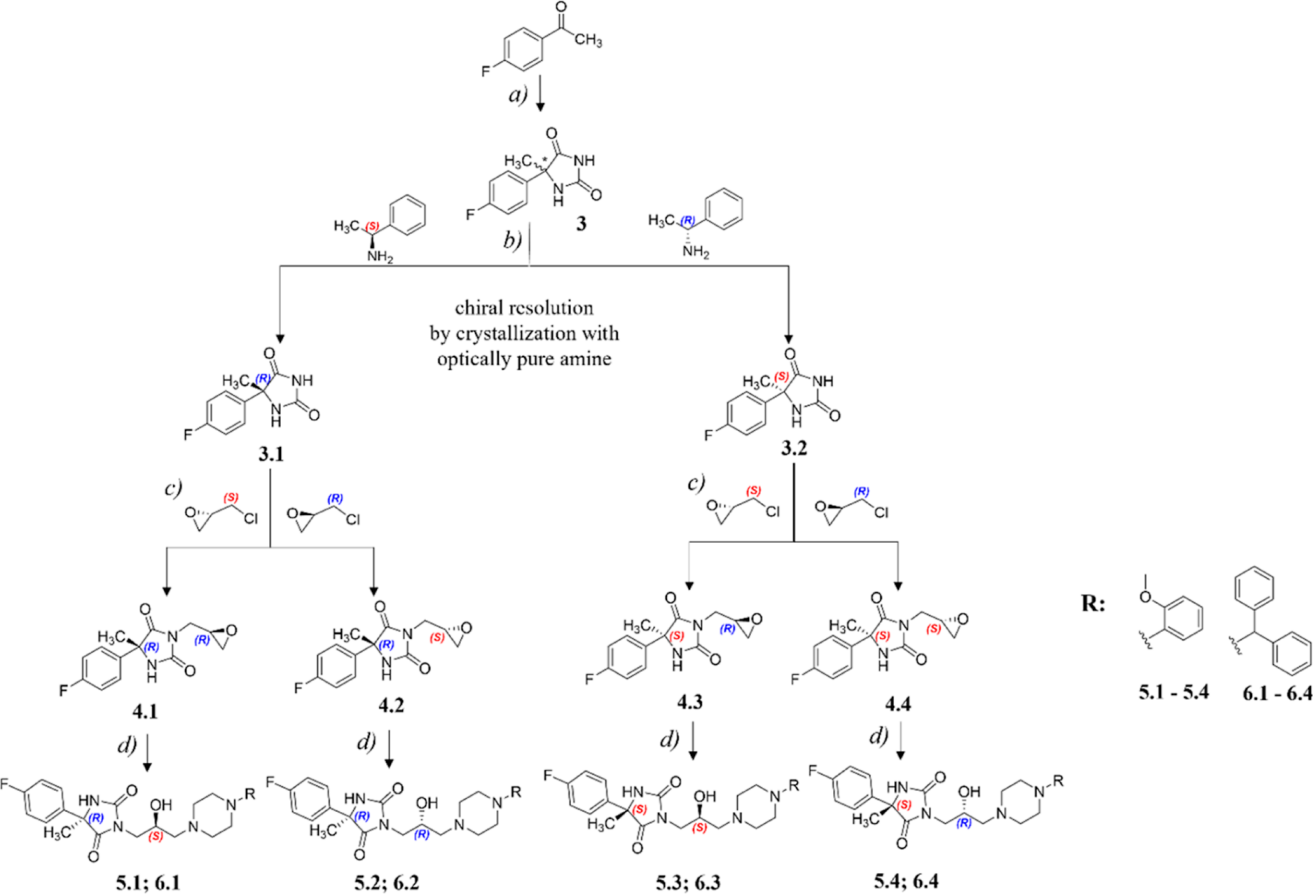
Synthetic Route to Stereoisomers **5.1–5.4** and **6.1–6.4**: (*a*) KCN, NH_4_(CO_3_)_2_, H_2_O/Ethanol, 55 °C, (b) 1. NaOH, H_2_O; 2. 1 M HCl, (*c*) NaOH, H_2_O, rt, and (*d*) Isopropanol, Reflux

In the first step of the synthesis pathway, the hydantoin ring formation according to the previously described procedure for the Bucherer–Bergs reaction^31^ was performed. As the hydantoin formation is nonstereospecific, the chiral resolution of enantiomers was achieved via classic crystallization of the diastereomeric salts of hydantoin enantiomers with *S*- and *R*-phenethylamine (Scheme 1). Subsequently, the optically pure enantiomers of the aryl hydantoin, compounds **3.1** and **3.2**, were used for *N*-alkylation with *S*- and *R*-epichlorohydrin–which resulted in oxirane derivatives **4.1–4.4**. In the final step, an epoxide ring-opening reaction with appropriate piperazine derivatives to give the final stereoisomers **5.1–5.4** and **6.1–6.4** was carried out.

### 2.2 X-ray Studies

#### 2.2.1 Crystallographic Studies on Intermediates **3.1** and **3.2**

**Figure 3:**
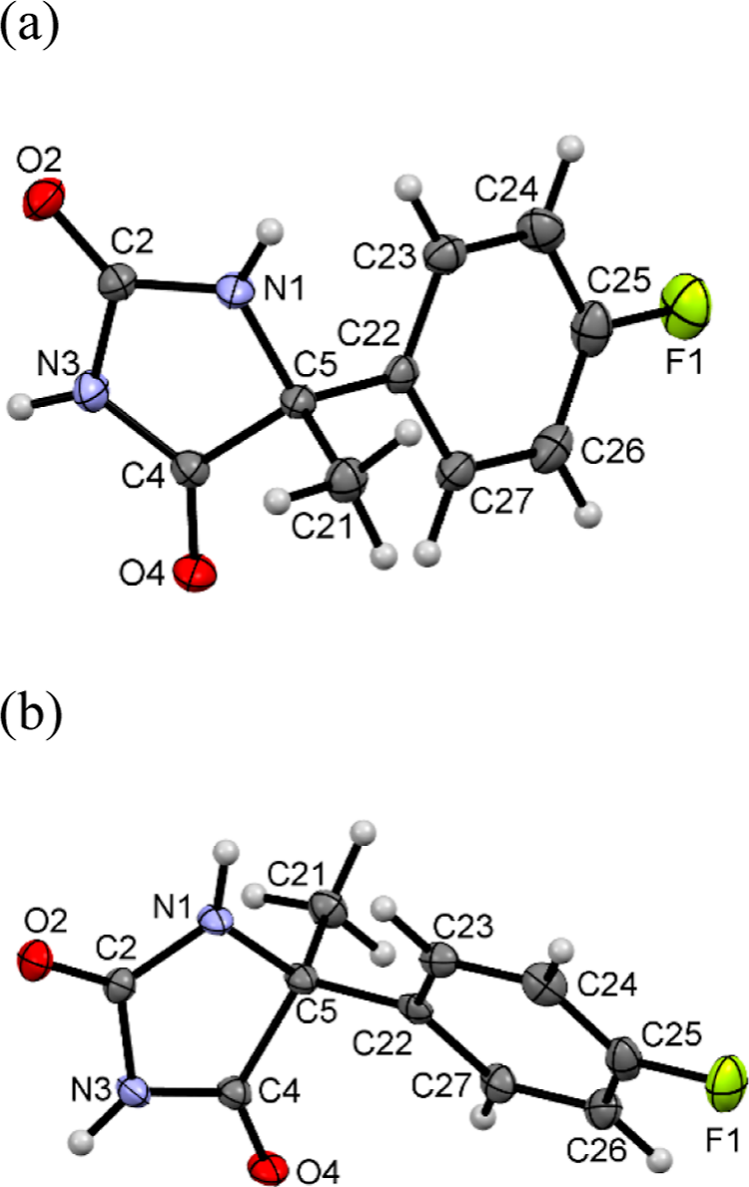
Asymmetric unit of (a) **3.1** and (b) **3.2**, with the atom numbering schemes. Displacement ellipsoids are drawn at the 50% probability level.

In order to confirm the efficacy of chiral resolution via crystallization (Scheme 1, step *b*) for the obtained crystals of intermediates **3.1** and **3.2**, crystallographic studies were conducted. Both compounds crystallized in the orthorhombic space group *P*2_1_2_1_2_1_ with one molecule in the asymmetric unit (Figure 3). The crystal structure of the polymorph of **3.1**, in the space group *Pbca*, was determined earlier.^32^

**Figure 4:**
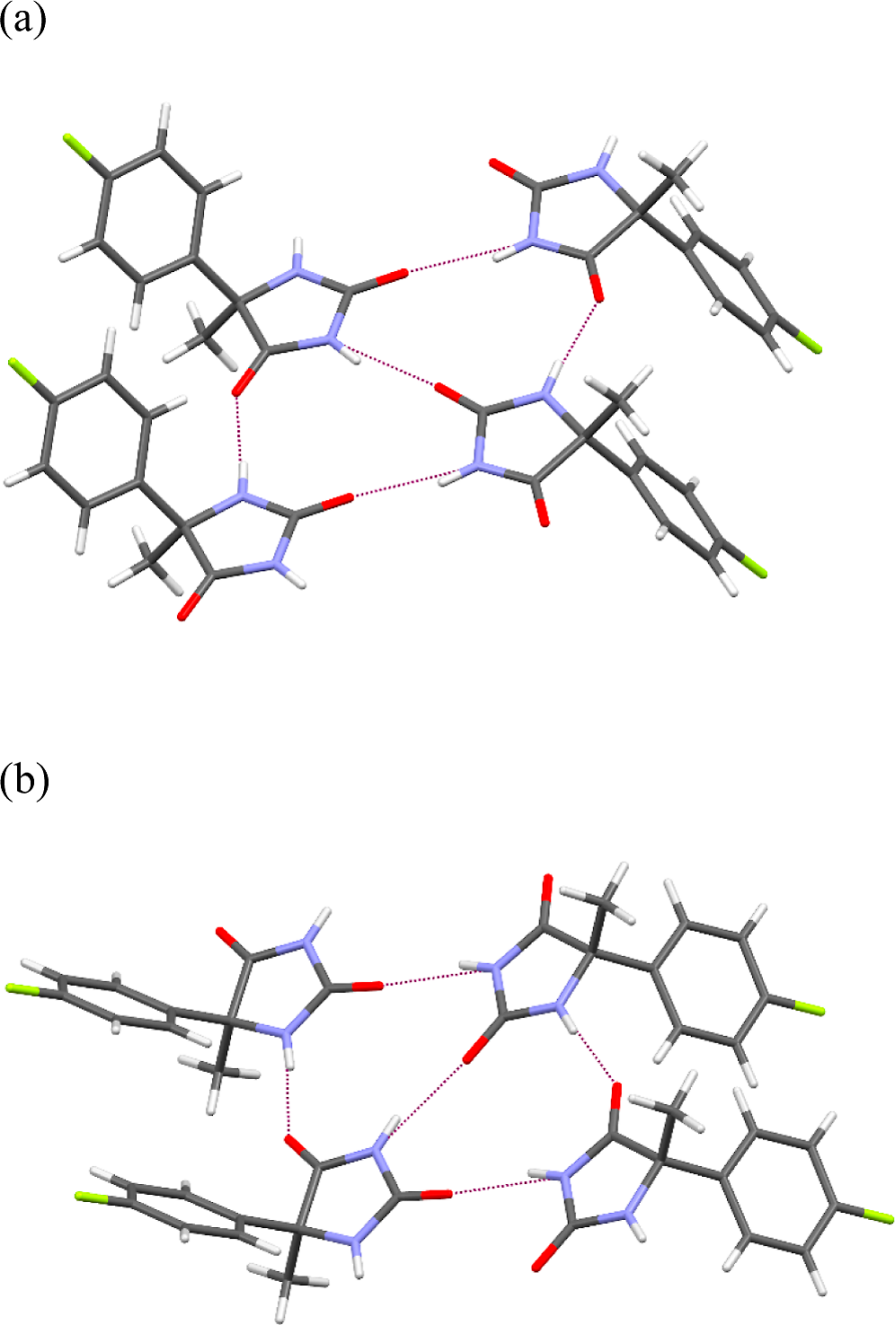
Interactions of four molecules of (a) **3.1** and (b) **3.2** in the crystals. Purple dashed lines indicate hydrogen bonds.

The assignment of the absolute configuration of the enantiomers, *R* for **3.1** and *S* for **3.2**, was confirmed. The angles between the hydantoin and aromatic rings have values of 60.52(6) and 60.42(6)° for **3.1** and **3.2**, respectively. Molecular packing is mainly determined by the intermolecular hydrogen bonds involving oxygen (O2, O4) and nitrogen (N1, N3) atoms. For both crystal structures, the same type of hydrogen bond can be observed (Figure 4).

#### 2.2.2 Crystal and Molecular Structure of Stereoisomers **5.1**–**5.4**

**Figure 5:**
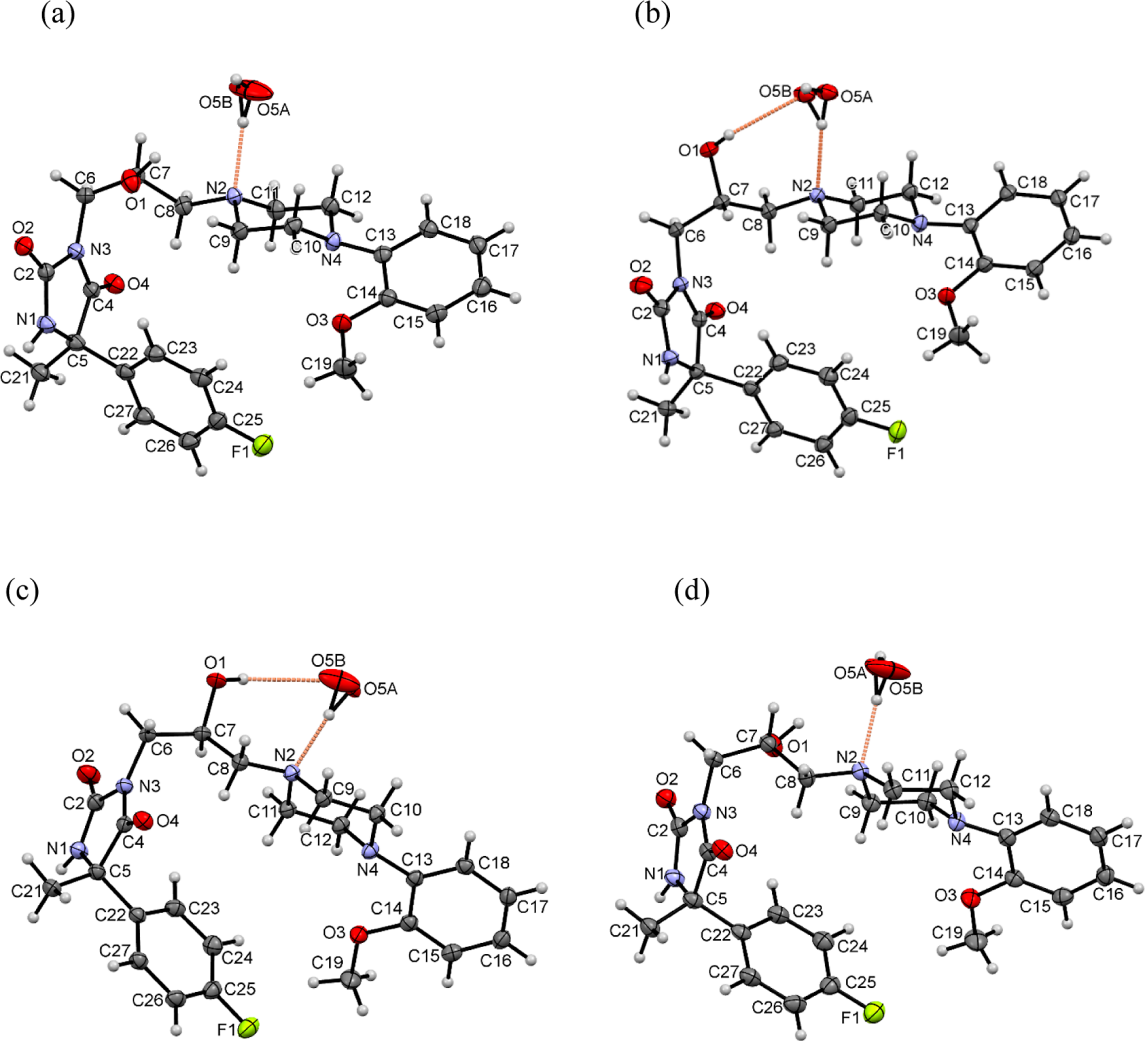
Molecular geometries of (a) **5.1**, (b) **5.2**, (c) **5.3**, and (d) **5.4** showing the atom numbering schemes. The orange dashed lines indicate hydrogen bonds. Displacement ellipsoids are drawn at the 50% probability level.

All four stereoisomers crystallized in orthorhombic space group *P*2_1_2_1_2_1_ with one molecule of the investigated compound and one disordered water molecule in the asymmetric unit. The molecular geometries of the investigated stereoisomers with the atom numbering schemes are shown in Figure 5.

Each of the molecules possesses two chiral centers on the C5 and C7 atoms. The crystal structures confirmed the (5*R*, 7*S*) configuration for **5.1**, (5*R*, 7*R*) for **5.2**, (5*S*, 7*S*) for **5.3**, and (5*S*, 7*R*) for **5.4**. The linker between the hydantoin and the piperazine rings adopts a bent conformation in all four compounds with the torsion angles N3–C6–C7–C8 being 60.0(2), 73.8(2), −73.8(2), and −60.2(3)° and C6–C7–C8–N2 being −171.9(2), −168.7(2), 168.7(1), and 171.8(2)° for **5.1**, **5.2**, **5.3**, and **5.4**, respectively. The conformation of the linker is different in comparison to the geometry observed in the crystal structure determined earlier for hydrochloride with the protonated N2 atom.^33^ The hydroxy group at the C7 atom of the linker is engaged in intramolecular interactions with one carbon atom of the piperazine ring, similar to other crystal structures containing the same linker between the hydantoin and the piperazine rings.^26,27,30^ The molecular structures are stabilized by an intramolecular C–H···O hydrogen bond between the oxygen atom of the methoxy group and the carbon atom of the piperazine ring. This type of interaction is observed in other crystal structures containing the *N*-(*o*-methoxyphenyl)piperazine moiety, which are deposited in the Cambridge Structural Database (CSD).^34^ The piperazine ring adopts a chair conformation with an equatorial location of the substituents at the N2 and N4 atoms. The N2 atom is involved as an acceptor in the hydrogen bond with the water molecule. The angles between the planes of the aromatic and piperazine (C9, C10, C11, and C12) rings are 41.74(9), 41.5(1), 41.60(8), and 41.8(1)° for **5.1**, **5.2**, **5.3**, and **5.4**, respectively. Similar values of angles have been observed in other crystal structures of hydantoin derivatives containing the *o*-methoxyphenyl substituent at the nitrogen atom of the piperazine rings.^33,35^ The angles between the planes of the aromatic ring at the C5 atom and the hydantoin ring are 826(2), 84.73(7), 84.81(6), and 826(7)° for **5.1**, **5.2**, **5.3**, and **5.4**, respectively. The values of these angles are greater than those in other derivatives containing the 5-(4-fluorophenyl)hydantoin moiety, for which the crystal structures were determined earlier.^30,36^

**Figure 6:**
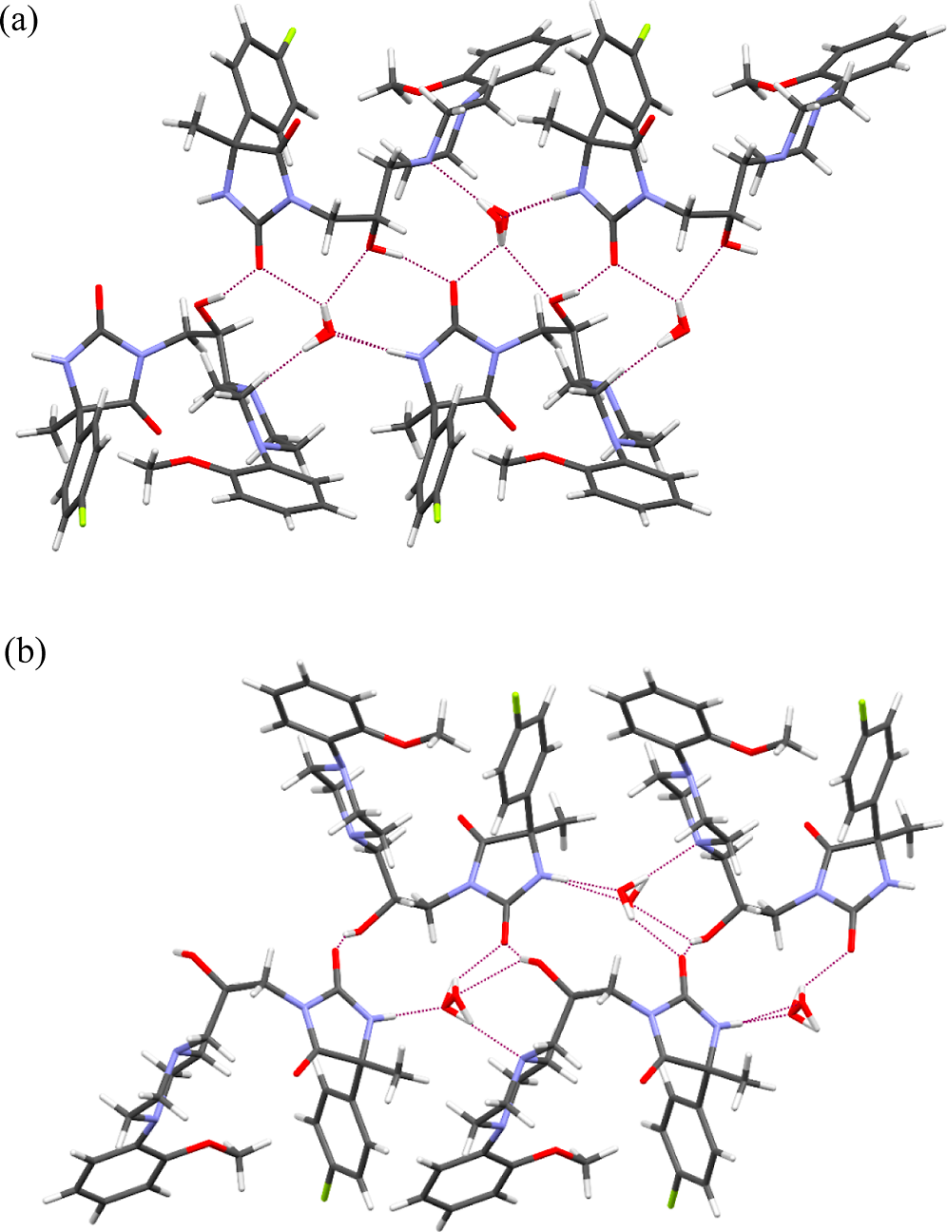
Interactions of four molecules of (a) **5.1** and (b) **5.2**. Purple dashed lines indicate hydrogen bonds.

The packings of molecules in the crystals are determined by N–H···O, O–H···O, and O–H···N intermolecular interactions. Each water molecule interacts with three molecules of the investigated compounds. Furthermore, the hydroxy group is involved in hydrogen bonding with the oxygen atom of the hydantoin ring. In the case of **5.2** and **5.3**, the hydroxy group makes hydrogen bonds as a donor not only with the hydantoin ring but also with the water molecule. These interactions are presented in Figure 6 for selected two-crystal structures of **5.1** and **5.2**. Additionally, the crystal structures are stabilized by C–H···O and C–H···F contacts.

### 2.3 Stereochemical vs Mechanistic Studies

The configuration on C5 of hydantoin depends on the particular enantiomer of phenethylamine used for chiral resolution, whereas the configuration on the carbon atom C7 (in the 2-hydroxypropyl linker) depends on the particular enantiomer of epichlorohydrin used for the second step.

**Figure 7:**
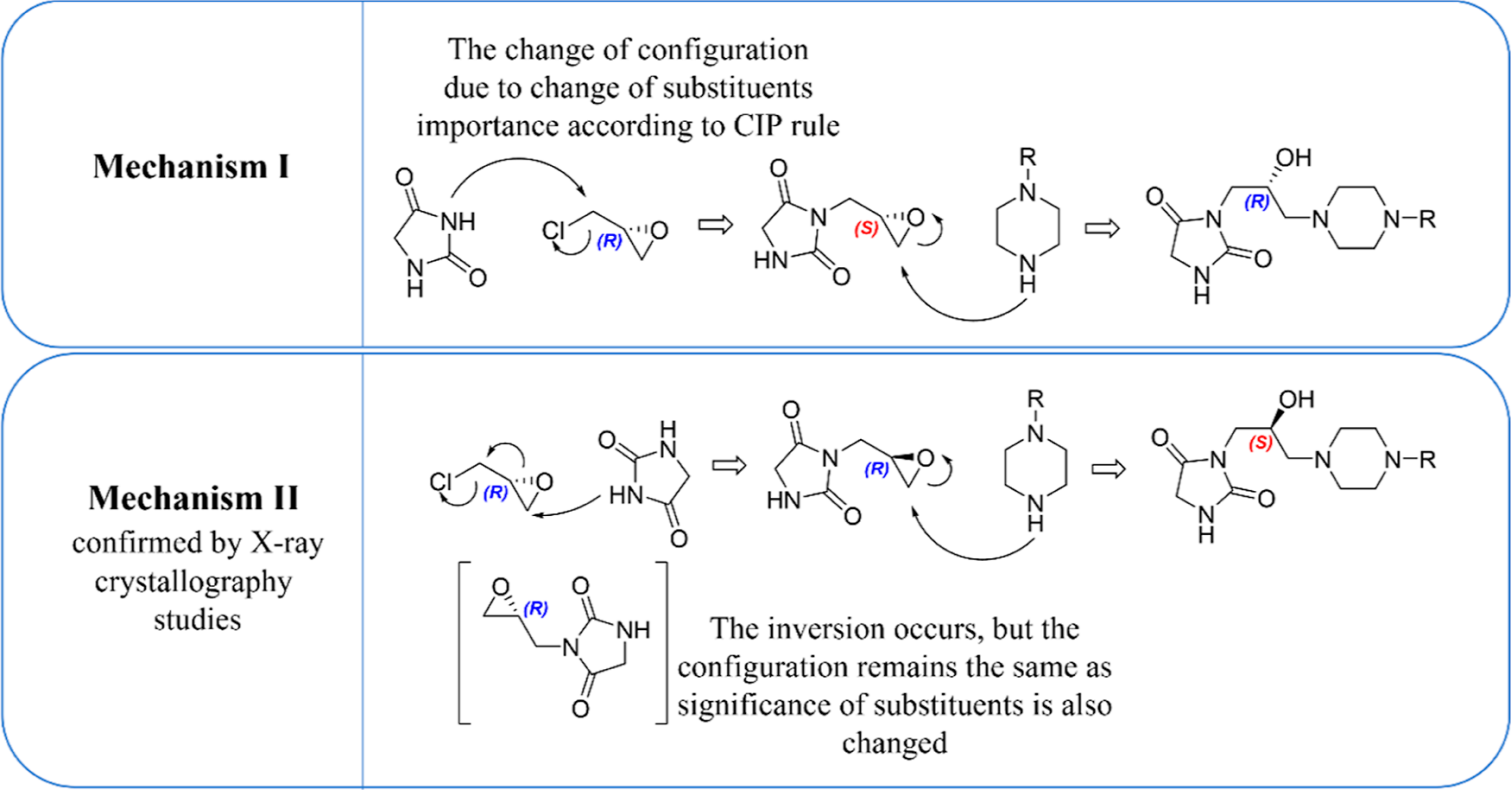
Two probable mechanisms for the reaction of hydantoin with epichlorohydrin. Mechanism II was confirmed by X-ray crystallography studies for the final compounds.

However, two mechanisms of attachment of epichlorohydrin to hydantoin can be considered in this particular case. In Mechanism I, the hydantoin anion formed after treatment of acidic imide with the strong base (NaOH) substitutes the chloride in epichlorohydrin (Figure 7). In this way, the configuration of the stereogenic center of epichlorohydrin remains unchanged. However, according to the generally approved Cahn–Ingold–Prelog (CIP) rules for the description of configuration, the discussed unchanged stereogenic center should now be labeled *S*.^37,38^ The subsequent nucleophilic addition of piperazine to the chiral epoxide, which is a stereospecific reaction, results in the formation of a single enantiomer product with an inverted configuration due to the nucleophilic addition from the other side, and the alternative and more probable mechanism in applied conditions is based on the nucleophilic attack of the hydantoin anion on the epoxide ring, leading to the alkoxide intermediate formation.^39−41^ Further stereospecific, intramolecular substitution of chloride by the alkoxy group results in the regeneration of the epoxide ring with inverted stereochemistry. The same issue with the nomenclature of the CIP rules is also applicable here. Even though the proper configuration underwent the inversion after the reaction, the CIP priority rules impose the classification of the current stereogenic center in the configuration as *R*, the same as for the starting material.

The crystallographic studies confirmed the determinacy of Mechanism II; however, the HPLC analysis revealed the presence of a second enantiomer at a low level (∼3%). Hence, Mechanism I also occurs but at a significant minority.

Importantly, in the last step, i.e., the condensation with piperazine derivatives, the configuration is changed as a result of changing the substituent priority according to the CIP rule (Figure 7).

### 2.4 *In Vitro* Studies

#### 2.4.1 Radioligand Binding Assays

The final compounds **5.1**–**5.4** and **6.1**–**6.4** were tested in the radioligand binding assay for their affinity toward 5-HT_7_R and its common off-targets. The results obtained, compared with those for respective racemates (**1** and **2**) in Table 1, indicate the highest 5-HT_7_R affinity, accompanied by significant selectivity over receptors D_2_, 5-HT_1A_, and 5-HT_2A_, for compounds **5.4** and **6.4.**, showing very interesting regularities which will be discussed in the SAR analysis section.

**Table 1:** Affinities for Compounds **5.1**–**5.4** and **6.1**–**6.4** in Comparison to Racemates

compound	stereochemistry	*K*_*i*_ [nM]^a^			
		D_2_	5-HT_1A_	5-HT_2A_	5-HT_7_
**1**	*racemate*	715 ± 163	121 ± 18	nt^b^	**3 ± 2**
**5.1**	*5R, 7S*	462 ± 94	939 ± 153	50,740 ± 11 567	**1787±389**
**5.2**	*5R, 7R*	712 ± 101	1884 ± 351	41,140 ± 5694	**5215±807**
**5.3**	*5S, 7S*	1109 ± 137	219 ± 43	95,160 ± 23 921	**74±11**
**5.4**	*5S, 7R*	888 ± 97	306 ± 71	21,840 ± 4396	**5±2**
**2**	*racemate*	261 ± 38	5570 ± 1412	nt	**79±6**
**6.1**	*5R, 7S*	463 ± 75	15 800 ± 3491	12,320 ± 1577	**2901±461**
**6.2**	*5R, 7R*	1521 ± 331	40 240 ± 9517	11,390 ± 2435	**9091±1697**
**6.3**	*5S, 7S*	312 ± 52	10 740 ± 2667	4130 ± 983	**120±27**
**6.4**	*5S, 7R*	511 ± 96	8930 ± 1836	5786 ± 1068	**8±3**

a Data are presented as the mean ± SD from two independent measurements.

b nt—not tested.

#### 2.4.2 Evaluation of Intrinsic Activity

Our previous studies on racemic mixtures showed that all the compounds from the hydantoin-derived series show antagonistic activity.^29^ However, as a particular absolute configuration may also have influenced intrinsic activities, functional tests were performed for the stereoisomers with the highest 5-HT_7_R affinity (**5.4** and **6.4**) to evaluate their ability to inhibit cAMP production. The results confirm that the investigated stereoisomers are also 5-HT_7_R antagonists (Table 2).

**Table 2:** Results from cAMP Functional Assays toward 5-HT_7_R^a^

compound	IC_50_ ± SEM [nM]	*K*_*b*_ ± SEM [nM]
methiothepin	2.9 ± 0.5	0.35 ± 0.09
**5.4**	51 ± 12	6.1 ± 1.2
**6.4**	113 ± 25	14 ± 3

a Data are presented as the mean ± SD from two independent measurements.

### 2.5 *In Vivo* Experiments

**Figure 8:**
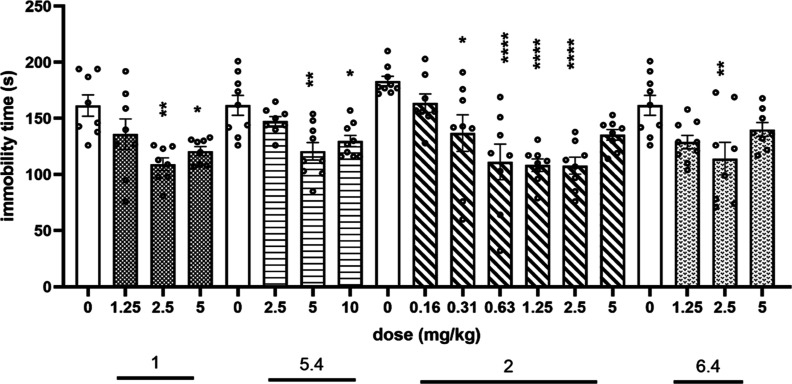
Antidepressant-like activity of compounds **1**, **5.4**, **2**, and **6.4** in FST in mice. The compounds were injected i.p. 60 min before the test. Values represent the mean ± SEM during the last 4 min test session compared to the respective vehicle group (one-way ANOVA is followed by Bonferroni’s post hoc test). *N* = 7–9. *p < 0.05, **p < 0.01, ****p < 0.0001.

Compounds **5.4** and **6.4** were selected for behavioral assays in mouse models. The aim was to compare the pharmacological effects of the most potent stereoisomers with those of racemic mixtures **1** and **2** (also tested in this study). We used the forced swim test (FST) (Porsolt’s test) to evaluate the antidepressant-like activity of the compounds. The obtained results indicate that compound **1** given at doses of 2.5 and 5 mg/kg produced antidepressant-like effects in testing (*F*(3,28) = 6.2675; *p* < 0.01), significantly reducing by about 32% and 25%, respectively, the immobility time of animals compared to the vehicle group (Figure 8). The compound **5.4** (5*S*,7*R* enantiomer of **1**) showed lower activity than compound **1**, producing antidepressant-like activity at doses of 5 and 10 mg/kg and decreasing immobility by 25% and 20%, respectively (*F*3,31) = 7.1077; *p* < 0.001) (Figure 8). A similar mode of action was observed for compound **2** and its 5*S*,7*R* enantiomer **6.4**. Compound **2**, administered at a dose of 0.312 mg/kg, significantly decreased the immobility of mice by 25% and its activity was maintained at doses of 0.63, 1.25, and 2.5 mg/kg (immobility decreasing significantly by about 40% after administration at these doses) (*F*(6,50) = 9.0255; *p* < 0.0001). The highest used dose of **2** (5 mg/kg) decreased immobility by 26%, producing, however, a nonsignificant effect (Figure 8). For the 5*S*,7*R* enantiomer **6.4**, the significant antidepressant-like activity was observed only at a dose of 2.5 mg/kg [decreasing immobility by 29%, *F*(3,30) = 4.6459; *p* < 0.01] (Figure 8). The obtained antidepressant-like effects for the investigated compounds (**1**, **2**, **5.4**, and **6.4**) were comparable with those for imipramine and SB269970 (a selective 5-HT_7_R antagonist), respectively, each injected i.p. at a dose of 10 mg/kg in our previous studies.^25^

**Figure 9:**
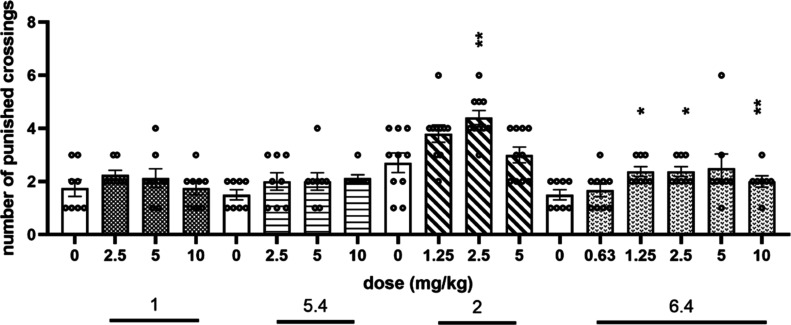
Anxiolytic-like activity of compounds **1**, **5.4**, **2**, and **6.4** in FPT in mice. The compounds were injected i.p. 60 min before the test. Values represent the mean ± SEM during 1 min test session compared to the respective vehicle group (one-way ANOVA is followed by Bonferroni’s post hoc test). *N* = 7–8. *p < 0.05, **p < 0.01.

The anxiolytic-like activity was assessed in the four-plate test (FPT) in mice. In this test, compounds **1** and **5.4** were inactive, i.e., *F*(3,28) = 0.8561; NS and *F*(3,28) = 1.1569; and NS, respectively (Figure 9). In contrast, compounds **2** and **6.4** exerted a significant anxiolytic-like effect, increasing the number of punished crossing by about 60% vs respective vehicle groups (*F*(3,36) = 5.9418; *p* < 0.01 and *F*(5,42) = 4.9578; *p* < 0.01, respectively). The enantiomer 5*S*,7*R* (compound **6.4**) was active at doses of 1.25, 2.5, and 10 mg/kg, while the effect of a dose of 5 mg/kg did not reach significant levels due to the large statistical spread of a single result as seen in Figure 9. The racemic mixture (**2**) given only at a dose of 2.5 mg/kg presented similar anxiolytic-like properties, increasing the number of punished crossings by 62% (Figure 9). The obtained anxiolytic-like activity for both compound **2** and its enantiomer (**6.4**) was comparable with that for diazepam (given i.p. at the dose range of 1.25–5 mg/kg) but lower than that for SB269970 given i.p. at a dose of 1 mg/kg, investigated by us previously.^25^

Importantly, the observed antidepressant- and/or anxiolytic-like effects produced by the tested compounds seem to be specific, since their minimal effective doses did not increase the spontaneous locomotor activity of mice during the initial 3–6 min of the session (i.e., at the time identical to that of the observation period in FST), and during the first minute of the session (i.e., at the time identical to that of the observation period in FPT). A significant decrease in locomotor activity was observed for compound **1** (2.5 mg/kg) between 3 and 6 min of the observation time (Table 3).

**Table 3:** Spontaneous Locomotor Activity of Investigated Compounds in Mice

treatment	dose (mg/kg)	locomotor activity number of crossings ± SEM during		
		1 min	3–6 min	60 min
vehicle	0	22.43 ± 8.74	136.43 ± 21.40	1678.43 ± 151.82
1	2.5	10.50 ± 4.11; NS	42.50 ± 14.87; *p* < 0.05	183.75 ± 33.41; *p* < 0.001
		*F*(1,9) = 0.955; NS	*F*(1,9) = 9.236; *p* < 0.05	*F*(1,9) = 47.708; *p* < 0.001
vehicle	0	22.43 ± 8.74	136.43 ± 21.40	1678.43 ± 151.82
5.4	5	18.50 ± 8.00; NS	138.75 ± 36.61; NS	716.25 ± 117.75; *p* < 0.001
		*F*(1,9) = 0.089; NS	*F*(1,9) = 0.003; NS	*F*(1,9) = 17.303; *p* < 0.001
vehicle	0	22.43 ± 8.74	136.43 ± 21.40	1678.43 ± 151.82
2	0.312	9.75 ± 5.45; NS	108.00 ± 42.04; NS	702.75 ± 122.53; NS
	2.5	16.50 ± 6.12; NS	211.50 ± 29.38; NS	2482.75 ± 587.23; NS
		*F*(2,12) = 0.6187; NS	*F*(2,12) = 2.7705; NS	*F*(2,12) = 77.1007; *p* < 0.01
vehicle	0	22.43 ± 8.74	136.43 ± 21.40	1678.43 ± 151.82
6.4	1.25	4.25 ± 0.46; NS	63.75 ± 25.93; NS	694.25 ± 78.90; NS
	2.5	13,25 ± 5.68; NS	105.75 ± 49.57; NS	922.50 ± 74.78; *p* < 0.001
		*F*(2,12) = 1.4064; NS	*F*(2,12) = 1.4261; NS	*F*(2,12) = 14.637; *p* < 0.001

During the 60 min observation period, a decrease in spontaneous locomotor activity was observed, which should be considered within the pharmacological characteristics of the investigated compounds but is not significant for the activity detected in FST and FPT (Table 3). Clinically used antidepressants and anxiolytics such as imipramine or diazepam also produce some reduction of spontaneous locomotor activity in rodents,^42^ and the sedative properties of these drugs are recommended in specific clinical states of psychiatric illness.

The compounds were injected i.p. 60 min before the test. Values represent the mean ± SEM during a 1 min or 3–6 or 60 min test session compared to the respective vehicle group (one-way ANOVA is followed by Bonferroni’s post hoc test). *N* = 5–6, NS-not significant.

### 2.6 Molecular Modeling

#### 2.6.1 Docking Studies

**Figure 10:**
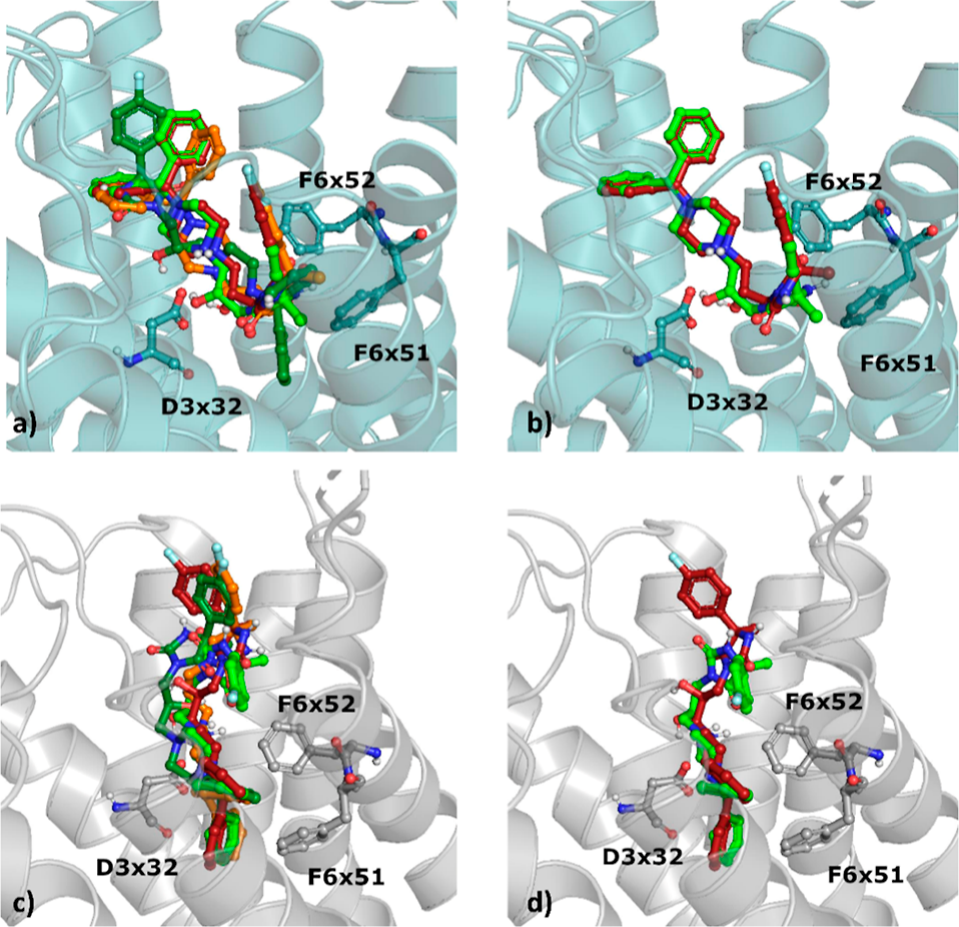
Docking results of stereoisomers of **2** to 5-HT_7_R, (a,b) inactive-state homology model from GPCRdb and (c,d) crystal structure (7XTC); **6.1**—orange, **6.2**—firebrick, **6.3**—dark green, and **6.4**—green.

All the compounds formed a salt bridge between the protonated nitrogen from the piperazine ring and the aspartic acid from the third transmembrane helix (D3 × 32 according to the GPCRdb numbering^43^), whereas a hydrogen bond formed between D3 × 32 and the hydroxyl group (only **6.1** did not form the latter interaction). However, there were slight differences in their orientations in the protein binding pocket (Figure 10).

Interestingly, **6.2**, which displayed very low activity toward 5-HT_7_R (*K*_*i*_ = 9091 ± 1697 nM), adopted the most similar orientation to the most active compound **6.4** (*K*_*i*_ = 8 ± 3 nM) in the 5-HT_7_R homology model. The benzhydrylpiperazine parts of these two compounds were almost perfectly aligned, whereas the fluorophenyl moiety of **6.2** was slightly shifted toward the upper part of the binding pocket in comparison to the analogous part of the molecule of **6.4**. When the compounds were docked to the 5-HT_7_R crystal structure, **6.1**, **6.2**, and **6.3** adopted similar poses, whereas the imidazolidinedione and fluorophenyl parts of **6.4** were bent, which resulted in hydrophobic contact by **6.4** with F6 × 51 and F6 × 52, not observed in the case of **6.2**.

**Figure 11:**
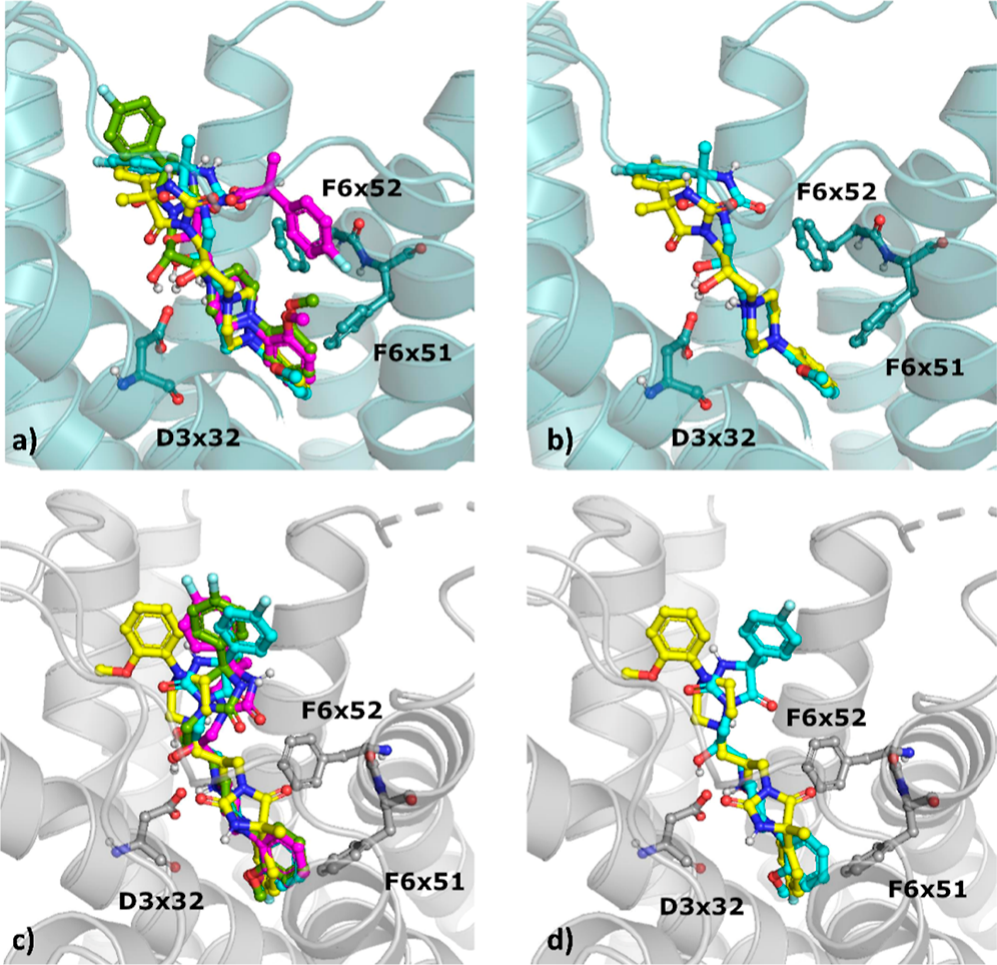
Docking results of stereoisomers of compound **1** to 5-HT_7_R, (a,b) inactive-state homology model from GPCRdb and (c,d) crystal structure (7XTC); **5.1**—magenta, **5.2**—olive, **5.3**—cyan, and **5.4**—yellow.

For comparison, docking was also carried out for the stereoisomers of compound **1** (to both the crystal structure of 5-HT_7_R and the 5-HT_7_R homology model). In general, the docking poses of different stereoisomers of compound **1** were more consistent when docked to the 5-HT_7_R homology model in comparison to those for the crystal structure (Figure 11), i.e., **5.1** had a different conformation of the fluorophenyl part in the 5-HT_7_R homology model, whereas the pose of **5.4** in the 5-HT_7_R crystal structure was flipped in comparison to the orientations of the remaining stereoisomers.

#### 2.6.2 Molecular Dynamics

**Figure 12:**
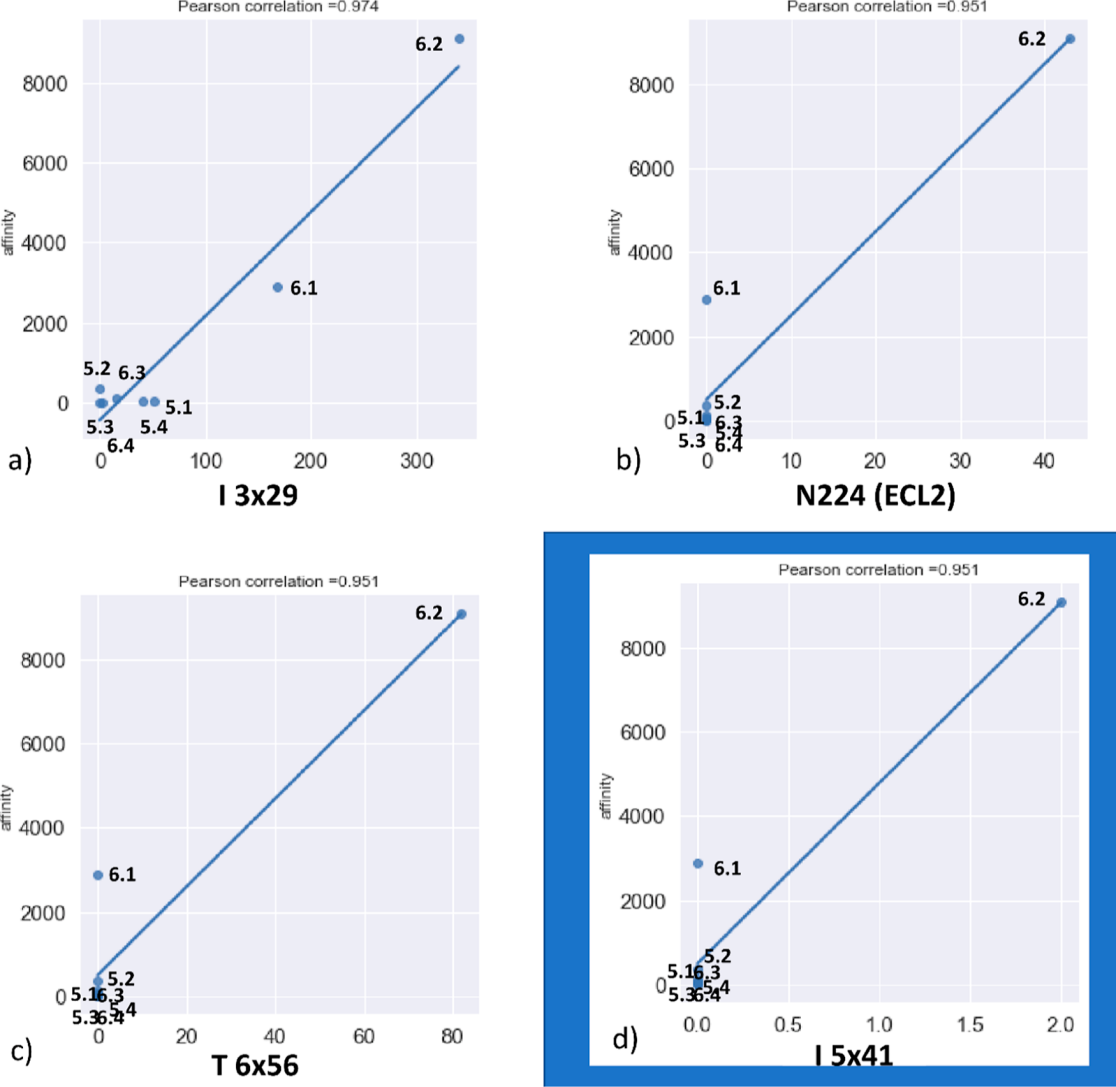
Amino acids with the highest correlation between the frequency of ligand–protein contacts during MD simulation and compound affinity (*K*_*i*_) toward 5-HT_7_R: (a–c)—homology model of 5-HT_7_R and (d) crystal structure of 5-HT_7_R.

**Figure 13:**
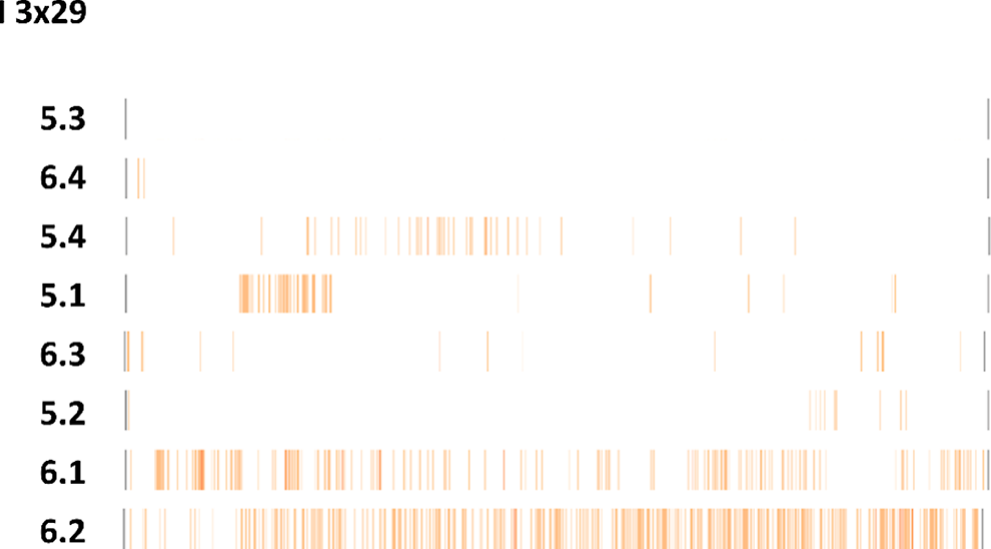
Ligand–protein interactions occurring between ligands and I3 × 29 during MD simulations.

**Figure 14:**
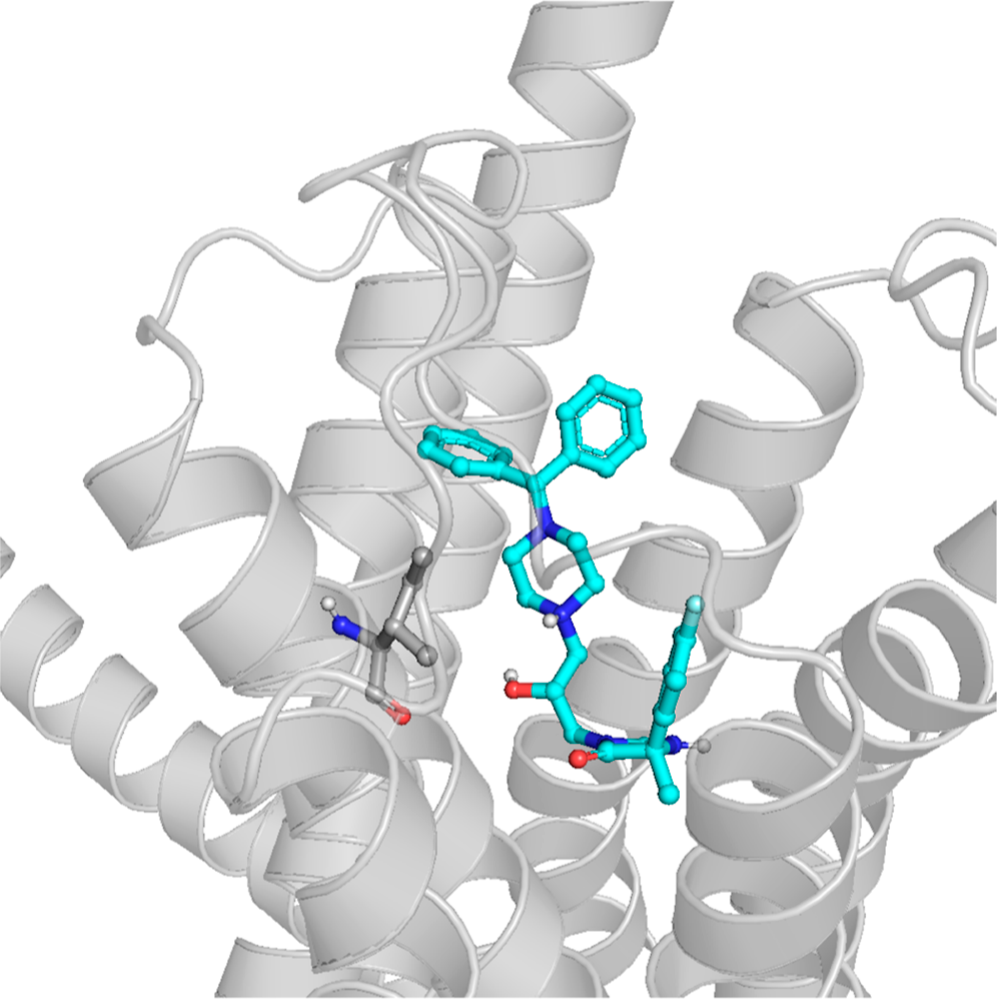
5-HT_7_R homology model with **6.4** ligand docked to its binding site and I3 × 29 visualized.

In order to verify the validity of the obtained docking poses as well as to translate the dynamics of the obtained ligand–protein complexes into possible explanations of the observed activity of particular stereoisomers, MD simulations were carried out. The output of the MD simulations was analyzed from various perspectives. First of all, the stability of docking poses of all examined compounds was verified in the form of a ligand RMSF. No correlation was found between the ligand RMSF and its affinity to the receptor (Figure S1, Supporting Information). After this, the MD output was analyzed in the context of the correlations between the frequency of interactions between ligands and particular amino acids. To fulfill this aim, Pearson’s correlation coefficients between the number of formed contacts during MD simulations and compound *K*_*i*_ were determined. There were several amino acids for which high values of Pearson’s correlation coefficients (above 0.9) were determined. Interestingly, a higher number of highly correlated amino acids was found for the studies with the 5-HT_7_R homology model (3 amino acids) in comparison to that for ligands modeled with the 5-HT_7_R crystal structure (only 1 amino acid)—Figure 12. Three of these positions belong to the transmembrane helices, whereas 1 amino acid is part of the second extracellular loop (N224). Nevertheless, taking into account the relatively low number of contacts occurring during simulation for N224, T6 × 56, and I5 × 41, the correlation obtained for I3 × 29 is of the highest scientific value that can be useful as a reference to design further derivatives of the examined compounds. Ligand–protein interaction diagrams for I3 × 29 are presented in Figure 13, while a visualization of the position of I3 × 29 in the 5-HT_7_R homology model is presented in Figure 14. The correlational studies indicate that for the most active compounds, the contact with I3 × 29 is disfavored, and both **5.4** and **6.4** compounds do not make strong and persistent interactions via this amino acid. Both compounds come into contact with this residue during MD simulations; however, this interaction is quickly lost. The biological activity of these compounds is therefore provided by other contact networks.

### 2.7 SAR Analysis

The radioligand binding results showed very clearly that the level of 5-HT_7_R affinity strongly depends on the absolute configuration of both stereogenic centers of the hydantoin-piperazine derivatives. However, the influence of configuration is more drastically observed in the case of the stereogenic center on carbon atom C5 of hydantoin than on the center of carbon atom C7 bound to the hydroxyl group. For the stereoisomers of both analogues, a similar 5-HT_7_R affinity preference has been maintained, i.e., the configuration *S* for C5 and *R* for C7. Interestingly, racemate **1** showed similar 5-HT_7_R affinity (*K*_*i*_ = 3 ± 2 nM) as stereoisomer **5.4** (*K*_*i*_ = 5 ± 2 nM). The situation differs for compound 2 (*K*_*i*_ = 79 ± 6 nM), which has a significantly lower affinity than stereoisomer **6.4** (*K*_*i*_ = 8 ± 3 nM).

The confrontation of molecular modeling results with the outcome of *in vitro* studies clearly indicates that the increasing contact frequency with I3 × 29 is directly related to a higher *K*_*i*_ value, which means a lower affinity of a compound to 5-HT_7_R. However, no preference for the side of the ligand which makes contact with this amino acid is observed. Thus, for the **6.1–6.4** stereoisomers, it is the phenylpiperazine part that comes into contact with I3 × 29 for the most active ligands, whereas the compounds with lower affinity were oriented toward I3 × 29 with their hydroxy moieties, and these were the cases where the unfavorable contact with I3 × 29 was more frequent. On the other hand, no corresponding analogy for compounds **5.1–5.4** can be observed, as they were all oriented toward I3 × 29 with their hydroxy groups.

Despite the regular dependence between 5-HT_7_R affinity and the configuration of both stereogenic centers, a transfer to *in vivo* animal models was only partially observed. Racemate **1** and its stereoisomer **5.4** showed a similar antidepressant effect, which is consistent with the 5-HT_7_R affinity level (*K*_*i*_ = 3 ± 2 nM for **1** and *K*_*i*_ = 5 ± 2 nM for **5.4**). However, the situation with the pairing of **2** and **6.4** was different as the racemic mixture resulted in stronger antidepressant properties *in vivo*. This effect is inadequate, or even reversed, to the 5-HT_7_R binding results determined for the compounds *in vitro*, i.e., compound **6.4** has an almost 10-fold higher 5-HT_7_R affinity in comparison to **2**. Moreover, the pairing of compounds **1** and **5.4** did not give any anxiolytic effects, whereas pairing of **2** and **6.4** showed significant anxiolytic-like properties. However, in this case, stereoisomer **6.4** has more potent anxiolytic properties than racemic mixture **2**, which may be partially justified by its stronger action on 5-HT_7_R. Nevertheless, the majority of the results obtained indicate that the 5-HT_7_R affinity is not the main factor of the observed antidepressant and anxiolytic effects of compounds **2** and **6.4** in mice. Since the results of the comprehensive studies performed here are not sufficient to clarify this issue, further development of research for the benzhydryl compound’s stereoisomers, covering cellular molecular mechanisms, bioavailability, and a broader panel of depression-related protein targets, is needed in the future.

## 3 Conclusions

In this work, the synthesis of optically pure hydantoin-phenylpiperazine 5-HT_7_R agents, using cost-effective classical methods, was presented for the first time. X-ray crystallographic analysis confirmed not only the absolute configuration on both stereogenic centers but also allowed elucidation of the mechanism of introduction of epichlorohydrin into the N3-position of hydantoin. The radioligand binding assay results showed a clear trend for the 5-HT_7_R-preferable configurations, i.e., the combination of 5*S*/7*R* as the most profitable in terms of the 5-HT_7_R affinity for stereoisomers of both analogues (**1** and **2**). The molecular modeling resulted in the indication of the key interaction (with amino acid I3 × 29) that can be responsible for a decrease in the 5-HT_7_R affinity among the investigated hydantoins. However, the stereoisomers of the methoxyphenyl compound (**5.1**–**5.4**) were differently oriented than the stereoisomers of the benzhydryl one (**6.1**–**6.4**). Finally, the comparison of antidepressant- and anxiolytic-like effects of the racemates versus their stereoisomers suggests that 5-HT_7_R affinity is not the only key factor responsible for the resulting *in vivo* activity, which is worthy of deeper insight within further studies.

## 4 Methodology

### 4.1 Chemical Synthesis

^1^H NMR and ^13^C NMR spectra were recorded at 300 K on an FT-NMR 500 MHz spectrometer JEOL JNM-ECZR500 RS1 (ECZR version) at 500 and 126 MHz, respectively, and on a Bruker AVANCE 300 [^1^H NMR (300 MHz) and ^13^C NMR (75 MHz)]. All of the ^1^H NMR spectra were obtained with use of CD_3_OD and CDCl_3_ as deuterated solvents at ambient temperature using the solvent signal as an internal standard. The values of chemical shifts are expressed as δ values in (ppm) and the coupling constants (*J*) in Hz. Data are reported as follows: chemical shift, multiplicity (s, singlet; br s, broad singlet; d, doublet; t, triplet; dd, doublet of doublet, q, quintet, m, multiplet), coupling constant *J*, number of protons, and protons’ position. TMS or solvent peaks were used as internal standard to determine chemical shifts for ^1^H NMR and solvent peaks were used as internal standard to determine chemical shifts for ^13^C NMR. Hexafluoroacetone trihydrate (CAS: 34202–69–2 from Sigma-Aldrich) was used as internal standard to determine chemical shifts for ^19^F NMR, however, it seems to undergo some side reactions with analyzed compounds. In all cases, the single signal of the reference undergoes split into two to three signals after mixing with analyzed compounds (see the Supporting Information materials for detail). Mass spectra were recorded on a UPLC-MS/MS system consisted of a Waters ACQUITYUPLC (Waters Corporation, Milford, MA, USA), which was coupled to a Waters TQD mass spectrometer (electrospray ionization mode ESI-tandem quadrupole). The UPLC/MS purity of all the final compounds was confirmed to be higher than 95%. Retention time values (*t*_R_) are given in minutes. HRMS spectra were recorded on the UPLC-QT of a system consisting of a Waters Acquity I-Class Plus (Waters Corporation, Milford, MA, USA) coupled to a Waters Synapt XS mass spectrometer (electrospray ionization mode ESI). Chromatographic separations were carried out using the Acquity UPLC BEH (bridged ethylene hybrid) C18 column; 2.1 × 100 mm, and 1.7 μm particle size, equipped with Acquity UPLC BEH C18 VanGuard precolumn; 2.1 × 5 mm, and 1.7 μm particle size. The column was maintained at 40 °C and eluted under gradient conditions using 95 to 0% of eluent A over 10 min, at a flow rate of 0.3 mL min-1. Eluent A: water/formic acid (0.1%, v/v); eluent B: acetonitrile/formic acid (0.1%, v/v).

Chromatograms were recorded using a Waters eλ PDA detector. Spectra were analyzed in the 200–700 nm range with a 1.2 nm resolution and sampling rate of 20 points/s.

MS detection settings of Waters Synapt XS mass spectrometer were as follows: source temperature 150 °C, desolvation temperature 350 °C, desolvation gas flow rate 600 L h-1, cone gas flow 100 L h-1, capillary potential 3.00 kV, and cone potential 30 V. Nitrogen was used for both nebulizing and drying gas. The data were obtained in a resolution scan mode ranging from 50 to 1000 *m*/*z* in time intervals of 0.1 s intervals. Leu-enkephalin was used as a mass reference compound.

#### 4.1.1 Data Acquisition Software was MassLynx V 4.2 (Waters)

Thin-layer chromatography was performed on precoated Merck silica gel 60 F254 aluminum sheets. The reactions at fixed temperature were carried out using a magnetic stirrer with a Heidolph MR 2001 contact thermometer.

Chiral HPLC analysis of final products was conducted on an HPLC system (AZURA HPLC Plus System, Knauer, Berlin, Germany), equipped with Chiralpak AD-H, AS-H, and OZ-H columns (250 × 4.6 mm) and UV–vis detector at detection wavelengths λ = 254, 320, 370, and 440 nm. The detailed conditions applied for the chiral HPLC analysis are provided in the synthetic procedure for each compound.

##### 4.1.1.1 Chiral Resolution of Enantiomers **3.1** and **3.2**

To obtain the starting hydantoin (**3.0**), the Bucherer–Bergs reaction was carried out according to previously reported procedure.^43^ The separation of enantiomers of **3.1** and **3.2** was performed using the modified, reported procedure from Patent US_6344564_B1_p28–29.^44^

##### 4.1.1.2 (*S*)-5-(4-Fluorophenyl)-5-methylimidazolidine-2,4-dione (**3.2**)

Sodium hydroxide (0.3 g, 7.5 mmol, 0.5 equiv) was added to a suspension of hydantoin **3** (2.80 g, 14.7 mmol, 1 equiv) in water (65 mL). The reaction mixture was heated to 40 °C and stirred for 15 min and then (*R*)-phenylethylamine (1.8 mL, 14.5 mmol, 1 equiv) was added. The temperature was increased to 50 °C and maintained for 45 to 60 min with continuous stirring of the solution. During this time, the white solid may start to precipitate. After being cooled to rt, the reaction mixture was left for 3 days. The white precipitate was filtrated, washed with water (3 × 7 mL), and dried in an air stream (approximately 2h). The diastereomeric salt obtained (1.37 g, white crystals) was dissolved in 1 M HCl (10 mL), cooled to <10 °C, and stirred for 2 h. The precipitate was filtered and recrystallized from water/MeOH to produce pure hydantoin as white crystals (0.69 g, 49%) with 60% *ee*. [HPLC analysis: Chiralpak AD-H column (250 × 4.6 mm), hexane/i-PrOH = 85/15 (v/v), flow rate: 1.0 mL/min, temperature 21 °C, detection at λ = 254 nm, (*R*)-enantiomer 8.4 min, and (*S*)-enantiomer 12.6 min]. The mother liquor (after filtration of the diastereomeric salt) enriched with the (*R*)-enantiomer was evaporated under reduced pressure, and the residue was acidified by 1 M HCl. The precipitate (1.92 g) was reused in the procedure of compound **3.2** resolution (see 4.1.1.2.). The mother liquor (after filtration of the diastereomeric salt from the 4.1.1.2 procedure) enriched with the (*S*)-enantiomer was evaporated under reduced pressure, and the residue was acidified with 1 M HCl. The precipitated product was combined with the previous portion (0.69 g, 60% *ee*) to obtain 1.5 g of hydantoin **3** in total. The entire procedure was repeated with (*R*)-phenylethylamine (1.0 mL, 7.8 mmol, 1 equiv) in 35 mL water and NaOH (0.16 g, 4 mmol, 0.5 equiv) to obtain the final product in 0.41 g (55% yield) with >95% *ee*. White solid. Yield 0.41 g, 55%. C10H9FN2O2 (*M*_W_ 208.19); all spectroscopic data have been in full agreement with those reported for compound **3**; ^1^H NMR (600 MHz, CD_3_OD): δ 7.54 (dd, *J* = 9.0, 5.2 Hz, 2H), 7.11 (t, *J* = 8.8 Hz, 2H), 1.75 (s, 3H); ^13^C NMR (151 MHz, CD_3_OD): δ 179.0, 164.8, 163.2, 158.8, 136.9, 128.6, 128.5, 116.4, 116.3, 65.7, 25.6; ^19^F NMR (282 MHz, CD_3_OD) −116.5; [α]_D_^20^ = −112.8 (c 1.01, EtOH); HRMS (ESI) [M + 1] found 209.0714 (calcd 209.0726); Chiral HPLC: Chiralpak AD-H column (250 × 4.6 mm), hexane/i-PrOH = 85/15 (v/v), flow rate: 1.0 mL/min, temperature 21 °C, detection at λ = 254 nm, minor: 8.4 min, main: 12.6min > 95% *ee*.

##### 4.1.1.3 (*R*)-5-(4-Fluorophenyl)-5-methylimidazolidine-2,4-dione (**3.1**)

Sodium hydroxide (0.34 g, 8.5 mmol, 0.5 equiv) was added to a suspension of hydantoin **3** (3.16 g, 16.6 mmol, 1 equiv, including the previous 1.92 g from the procedure 4.1.1.1.) in water (75 mL). The reaction mixture was heated to 40 °C and stirred for 15 min and then (*S*)-phenylethylamine (2.2 mL, 16.4 mmol, 1 equiv) was added. The temperature was increased to 50 °C and maintained for 45 min with continuous stirring of the solution. After being cooled to rt, the reaction mixture was left for 3 days. The white precipitate was filtrated, washed with water (3 × 10 mL), and dried in an air stream. The diastereomeric salt obtained was dissolved in 1 M HCl (12 mL) cooled to <10 °C, and stirred for 2 h. The precipitate was filtered and recrystallized from water/MeOH to produce pure hydantoin as white crystals (1.02 g, 65%) with >99% *ee*.

White solid. Yield 1.02 g,%. C_10_H_9_FN_2_O_2_ (*M*_W_ 208.19); all spectroscopic data have been in full agreement with those reported for compound **3**; ^1^H NMR (600 MHz, CD_3_OD): δ 7.54 (dd, *J* = 9.0, 5.2 Hz, 2H), 7.11 (t, *J* = 8.8 Hz, 2H), 1.75 (s, 3H); ^13^C NMR (151 MHz, CD_3_OD): δ 179.0, 164.8, 163.2, 158.8, 136.9, 128.6, 128.5, 116.4, 116.3, 65.7, 25.6; ^19^F NMR (282 MHz, CD_3_OD): δ −116.5; [α]_D_^20^ = +118.0 (c 1.01, EtOH); HRMS (ESI) [M + 1] found 209.0714 (calcd 209.0726); Chiral HPLC: Chiralpak AD-H column (250 × 4.6 mm), hexane/i-PrOH = 85/15 (v/v), flow rate: 1.0 mL/min, temperature 21 °C, detection at λ = 254 nm, main: 8.4 min, minor: 12.6min > 99% *ee*.

#### 4.1.2 Physicochemical Data for the Final Compounds **5.1–5.4** and **6.1–6.4**

The chiral intermediates **4.1–4.4** were obtained in the reaction of **3.1** and **3.2** with the appropriate chiral epichlorohydrin (Scheme 1). Next, the resulting epoxides reacted with 2-methoxyphenyl piperazine to give series **5.1**–**5.4** and with benzhydrylpiperazine to give series **6.1–6.4**. The above-described process was carried out according to previously reported procedure.^27^

##### 4.1.2.1 (*R*)-5-(4-Fluorophenyl)-3-((*S*)-2-hydroxy-3-(4-(2-methoxyphenyl)piperazin-1-yl)propyl)-5-methylimidazolidine-2,4-dione (**5.1**)

White solid (0.44 g, Yield 64%). C_24_H_29_FN_4_O_4_ (*M*_W_ 456.52) all spectroscopic data have been in full agreement with those reported for compound **1**; ^1^H NMR (600 MHz, CDCl_3_): δ 7.53–7.47 (m, 2H), 7.10–7.04 (m, 2H), 7.03–6.97 (m, 1H), 6.93–6.89 (m, 2H), 6.88–6.82 (m, 1H), 6.48 (s, 1H), 4.04 (ddt, *J* = 9.7, 8.1, 4.1 Hz, 1H), 3.85 (s, 3H), 3.65 (dd, *J* = 13.9, 7.6 Hz, 1H), 3.56 (dd, *J* = 13.9, 4.3 Hz, 1H), 3.05 (br s, 4H), 2.79 (br s, 2H), 2.58 (br s, 2H), 2.47–2.33 (m, 2H), 1.83 (s, 3H); ^13^C NMR (151 MHz, CDCl_3_): δ 175.6, 157.0, 152.3, 141.2, 134.6, 127.4, 127.4, 123.2, 121.1, 118.3, 116.0, 115.8, 111.3, 64.7, 63.4, 61.6, 55.5, 53.6, 50.8, 43.0, 25.6; ^19^F NMR (282 MHz, CDCl_3_): δ −113.5; [α]_D_^20^ = −48.4 (c 1.00, CHCl_3_); HRMS (ESI) [M + 1] found 457.2226 (calcd 457.2251); Chiral HPLC Chiralpak AS-H column (250 × 4.6 mm), hexane/i-PrOH = 80/20 (v/v) + 0.5% Et_3_N, flow rate: 0.8 mL/min, temperature 29 °C, detection at λ = 254 nm, minor:18.7 min, main: 20.8 min, minor: 24.5 min, 86% *de*.

##### 4.1.2.2 (*R*)-5-(4-Fluorophenyl)-3-((*R*)-2-hydroxy-3-(4-(2-methoxyphenyl)piperazin-1-yl)propyl)-5-methylimidazolidine-2,4-dione (**5.2**)

White solid (0.43 g, Yield 63%). C_24_H_29_FN_4_O_4_ (*M*_W_ 456.52) all spectroscopic data have been in full agreement with those reported for compound **1**; ^1^H NMR (600 MHz, CDCl_3_): δ 7.53–7.46 (m, 2H), 7.11–7.05 (m, 2H), 7.03–6.97 (m, 1H), 6.94–6.90 (m, 2H), 6.88–6.83 (m, 1H), 5.90 (s, 1H), 4.01 (tt, *J* = 8.6, 4.4 Hz, 1H), 3.85 (s, 3H), 3.64 (dd, *J* = 13.9, 7.9 Hz, 1H), 3.57 (dd, *J* = 13.9, 4.2 Hz, 1H), 3.06 (br s, 4H), 2.85–2.75 (m, 2H), 2.59 (br s, 2H), 2.47–2.36 (m, 2H), 1.85 (s, 3H); ^13^C NMR (151 MHz, CDCl_3_): δ 175.5, 156.8, 152.4, 141.2, 134.5, 127.4, 127.4, 123.2, 121.1, 118.3, 116.1, 115.9, 111.3, 64.6, 63.3, 61.6, 55.5, 53.6, 50.8, 43.1, 25.7. ^19^F NMR (282 MHz, CDCl_3_): δ −113.4; [α]_D_^20^ = −38.2 (c 1.01, CHCl_3_); HRMS (ESI) [M + 1] found 457.2226 (calcd 457.2251); Chiral HPLC: Chiralpak AS-H column (250 × 4.6 mm), hexane/i-PrOH = 80/20 (v/v) + 0.5% Et_3_N, flow rate: 0.8 mL/min, temperature 29 °C, detection at λ = 254 nm, minor: 21.1 min, main: 24.8 min, 97% *de*.

##### 4.1.2.3 (*S*)-5-(4-Fluorophenyl)-3-((*S*)-2-hydroxy-3-(4-(2-methoxyphenyl)piperazin-1-yl)propyl)-5-methylimidazolidine-2,4-dione (**5.3**)

White solid (0.35 g, Yield 51%); C_24_H_29_FN_4_O_4_ (*M*_W_ 456.52); all spectroscopic data have been in full agreement with those reported for compound **1**; ^1^H NMR (600 MHz, CDCl_3_): δ 7.52–7.46 (m, 2H), 7.11–7.06 (m, 2H), 7.03–6.97 (m, 1H), 6.94–6.89 (m, 2H), 6.88–6.83 (m, 1H), 5.92 (s, 1H), 4.01 (dq, *J* = 12.2, 4.3 Hz, 1H), 3.85 (s, 3H), 3.64 (dd, *J* = 13.9, 7.9 Hz, 1H), 3.57 (dd, *J* = 13.9, 4.2 Hz, 1H), 3.06 (br s, 4H), 2.80 (br s, 2H), 2.59 (br s, 2H), 2.47–2.36 (m, 2H), 1.85 (s, 3H); ^13^C NMR (151 MHz, CDCl_3_): δ 175.4, 156.7, 152.4, 141.2, 127.5, 123.2, 121.1, 118.3, 116.1, 115.9, 111.3, 64.6, 63.3, 61.6, 55.5, 53.6, 50.8, 43.1, 25.6; ^19^F NMR (282 MHz, CDCl_3_): δ −113.4, −113.5; [α]_D_^20^ = 41.5 (c 0.83, CHCl_3_); HRMS (ESI) [M + 1] found 457.2226 (calcd 457.2251); Chiral HPLC: Chiralpak AS-H column (250 × 4.6 mm), hexane/i-PrOH = 80/20 (v/v) + 0.5% Et_3_N, flow rate: 0.8 mL/min, temperature 29 °C, detection at λ = 254 nm, main: 17.8 min, > 99% *de*.

##### 4.1.2.4 (*S*)-5-(4-Fluorophenyl)-3-((*R*)-2-hydroxy-3-(4-(2-methoxyphenyl)piperazin-1-yl)propyl)-5-methylimidazolidine-2,4-dione (**5.4**)

White solid (0.47 g, Yield 69%); C_24_H_29_FN_4_O_4_ (*M*_W_ 456.52); all spectroscopic data have been in full agreement with those reported for compound **1**; ^1^H NMR (600 MHz, CDCl_3_): δ 7.53–7.47 (m, 2H), 7.11–7.04 (m, 2H), 7.02–6.97 (m, 1H), 6.94–6.89 (m, 2H), 6.85 (d, *J* = 8.0 Hz, 1H), 6.25 (s, 1H), 4.04 (ddt, *J* = 9.7, 8.1, 4.1 Hz, 1H), 3.85 (s, 3H), 3.65 (dd, *J* = 13.9, 7.6 Hz, 1H), 3.56 (dd, *J* = 13.9, 4.3 Hz, 1H), 3.05 (br s, 4H), 2.79 (br s, 2H), 2.58 (br s, 2H), 2.48–2.35 (m, 2H), 1.84 (s, 3H); ^13^C NMR (151 MHz, CDCl_3_): δ 175.5, 156.9, 152.4, 141.2, 127.4, 127.4, 123.2, 121.1, 118.3, 116.0, 115.9, 111.3, 64.7, 63.4, 61.6, 55.5, 53.6, 50.8, 43.0, 25.6; ^19^F NMR (282 MHz, CDCl_3_) −113.4; [α]_D_^20^ = 48.1 (c 1.01, CHCl_3_); HRMS (ESI) [M + 1] found 457.2226 (calcd 457.2251); Chiral HPLC Chiralpak AS-H column (250 × 4.6 mm), hexane/i-PrOH = 80/20 (v/v) + 0.5% Et_3_N, flow rate: 0.8 mL/min, temperature 29 °C, detection at λ = 254 nm, minor: 17.8 min, minor: 19.9 min, minor: 25.0 min, main: 30.8 min, 79% *de*.

##### 4.1.2.5 (*R*)-3-((*S*)-3-(4-Benzhydrylpiperazin-1-yl)-2-hydroxypropyl)-5-(4-fluorophenyl)-5-methylimidazolidine-2,4-dione (**6.1**)

White solid (0.19 g, Yield 45%); C_30_H_33_FN_4_O_3_ (*M*_W_ 516.62); all spectroscopic data have been in full agreement with those reported for compound **2**; ^1^H NMR (600 MHz, CDCl_3_): δ 7.51–7.45 (m, 2H), 7.43–7.36 (m, 4H), 7.29–7.23 (m, 4H), 7.18 (dd, *J* = 7.4, 1.7 Hz, 2H), 7.09–7.02 (m, 2H), 5.94 (s, 1H), 4.19 (s, 1H), 3.96 (m, 1H), 3.61 (dd, *J* = 13.9, 7.6 Hz, 1H), 3.52 (dd, *J* = 13.9, 4.3 Hz, 1H), 2.79–2.14 (m, 10H), 1.82 (s, 3H); ^13^C NMR (151 MHz, CDCl_3_): δ 156.7, 142.8, 128.6, 128.0, 128.0, 127.4, 127.1, 116.0, 115.9, 76.3, 64.6, 63.3, 61.4, 52.0, 43.0, 25.6; ^19^F NMR (282 MHz, CDCl_3_): δ −113.4; [α]_D_^20^ = −39.9 (c 1.01, CHCl_3_); HRMS (ESI) [M + 1] found 517.2578 (calcd 517.2615); Chiral HPLC: Chiralpak OZ-H column (250 × 4.6 mm), hexane/i-PrOH = 80/20 (v/v), flow rate: 0.5 mL/min, temperature 30 °C, detection at λ = 254 nm, minor: 18.4 min, main: 30.1 min, minor: 40.8 min, 84% *de*.

##### 4.1.2.6 (*R*)-3-((*R*)-3-(4-Benzhydrylpiperazin-1-yl)-2-hydroxypropyl)-5-(4-fluorophenyl)-5-methylimidazolidine-2,4-dione (**6.2**)

White solid (0.24 g; Yield 51%); C_30_H_33_FN_4_O_3_ (*M*_W_ 516.62); all spectroscopic data have been in full agreement with those reported for compound **2**; ^1^H NMR (600 MHz, CDCl_3_): δ 7.51–7.44 (m, 2H), 7.38 (dt, *J* = 8.1, 1.4 Hz, 4H), 7.29–7.22 (m, 4H), 7.20–7.12 (m, 2H), 7.08–7.01 (m, 3H), 4.17 (s, 1H), 3.91 (tdd, *J* = 7.8, 5.6, 4.3 Hz, 1H), 3.56 (dd, *J* = 13.9, 7.8 Hz, 1H), 3.50 (dd, *J* = 13.9, 4.3 Hz, 1H), 2.73–2.15 (m, 10H), 1.79 (s, 3H); ^13^C NMR (151 MHz, CDCl_3_): δ 175.6, 163.6, 161.9, 157.2, 142.7, 134.6, 128.6, 128.0, 127.9, 127.4, 127.4, 127.0, 115.9, 115.8, 76.2, 65.9, 64.6, 63.3, 61.4, 53.6, 52.0, 43.0, 25.7, 15.4.; ^19^F NMR (282 MHz, CDCl_3_): δ −113.6; [α]_D_^20^ = −36.6 (c 1.02, CHCl_3_); HRMS (ESI) [M + 1] found 517.2578 (calcd 517.2615); Chiral HPLC: Chiralpak OZ-H column (250 × 4.6 mm), hexane/i-PrOH = 80/20 (v/v), flow rate: 0.5 mL/min, temperature 30 °C, detection at λ = 254 nm, minor: 18.6 min, minor: 21.8 min, minor: 30.8 min, main: 45.0 min, 88% *de*).

##### 4.1.2.7 (*S*)-3-((*S*)-3-(4-Benzhydrylpiperazin-1-yl)-2-hydroxypropyl)-5-(4-fluorophenyl)-5-methylimidazolidine-2,4-dione (**6.3**)

White solid (0.27 g; Yield 54%); C_30_H_33_FN_4_O_3_ (*M*_W_ 516.62); all spectroscopic data have been in full agreement with those reported for compound **2**; ^1^H NMR (600 MHz, CDCl_3_): δ 7.52–7.45 (m, 2H), 7.38 (dt, *J* = 8.1, 1.4 Hz, 4H), 7.25 (ddd, *J* = 7.9, 6.8, 1.7 Hz, 4H), 7.16 (td, *J* = 7.2, 1.5 Hz, 2H), 7.12 (s, 1H), 7.03 (dd, *J* = 9.6, 7.7 Hz, 2H), 4.17 (s, 1H), 3.96–3.87 (m, 1H), 3.56 (dd, *J* = 13.9, 7.7 Hz, 1H), 3.50 (dd, *J* = 13.9, 4.3 Hz, 1H), 2.73–2.13 (m, 10H), 1.78 (s, 3H); ^13^C NMR (151 MHz, CDCl_3_): δ 175.6, 163.6, 161.9, 157.2, 142.7, 134.6, 128.6, 128.0, 127.9, 127.4, 127.4, 127.1, 115.9, 115.8, 76.2, 65.9, 64.6, 63.3, 61.4, 53.6, 51.9, 43.0, 25.7, 15.4; ^19^F NMR (282 MHz, CDCl_3_): δ −113.6; [α]_D_^20^ = 36.3 (c 1.01, CHCl_3_); HRMS (ESI) [M + 1] found 517.2578 (calcd 517.2615); Chiral HPLC: Chiralpak OZ-H column (250 × 4.6 mm), hexane/i-PrOH = 80/20 (v/v), flow rate: 0.5 mL/min, temperature 30 °C, detection at λ = 254 nm, main: 18.4 min, minor: 20.6 min, minor: 30.9 min 94% *de*.

##### 4.1.2.8 (*S*)-3-((*R*)-3-(4-Benzhydrylpiperazin-1-yl)-2-hydroxypropyl)-5-(4-fluorophenyl)-5-methylimidazolidine-2,4-dione (**6.4**)

White solid (0.18 g; Yield 43%); C_30_H_33_FN_4_O_3_ (*M*_W_ 516.62; all spectroscopic data have been in full agreement with those reported for compound **2**; ^1^H NMR (600 MHz, CDCl_3_): δ 7.47 (dd, *J* = 8.9, 5.1 Hz, 2H), 7.39 (dd, *J* = 6.8, 1.5 Hz, 4H), 7.29–7.23 (m, 4H), 7.21–7.14 (m, 2H), 7.05 (dd, *J* = 8.9, 8.3 Hz, 2H), 6.16 (s, 1H), 4.18 (s, 1H), 3.95 (ddt, *J* = 9.7, 8.0, 4.1 Hz, 1H), 3.60 (dd, *J* = 13.9, 7.6 Hz, 1H), 3.52 (dd, *J* = 13.9, 4.2 Hz, 2H), 2.67–2.27 (m, 10H), 1.82 (s, 3H); ^13^C NMR (151 MHz, CDCl_3_): δ 156.8, 142.8, 128.6, 128.0, 128.0, 127.4, 127.4, 127.1, 116.0, 115.9, 76.3, 64.6, 63.3, 61.4, 52.1, 43.0, 25.6; ^19^F NMR (282 MHz, CDCl_3_) −113.5; [α]_D_^20^ = 41.8 (c 1.02, CHCl_3_); HRMS (ESI) [M + 1] found 517.2578 (calcd 517.2615); Chiral HPLC: Chiralpak OZ-H column (250 × 4.6 mm), hexane/i-PrOH = 80/20 (v/v), flow rate: 0.5 mL/min, temperature 30 °C, detection at λ = 254 nm, minor: 18.6 min, main: 21.6 min, 89% *de*.

### 4.2 X-ray Crystallographic Studies

Single crystals suitable for X-ray analysis were obtained from water/MeOH for **3.1** and **3.2** and water (precipitates from water phase during workup) for **5.1**–**5.4** by filtration of crystals from solution under air at rt and drying under vacuum.

Data for single crystals of **5.1–5.4** and **3.2** were collected at 100 K using an XtaLAB Synergy-S diffractometer, equipped with the Cu (1.54184 Å) Kα radiation source and graphite monochromator, while data for **3.1** were collected at 120 K using an Oxford Diffraction SuperNova four circle diffractometer, equipped with the Cu (1.54184 Å) Kα radiation source and graphite monochromator. The phase problems were solved by direct methods using SIR-2014^45^ and all non-hydrogen atoms were refined anisotropically using weighted full-matrix least-squares on F^2^. Refinement and further calculations were carried out using SHELXL.^46^ The hydrogen atoms bonded to carbon atoms were included in the structure at idealized positions and were refined using a riding model with *U*_iso_(H) fixed at 1.2 U_eq_ of C with the exception of hydrogen atoms in the methyl group for which U_iso_(H) was fixed at 1.5 U_eq_. Hydrogen atoms attached to nitrogen and oxygen atoms were found from the difference Fourier maps and refined without any restraints. The absolute structures were determined on the basis of the anomalous dispersion phenomenon. Compounds **5.1–5.4** crystallized, together with one water molecule. The oxygen atom of water molecule was disordered in all four crystal structures. The occupancy factors of the oxygen atoms were refined to be 0.58(6), 0.82(1), 0.61(6), and 0.57(7) for the major components for **5.1**, **5.2**, **5.3**, and **5.4**, respectively. For molecular graphics, the MERCURY^47^ program was used.

**5.1:** C_24_H_29_FN_4_O_4_·H_2_O, M_r_ = 474.53, crystal size = 0.42 × 0.25 × 0.08 mm^3^, orthorhombic, space group *P*2_1_2_1_2_1_, a = 6.4156(1) Å, b = 10.2480(1) Å, c = 35.2940(5) Å, V = 2320.48(5) Å^3^, Z = 4, T = 100(2)K, 45 310 reflections collected, 4417 unique reflections (R_int_ = 0.0949), R1 = 0.0364, w*R*2 = 0.0944 [I > 2σ(I)], R1 = 0.0379, w*R*2 = 0.1010 [all data], absolute structure parameter 0.05(6).

**5.2:** C_24_H_29_FN_4_O_4_·H_2_O, M_r_ = 474.53, crystal size = 0.42 × 0.25 × 0.08 mm^3^, orthorhombic, space group *P*2_1_2_1_2_1_, a = 6.5272(1) Å, b = 10.2186(1) Å, c = 34.8944(4) Å, V = 2327.42(5) Å^3^, Z = 4, T = 100(2)K, 46 096 reflections collected, 4429 unique reflections (R_int_ = 0.0610), R1 = 0.0294, w*R*2 = 0.0762 [I > 2σ(I)], R1 = 0.0302, w*R*2 = 0.0777 [all data], absolute structure parameter 0.07(5).

**5.3:** C_24_H_29_FN_4_O_4_·H_2_O, M_r_ = 474.53, crystal size = 0.47 × 0.22 × 0.14 mm^3^, orthorhombic, space group *P*2_1_2_1_2_1_, a = 6.4156(1) Å, b = 10.2555(1) Å, c = 35.3036(5) Å, V = 2322.81(5) Å^3^, Z = 4, T = 100(2)K, 46 556 reflections collected, 4426 unique reflections (R_int_ = 0.0486), R1 = 0.0269, w*R*2 = 0.0668 [I > 2σ(I)], R1 = 0.0277, w*R*2 = 0.0697 [all data], absolute structure parameter 0.01(4).

**5.4:** C_24_H_29_FN_4_O_4_·H_2_O, M_r_ = 474.53, crystal size = 0.38 × 0.22 × 0.06 mm^3^, orthorhombic, space group *P*2_1_2_1_2_1_, a = 6.5273(1) Å, b = 10.2189(1) Å, c = 34.8932(6) Å, V = 2327.44(6) Å^3^, Z = 4, T = 100(2)K, 45 043 reflections collected, 4508 unique reflections (R_int_ = 0.0639), R1 = 0.0329, w*R*2 = 0.0849 [I > 2σ(I)], R1 = 0.0346, w*R*2 = 0.0889 [all data], absolute structure parameter 0.08(6).

**3.1**: C_10_H_9_FN_2_O_2_, M_r_ = 208.19, crystal size = 0.45 × 0.20 × 0.08 mm^3^, orthorhombic, space group *P*2_1_2_1_2_1_, a = 6.1808(1) Å, b = 7.0695(1) Å, c = 21.6568(3) Å, V = 946.30(2) Å^3^, Z = 4, T = 120(2)K, 19 437 reflections collected, 1823 unique reflections (R_int_ = 0.0708), R1 = 0.0268, w*R*2 = 0.0686 [I > 2σ(I)], R1 = 0.0281, w*R*2 = 0.0701 [all data], absolute structure parameter −0.01(10).

**3.2**: C_10_H_9_FN_2_O_2_, M_r_ = 208.19, crystal size = 0.45 × 0.20 × 0.08 mm^3^, orthorhombic, space group *P*2_1_2_1_2_1_, a = 6.1762(1) Å, b = 7.0613(1) Å, c = 21.5976(3) Å, V = 941.91(2) Å^3^, Z = 4, T = 100(2)K, 17 259 reflections collected, 1800 unique reflections (R_int_ = 0.0482), R1 = 0.0240, w*R*2 = 0.0655 [I > 2σ(I)], R1 = 0.0244, w*R*2 = 0.0659 [all data], absolute structure parameter 0.04(6).

### 4.3 Radioligand Binding Studies

Cell pellets were thawed and homogenized in 10 vol of assay buffer using an Ultra Turrax tissue homogenizer and centrifuged twice at 35 000 g for 15 min at 4 °C, with incubation for 15 min at 37 °C in between. The composition of the assay buffers was as follows: for 5-HT_1A_R: 50 mM Tris HCl, 0.1 mM EDTA, 4 mM MgCl_2_, 10 μM pargyline and 0.1% ascorbate; for 5-HT_2A_R: 50 mM Tris HCl, 0.1 mM EDTA, 4 mM MgCl_2_ and 0.1% ascorbate; for 5- HT_7b_R: 50 mM Tris HCl, 4 mM MgCl_2_, 10 mM pargyline and 0.1% ascorbate; and for dopamine D_2_LR: 50 mM Tris HCl, 1 mM EDTA, 4 mM MgCl_2_, 120 mM NaCl, 5 mM KCl, 1.5 mM CaCl_2_ and 0.1% ascorbate. All assays were incubated in a total volume of 200 mL in 96-well microtiter plates for 1 h at 37 °C, except 5-HT_2A_R which was incubated at 27 °C. The process of equilibration was terminated by rapid filtration through Unifilter plates with a 96-well cell harvester, and radioactivity retained on the filters was quantified on a Microbeta plate reader (PerkinElmer, USA). For displacement studies, the assay samples contained as radioligands (PerkinElmer, USA): 2.5 nM [^3^H]-8-OH-DPAT (135.2 Ci/mmol) for 5-HT_1A_R; 1 nM [3H]-ketanserin (53.4 Ci/mmol) for 5- HT_2A_R; 0.8 nM [3H]-5- CT (39.2 Ci/mmol) for 5-HT_7_R or 2.5 nM [3H]-raclopride (72 Ci/mmol) for D_2_LR. Nonspecific binding was defined with 10 mM of 5-HT in 5-HT_7_R binding experiments, whereas 20 mM of mianserin, 10 mM of methiothepine or 10 mM of haloperidol were used in 5-HT_2A_R and D_2_L assays, respectively. Each compound was tested in triplicate at 7 concentrations (10^–10^–10^–4^ M). The inhibition constants (*K*_i_) were calculated using the Cheng–Prusoff equation.^48^ Results were expressed as means of at least two separate experiments.

### 4.4 Functional Assays

The functional activity of **5.4** and **6.4** on intracellular cAMP levels, studied in CHO cells which stably expressed the human 5-HT_7_ receptor was performed at Eurofins Cerep (2, rue du Professeur GARGOUÏL, B.P. 30001, 86 600 Celle**-**Lévescault, France).^49^ The cAMP concentration was measured using the HTRF method. Adenylate cyclase activity was expressed as the percentage of the maximal effect obtained with 300 nM serotonin. The compounds were tested at 7 concentrations (10^–12^–10^–6^ M) in the h5-HT_7_ antagonist effect.

### 4.5 Behavioral Tests

#### 4.5.1 Animals

The behavioral experiments were performed on male BalbC mice weighing 20–25 g, n = 333. The animals were purchased from the Animal House at the Faculty of Pharmacy, Jagiellonian University Medical College, Krakow, Poland. Mice were kept in groups of ten in Makrolon type 3 cages (dimensions 26.5 × 15 × 42 cm). The animals were kept in an environmentally controlled room (ambient temperature 22 ± 2 °C; relative humidity 50–60%; 12:12 light: dark cycle; and lights on at 8:00). They were allowed to acclimatize with the environment enriched by nesting material, tooth grinding material, houses, and toys for 1 week before commencement of the experiments. During acclimatization, mice were accustomed to the presence of female experimenters and their appropriate manner of animals’ restraint, i.e., cupping each separate mouse in open hands, according to the 3Rs recommendations (handling).^50,51^ Standard laboratory food (Sniff M-Z) and filtered water were freely available. All the experiments were conducted in the light phase between 9 AM and 2 PM. Experimental groups consisted of 6–10 randomly selected animals, depending on the experiment, and each animal was used only once. The experiments were performed by an observer who was unaware of the treatment administered. All the experimental procedures with mice were carried out under EU Directive 2010/63/EU and were approved by the Local Ethics Commission for Animal Experiments of Jagiellonian University in Krakow (Approval nos.:297/2019).

#### 4.5.2 Drug Administration

The investigated compounds (**1**, **5.4**, **2**, and **6.4**) were suspended in 1% aqueous solution of Tween 80. Compounds were injected once intraperitoneally (*i.p*.) 60 min before tests, at a volume of 10 mL/kg. Control animals received a vehicle injection according to the same schedule.

#### 4.5.3 FST in Mice

The FST experiment was carried out according to the method of Porsolt et al.^52^ The total duration of immobility was recorded during the last 4 min of this 6 min period. The reduction of immobility time compared to the control was a measure of antidepressant properties of the tested compound. A mouse was regarded as immobile when it remained floating on water, making only small movements to keep its head above it.

#### 4.5.4 FPT in Mice

The four-plate apparatus (Bioseb, Pinellas Park, USA) was used for tests. Following a 15 s habituation period, the animal’s motivation to explore a novel environment is suppressed by an electric foot shock (0.8 mA, 0.5 s) every time it moves from one plate to another during a 1 min test session. This action is referred to as a “punished crossing” and is followed by a 3 s shock interval, during which the animal can move across plates without receiving a shock.^53^

#### 4.5.5 Spontaneous Locomotor Activity in Mice

Locomotor activity was recorded with an Opto M3 multichannel activity monitor (Columbus Instruments, Columbus, USA). The BalbC mice were individually placed in plastic cages (22 × 12 × 13 cm) for a 30 min habituation period, and then, the mice were observed during a 60 min session. Moreover, the mobility data of mice were collected also during the first minute and from 2 to 6 min of the test, i.e., the times equal to the observation periods in FPT and FST, respectively. According to the 3Rs recommendations,^54^ locomotor activity was assessed for the minimal active doses obtained in FST and/or FPT to ensure that detected antidepressant-/anxiolytic-like effects are not the result of increased motility of mice.

#### 4.5.6 Data Analysis and Statistics

Results are presented as means ± SEM. The comparisons between experimental and control groups were performed by a one-way ANOVA followed by the Bonferroni test post hoc. A value of *p* ≤ 0.05 was considered to be significant.

### 4.6 Molecular Modeling Studies

Possible interactions of four stereoisomers of **2** with 5-HT_7_R were modeled with the use of docking and MD simulations (for comparison, stereoisomers of MF-8 were also included in the modeling task). The recently released crystal structure of 5-HT_7_R was used (PDB ID: 7XTC),^55^ but as the modeled compounds display antagonistic mode of action (7XTC is cocrystallized with 5-CT), the 5-HT_7_R agonist, also the inactive form of 5-HT_7_R was used for modeling (inactive-state model from GPCRdb was used; despite the homology model validation provided by the GPCRdb service, we also performed the manual analysis of the orientation of crucial amino acids from the 5-HT_7_R binding pocket).

The Schrödinger Suite modeling package was used in the study. The compounds were prepared for docking using LigPrep^56^ (protonation states were generated for the pH = 7.4), protein preparation was carried out in the Protein Preparation Wizard,^57^ docking was carried out in Glide^58^ (in the “extra precision” mode), and MD simulations were conducted in Desmond. TIP3P^59^ was used as a solvent model and POPC (palmitoyl-oleoyl-phosphatidylcholine) was used as a membrane model; OPLS3e force-field under the pressure of 1.01325 bar at a temperature of 300 K was applied. The box shape was orthorhombic, with a size of 10 Å × 10 Å x 10 Å. In each case, the system was neutralized by the addition of the respective number of Cl- ions and relaxed before the simulation; the duration of each simulation was equal to 2000 ns.

The validity of the homology model used in the study was verified by examining its ability to discriminate active compounds from the inactive ones. It was done via two experiments: differentiation between active (*K*_*i*_ below 100 nM) and inactive (*K*_*i*_ above 1000 nM) compounds deposited in the ChEMBL database and differentiation between active compounds from ChEMBL database (*K*_*i*_ below 100 nM) and decoys generated according to the Directory of Useful Decoys^60^ methodology in a number of 2000. The above-mentioned compound sets were docked to the 5-HT_7_R homology model used in the study and its discriminative power was assessed via the BEDROC parameter^61^ (α = 20). The obtained BEDROC values of 0.688 for differentiation between active and inactive compounds from the ChEMBL database and 0.760 for the problem of discrimination between actives and decoys confirm the validity of the protein used for the study, providing its ability to correctly assess compounds in terms of their 5-HT_7_R affinity.

## Supporting Information Available

Detailed spectral characterization (^1^H, ^13^C NMR spectra and HPLC data), supporting data from MD simulations, and results from screening toward GABA and NK_1_ activity (PDF)
