# Supplementary material for: The Importance of Stereochemistry in 5-HT7R Modulation—A Case Study of Hydantoin Derivatives
Source: ACS Chem Neurosci. 2024 Oct 21;15(21):3884–900. doi: 10.1021/acschemneuro.4c00152 (PMC11587507; doi:10.1021/acschemneuro.4c00152)
Supplement: Supplementary file 1 — cn4c00152_si_001.pdf [file cn4c00152_si_001.pdf]

The importance of stereochemistry in 5-HT$_7$R modulation - a case study of hydantoin derivatives

Katarzyna Kucwaj-Brysz[a*], Sebastian Baś[b], Ewa Żesławska[c], Sabina Podlewska[d], Magdalena Jastrzębska-Więsek[e], Anna Partyka[e], Wojciech Nitek[b], Grzegorz Satała[d], Anna Wesołowska[e], Jadwiga Handzlik[a*]

[a]*Department of Technology and Biotechnology of Drugs, Jagiellonian University Medical College, Medyczna 9, 30-688 Cracow, Poland*

[b]*Faculty of Chemistry, Jagiellonian University, Gronostajowa 2, 30-387 Cracow, Poland*

[c]*Institute of Biology, Pedagogical University of Cracow, Podchorążych 2, 30-084 Cracow, Poland*

[d]*Department of Medicinal Chemistry, Maj Institute of Pharmacology, Polish Academy of Sciences, Smętna 12, 31-343 Cracow, Poland*

[e]*Department of Clinical Pharmacy, Jagiellonian University, Medical College, Medyczna 9, PL 30-688 Cracow, Poland;*

*An author to whom correspondence should be addressed; e-mail: katarzyna.kucwaj@uj.edu.pl

Content

1. *In vitro* evaluation towards another potentially related protein targets………………S2
2. Molecular modelling - additional data from molecular dynamic simulation………………………………………………………………………S3
3. Spectroscopic data for intermediate and final compounds…………………………………………………………………..…… S4
4. The extended NMR studies to elucidate the the differences between expected number of protons/carbons and observed in spectra…………………………...……………S35
5. HPLC data for intermediate and final compounds…………………………………S43

1. *In vitro* evaluation towards another related protein target (GABA, NK_1)

Table S2. *Affinity results to GABA and NK_1*

Compound	NK_1 Screening at 1 μM (% inhibition of control agonist response)	GABA (non-specific) Screening at 1 μM (% of inhibtion of control specific binding)
**5.4**	2.9	7
**6.4**	-4.4	26

[a]data are presented as the mean ± SD from two independent measurements

The above biological screening have been performed at Eurofins Cerep (2, rue du Professeur GARGOUÏL, B.P. 30001, 86 600 Celle l'Evescault, France according to previously described methods:

Tsuji, A. et al. (**1988**), *Antimicrob. Agents Chemother.,* 32*: 190-194.*

Eistetter, H.R. et al. (**1992**), *Glia,* 6*: 89-95.*

Detailed parameters for NK_1 assay:

Assay	Source	Stimulus	Incubation	Measured Component	Detection Method
**Receptors**					
**NK_1 *(h)* (antagonist effect)**	human endogenous (U373 cells)	$[Sar^9,Met(O_2)^{11}]$-SP (1 nM)	RT	intracellular $[Ca^{2+}]$	Fluorimetry

Detailed parameters for GABA (non-specific) assay:

Assay	Source	Ligand	Conc.	Kd	Non Specific	Incubation	Detection Method
**Receptors**							
**GABA (non-selective) (agonist radioligand)**	rat cerebral cortex	$[^3H]$GABA	10 nM	15 nM	GABA (100 μM)	60 min RT	Scintillation counting

2. Molecular modelling – additional data from molecular dynamic simulation

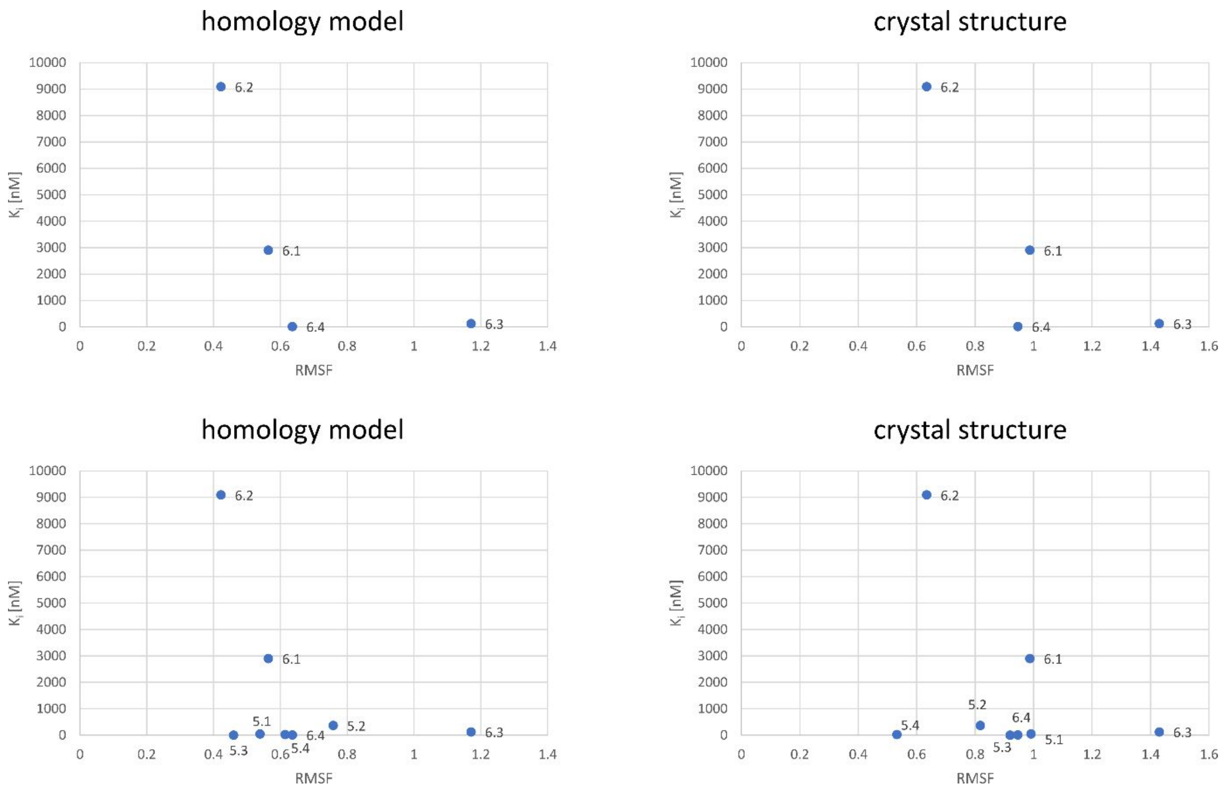

Figure S1. Correlation between the ligand RMSF during molecular dynamics simulations and their affinity towards 5-HT_7R.

3. Spectroscopic data for intermediate and final compounds

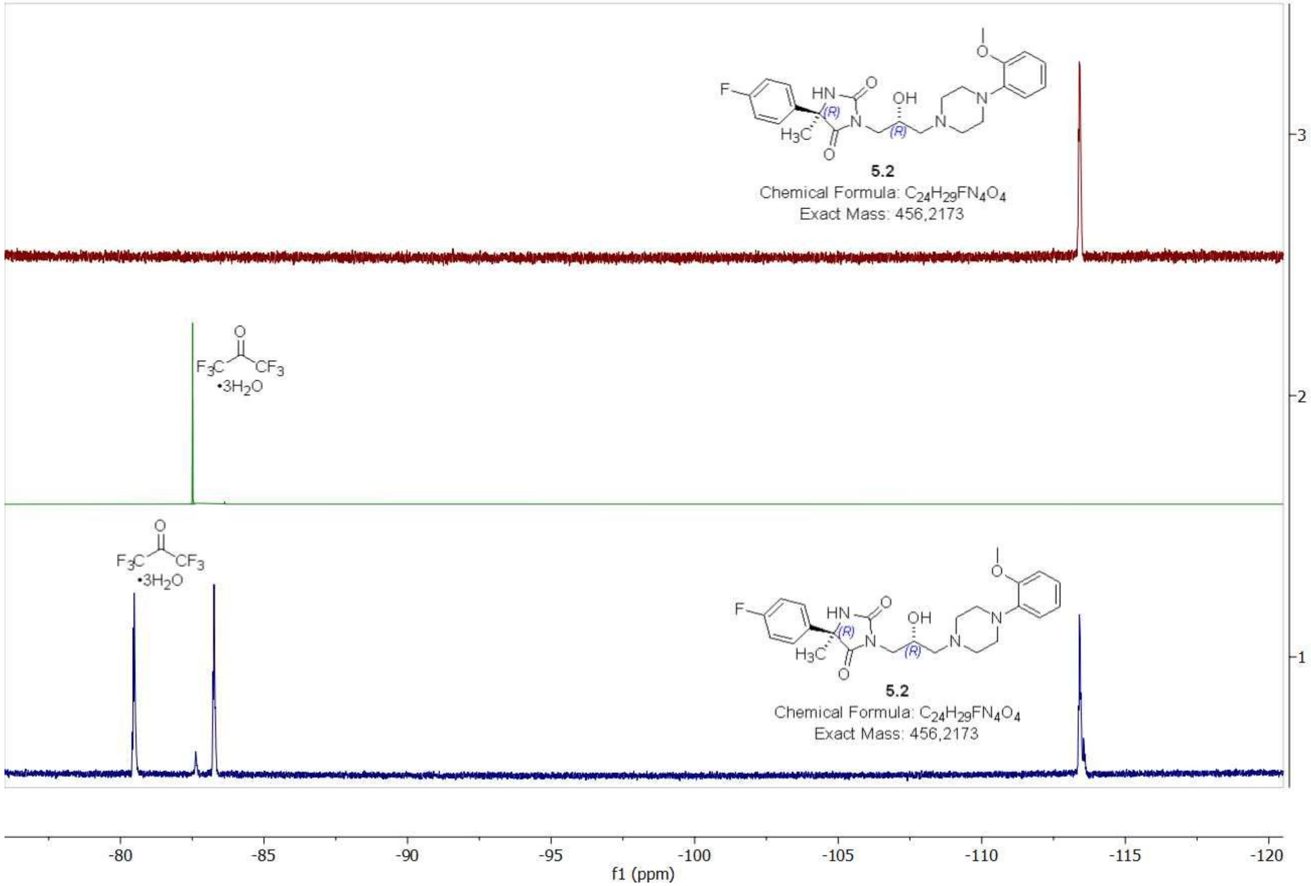

Figure S2. The confirmation of side reaction between reference compound hexafluoroacetone trihydrate and compound **5.2**. *Top* ^{19}F NMR spectra of pure compound **5.2**, *middle* ^{19}F NMR spectra of pure hexafluoroacetone trihydrate, *bottom* ^{19}F NMR spectra of mixture of hexafluoroacetone trihydrate and **5.2**.

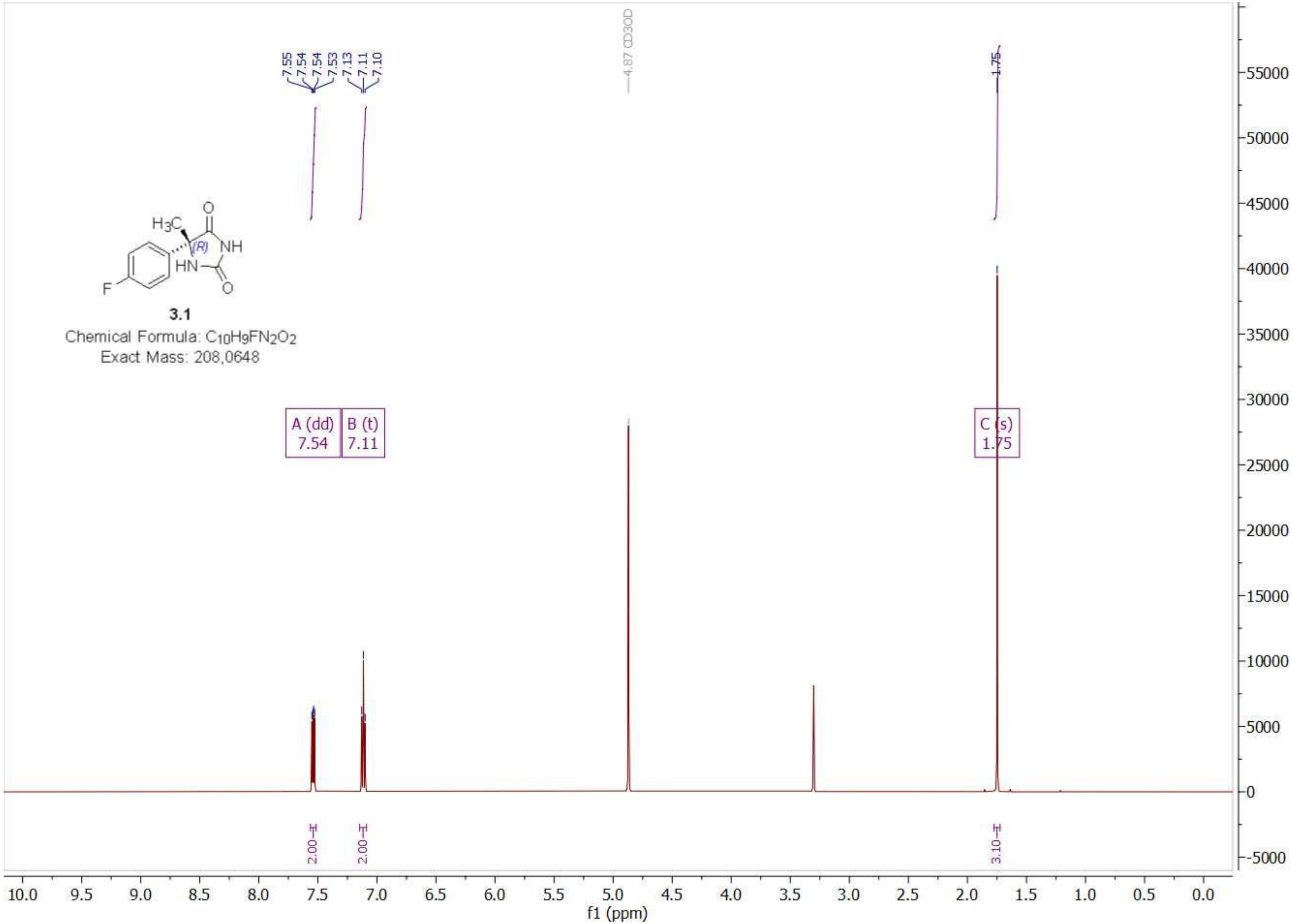

Figure S3. The ^{1}H NMR spectra of pure compound **3.1**

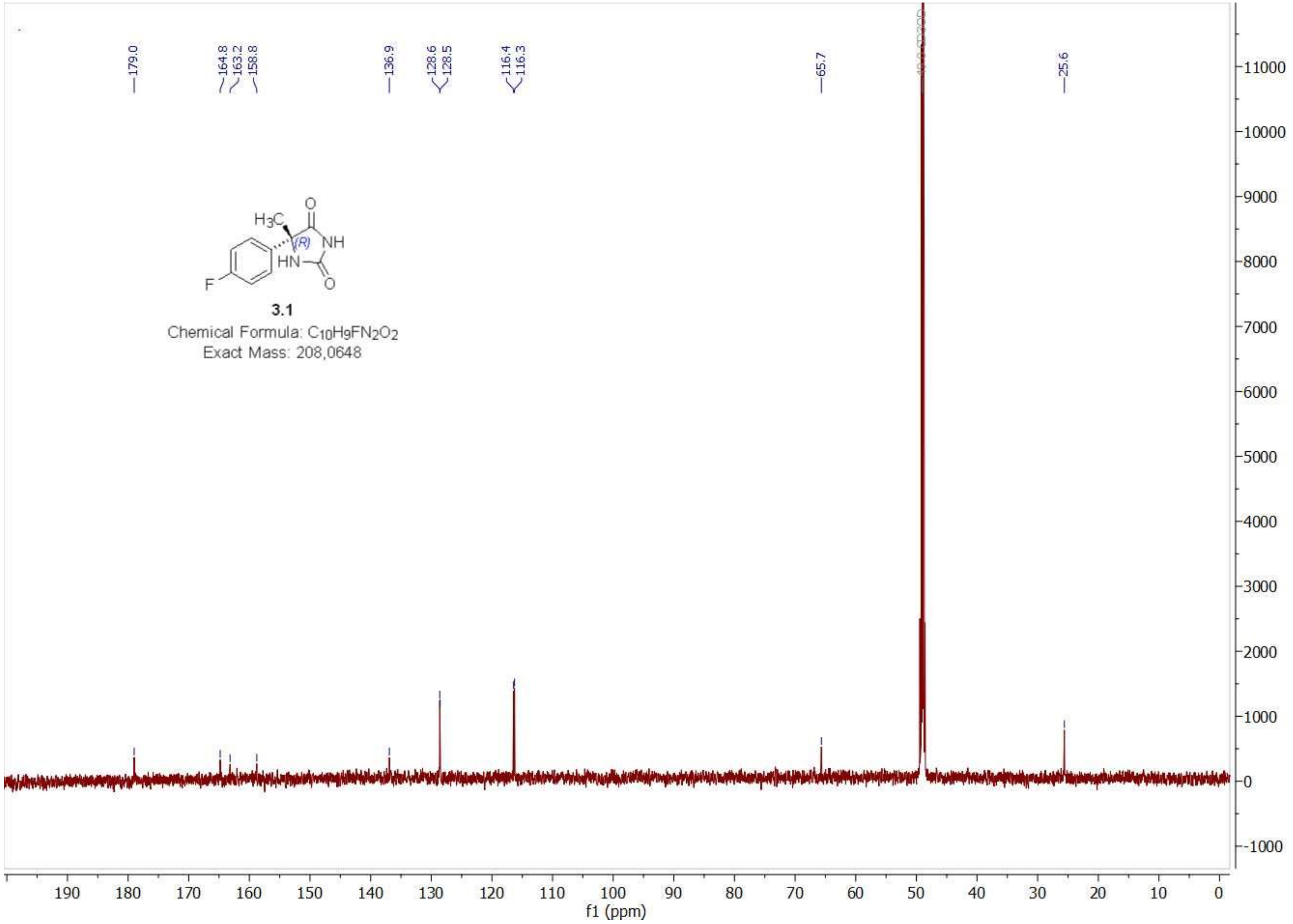

Figure S4. The ^{13}C NMR spectra of pure compound **3.1**

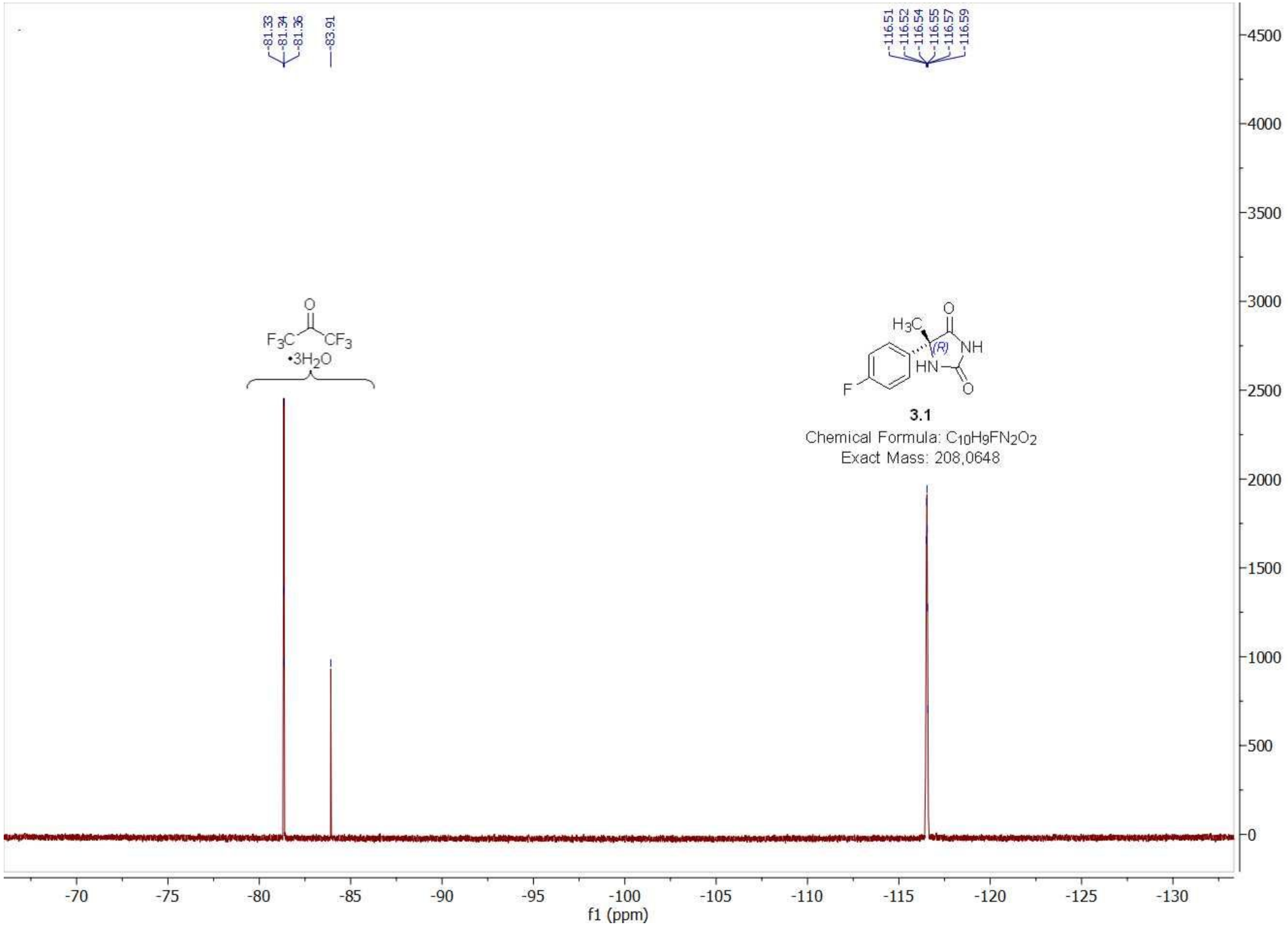

Figure S5. The ^{19}F NMR spectra of pure compound **3.1**

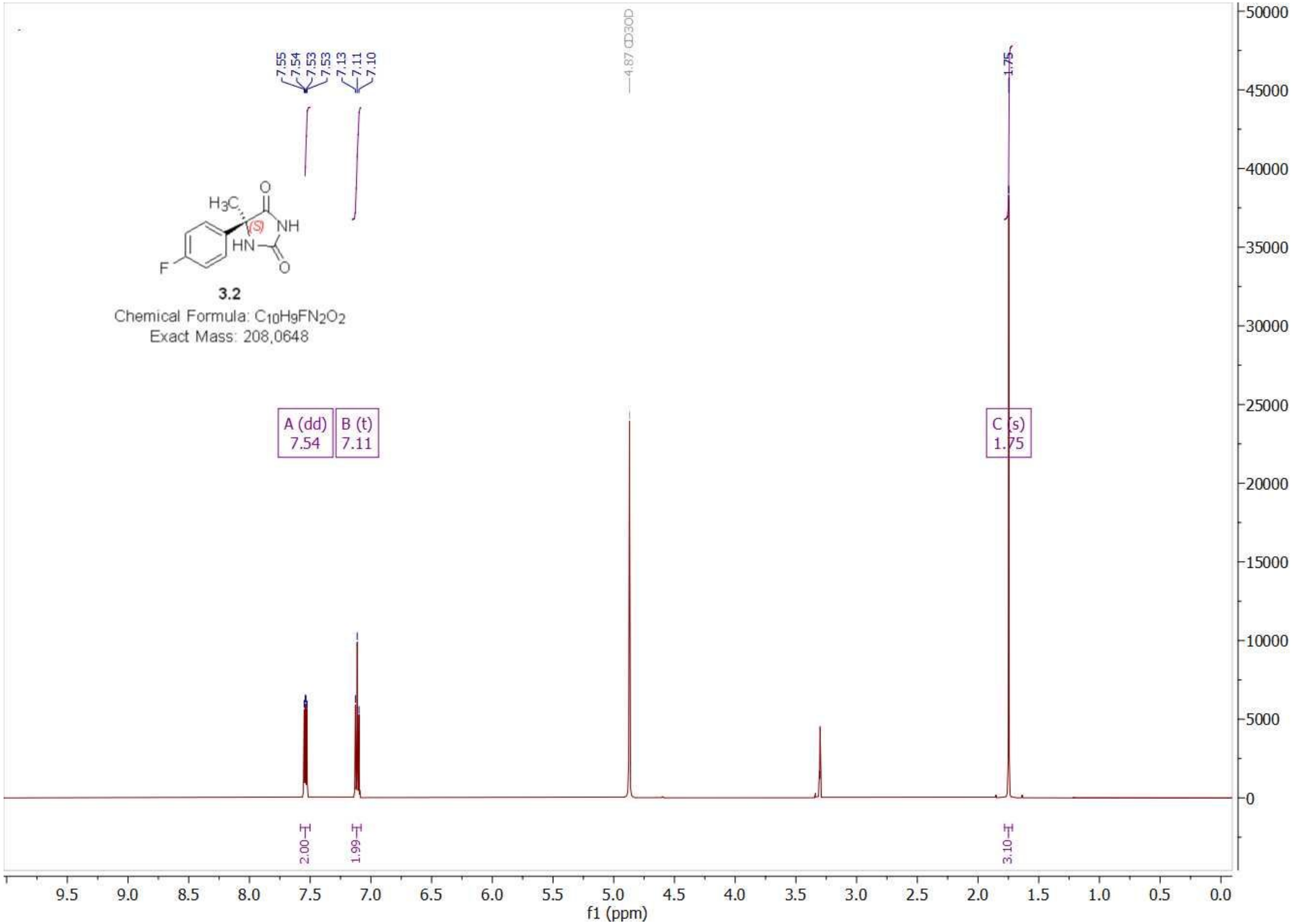

Figure S6. The ^{1}H NMR spectra of pure compound **3.2**

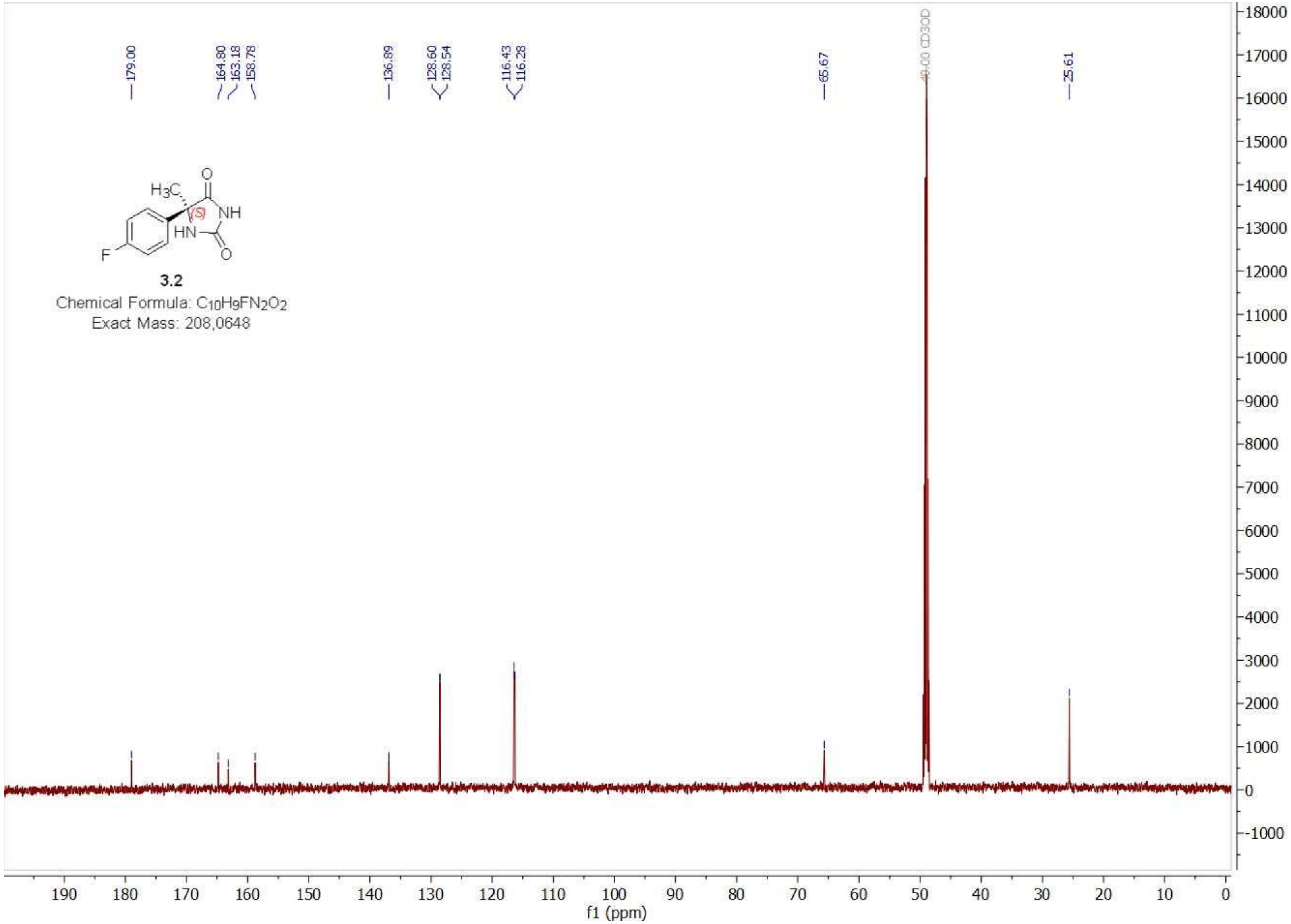

Figure S7. The ^{13}C NMR spectra of pure compound **3.2**

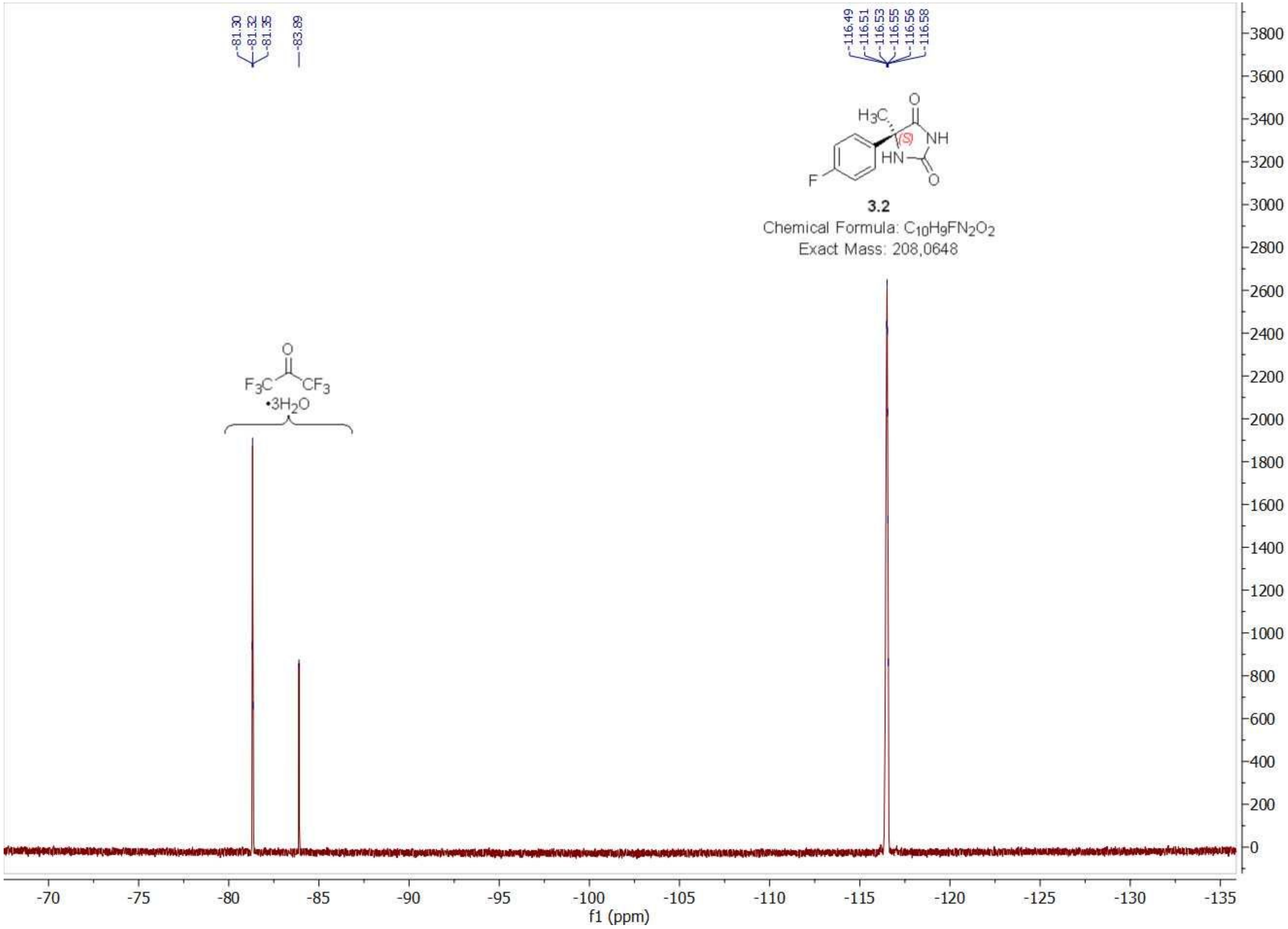

Figure S8. The ^{19}F NMR spectra of pure compound **3.2**

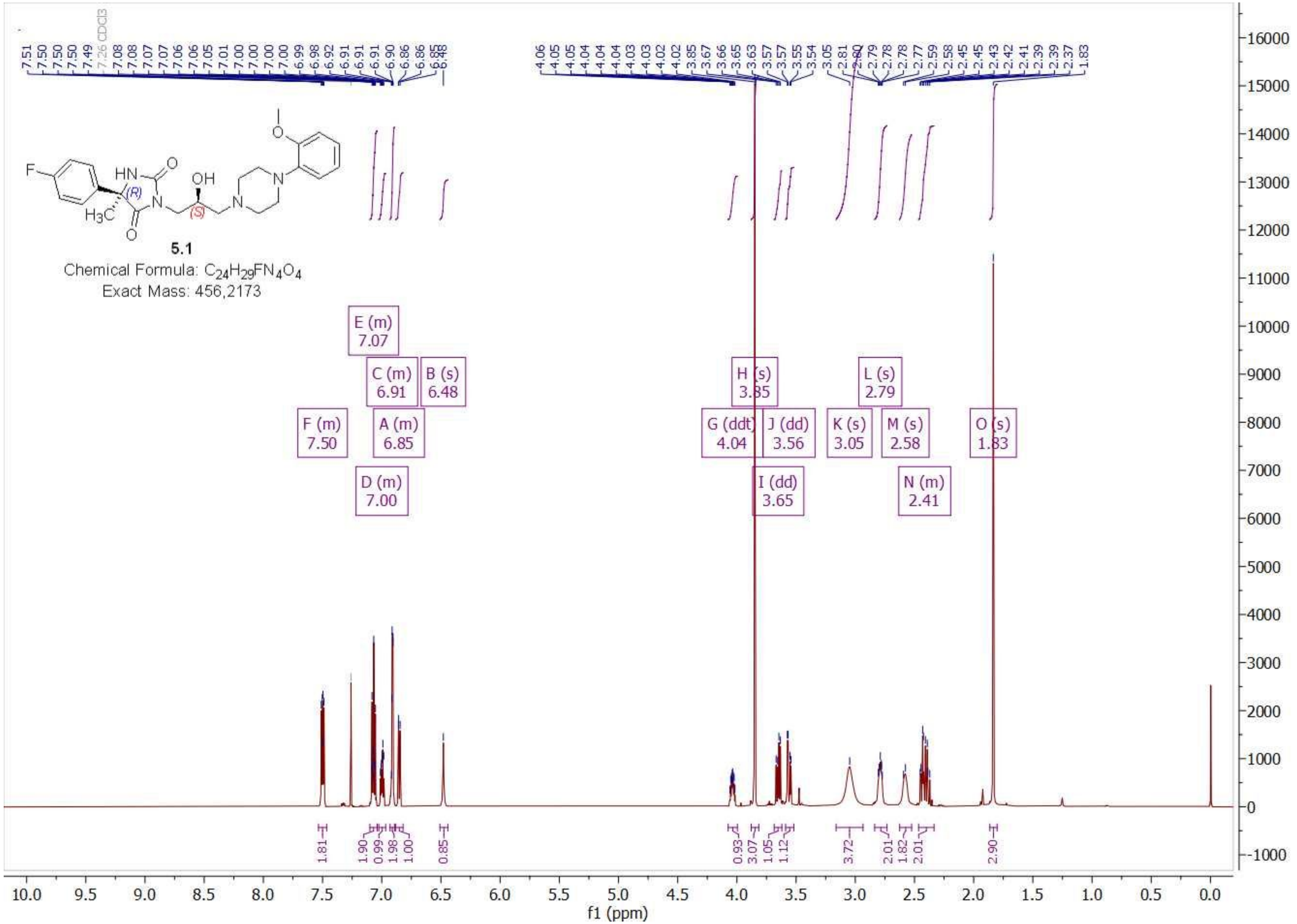

Figure S9. The ^{1}H NMR spectra of pure compound **5.1**

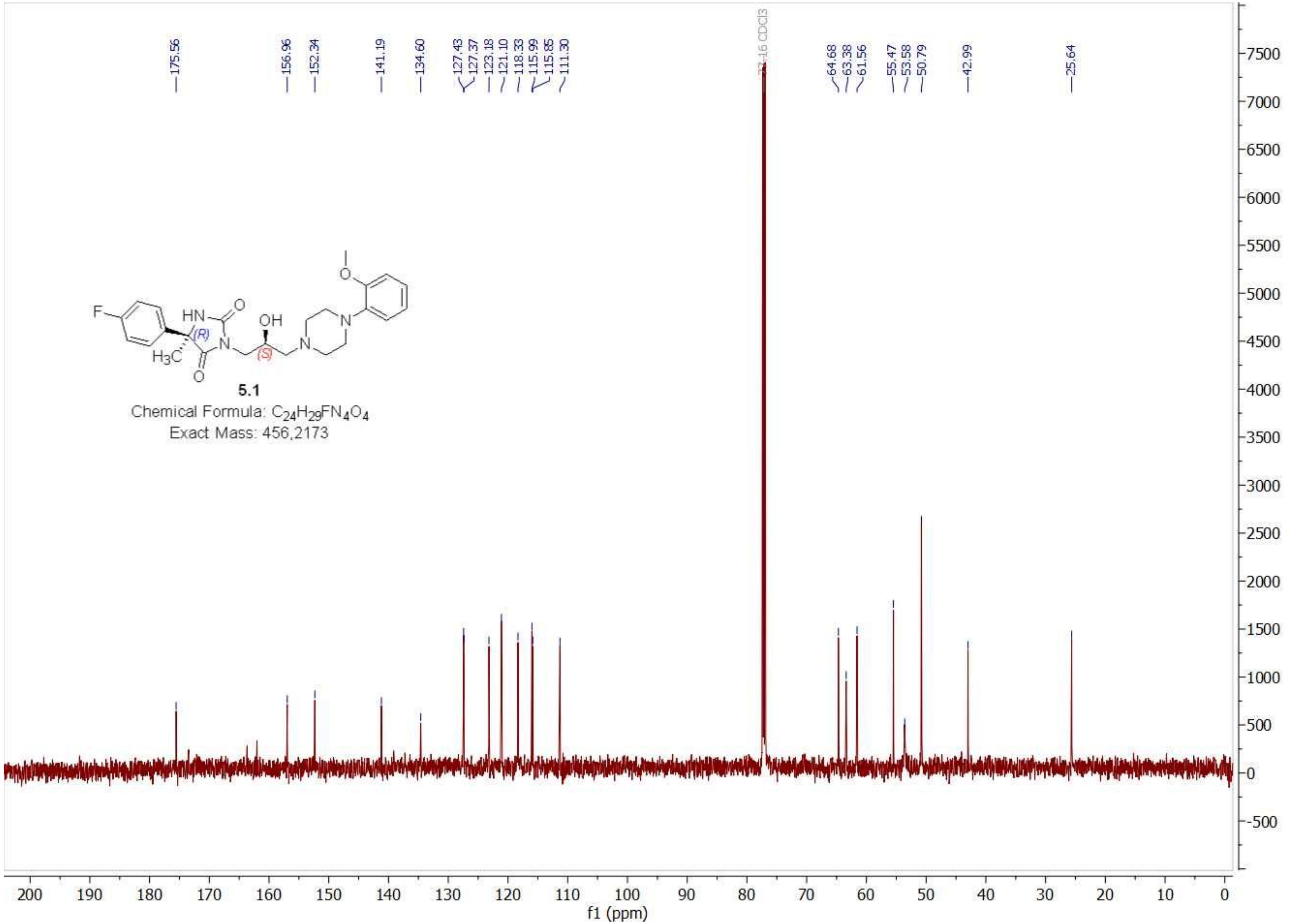

Figure S10. The ^{13}C NMR spectra of pure compound **5.1**

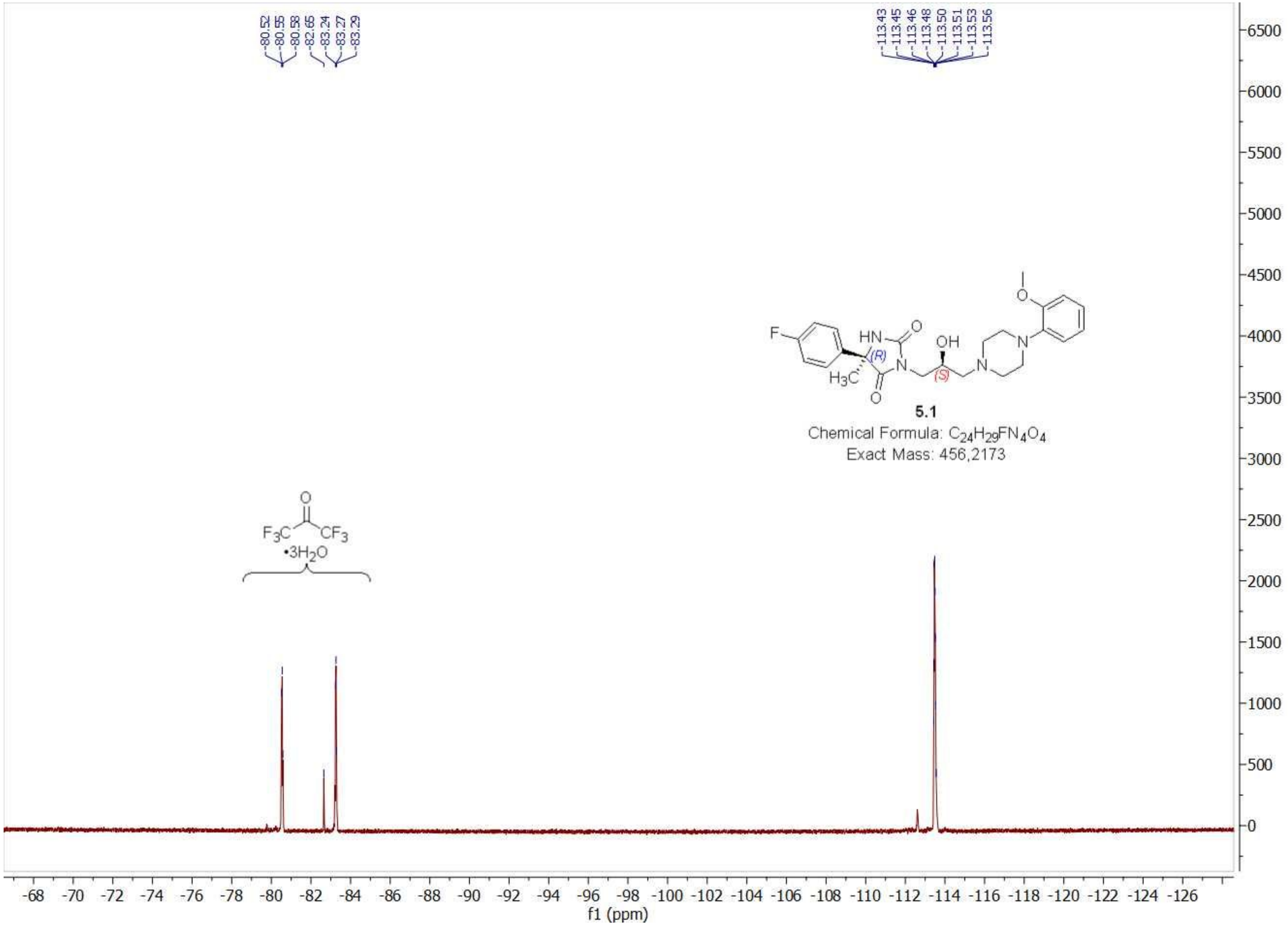

Figure S11. The ^{19}F NMR spectra of pure compound **5.1**

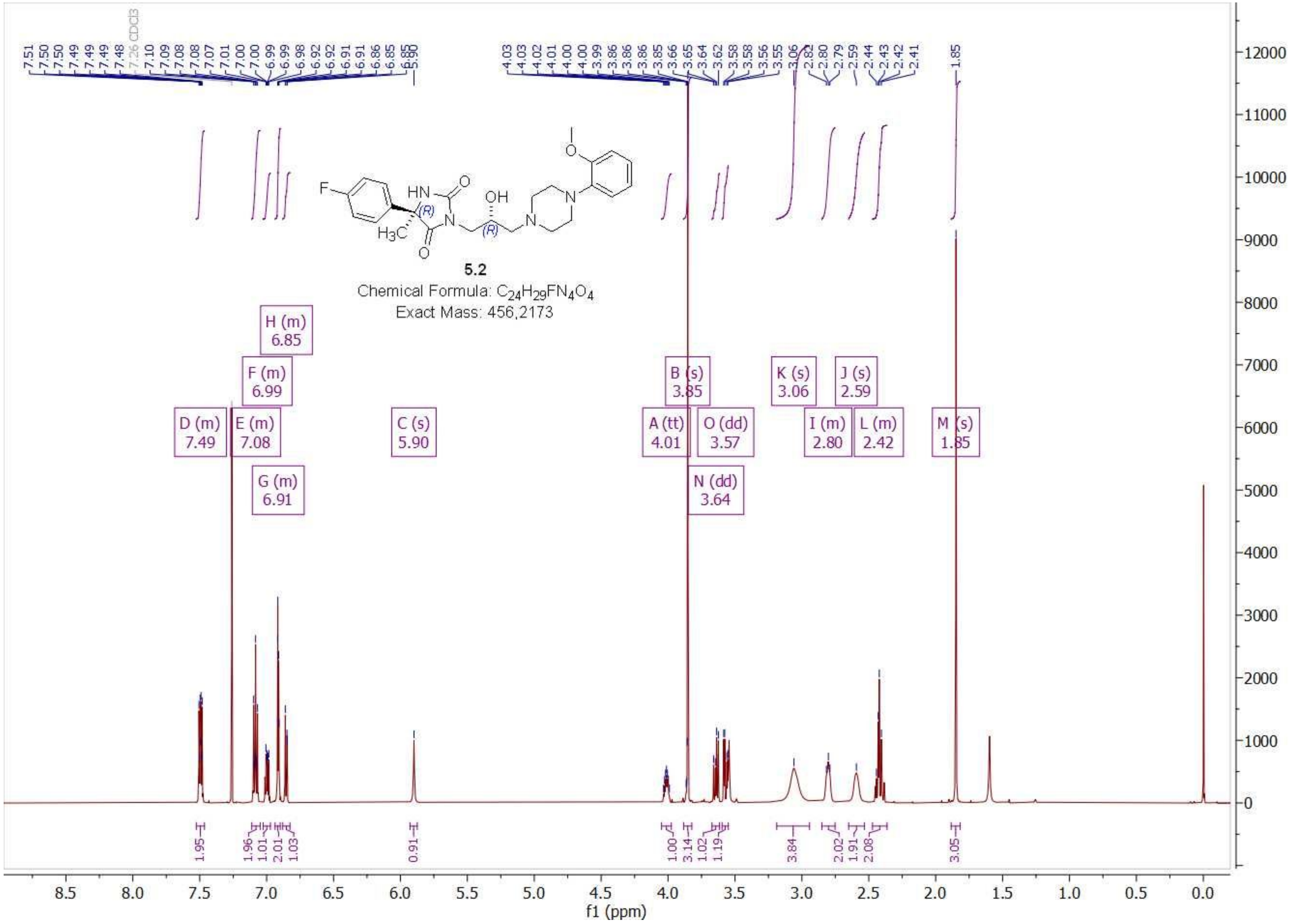

Figure S12. The ^{1}H NMR spectra of pure compound **5.2**

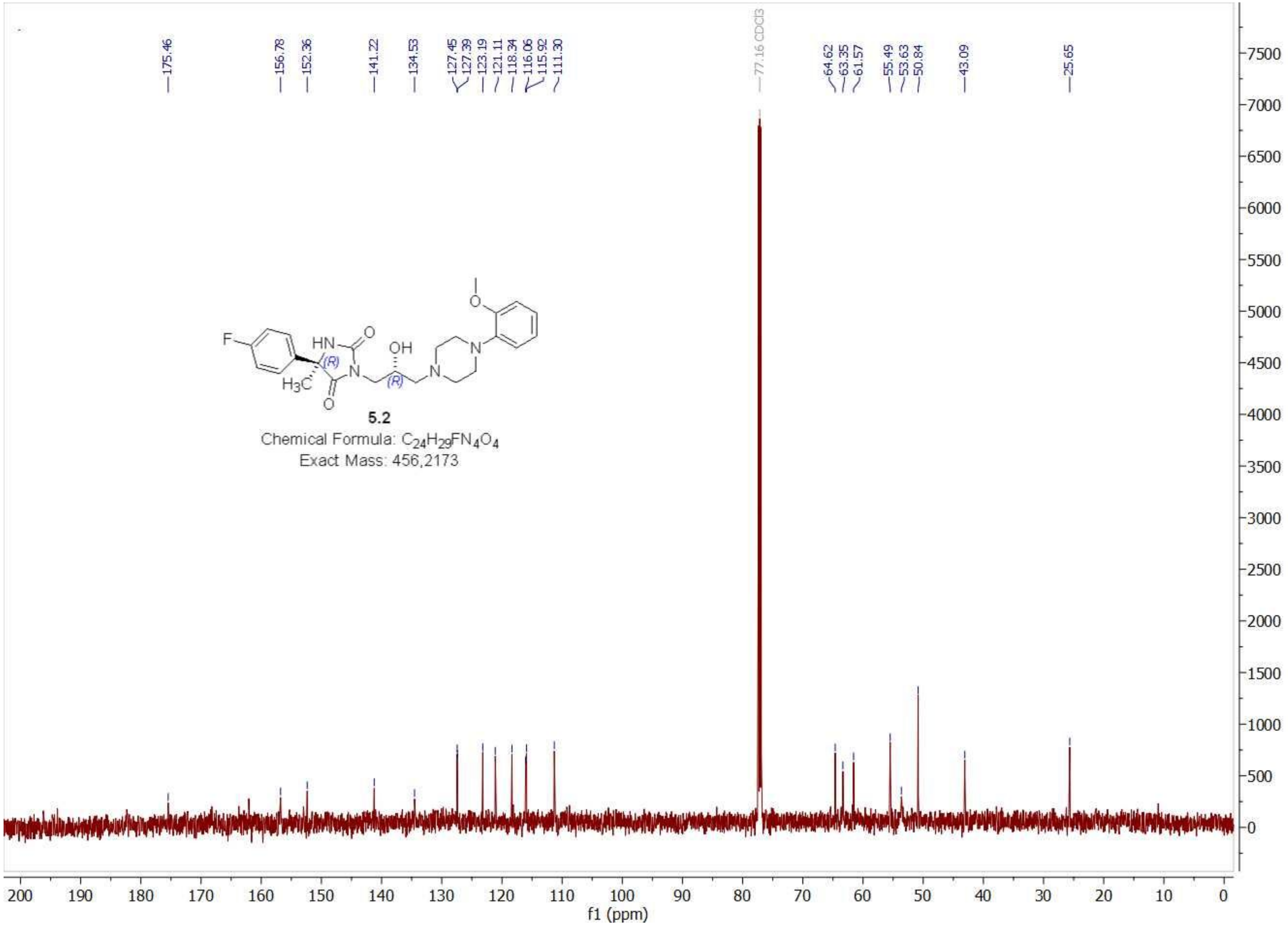

Figure S13. The ^{13}C NMR spectra of pure compound **5.2**

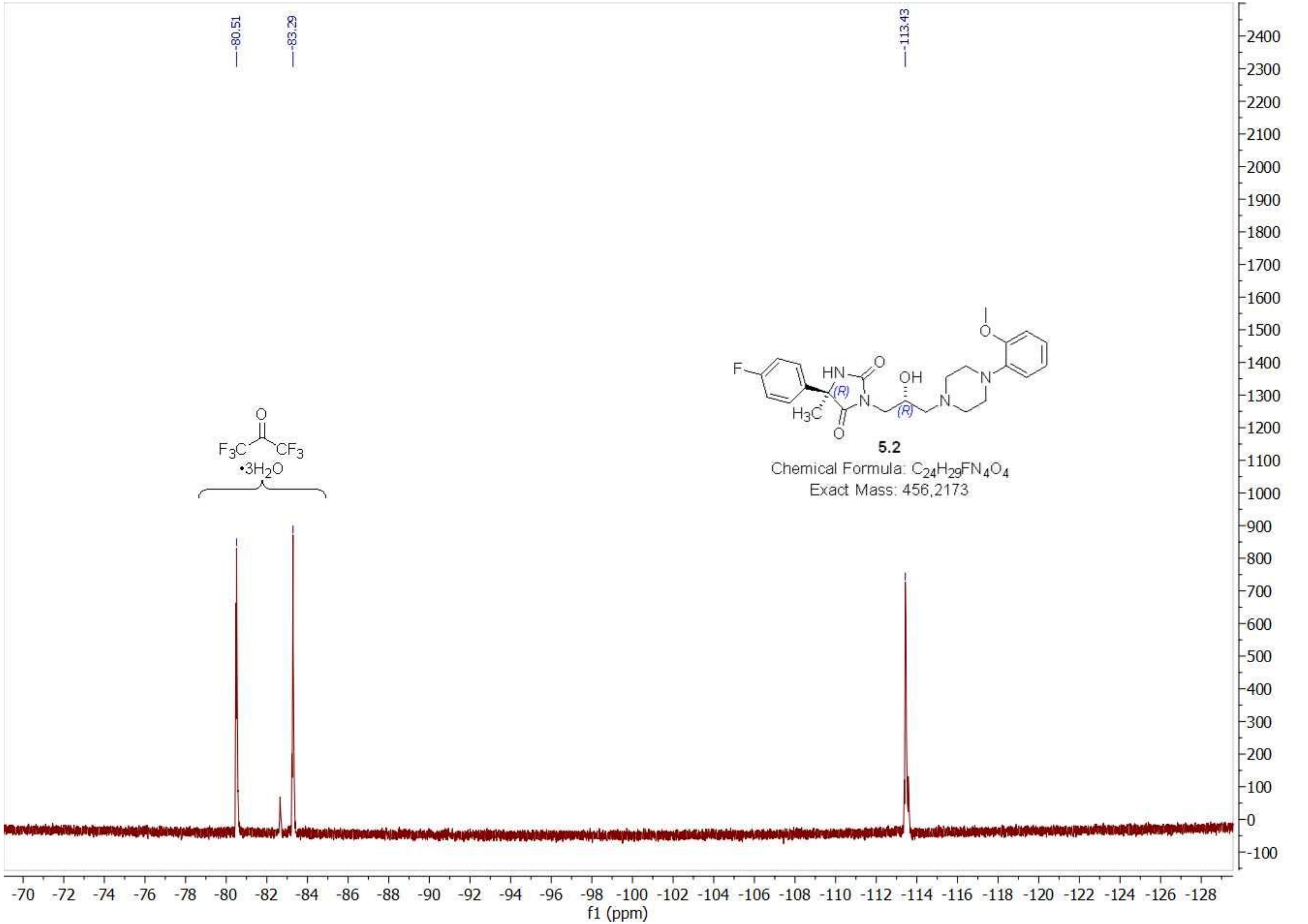

Figure S14. The ^{19}F NMR spectra of pure compound **5.2**

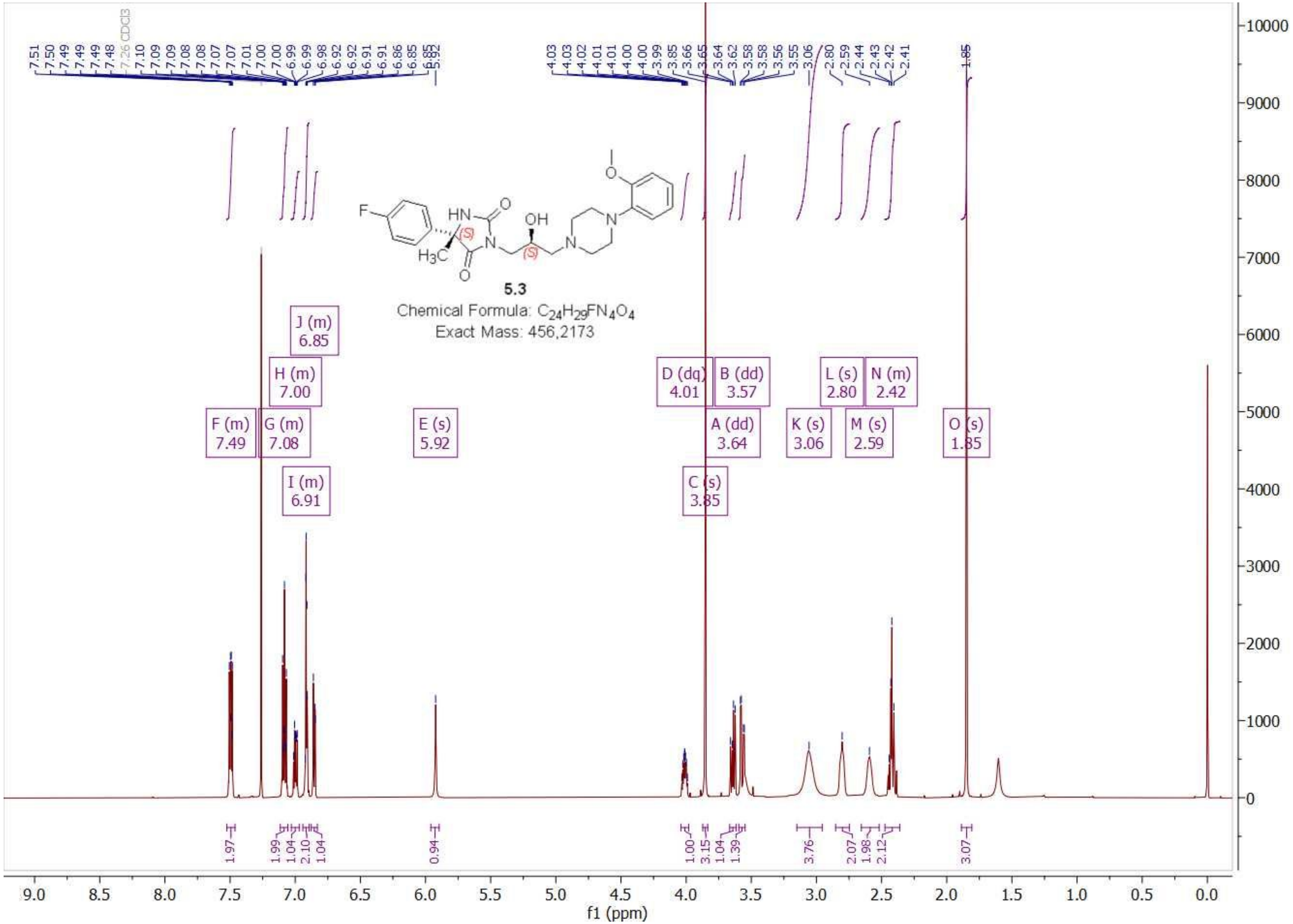

Figure S15. The ^{1}H NMR spectra of pure compound **5.3**

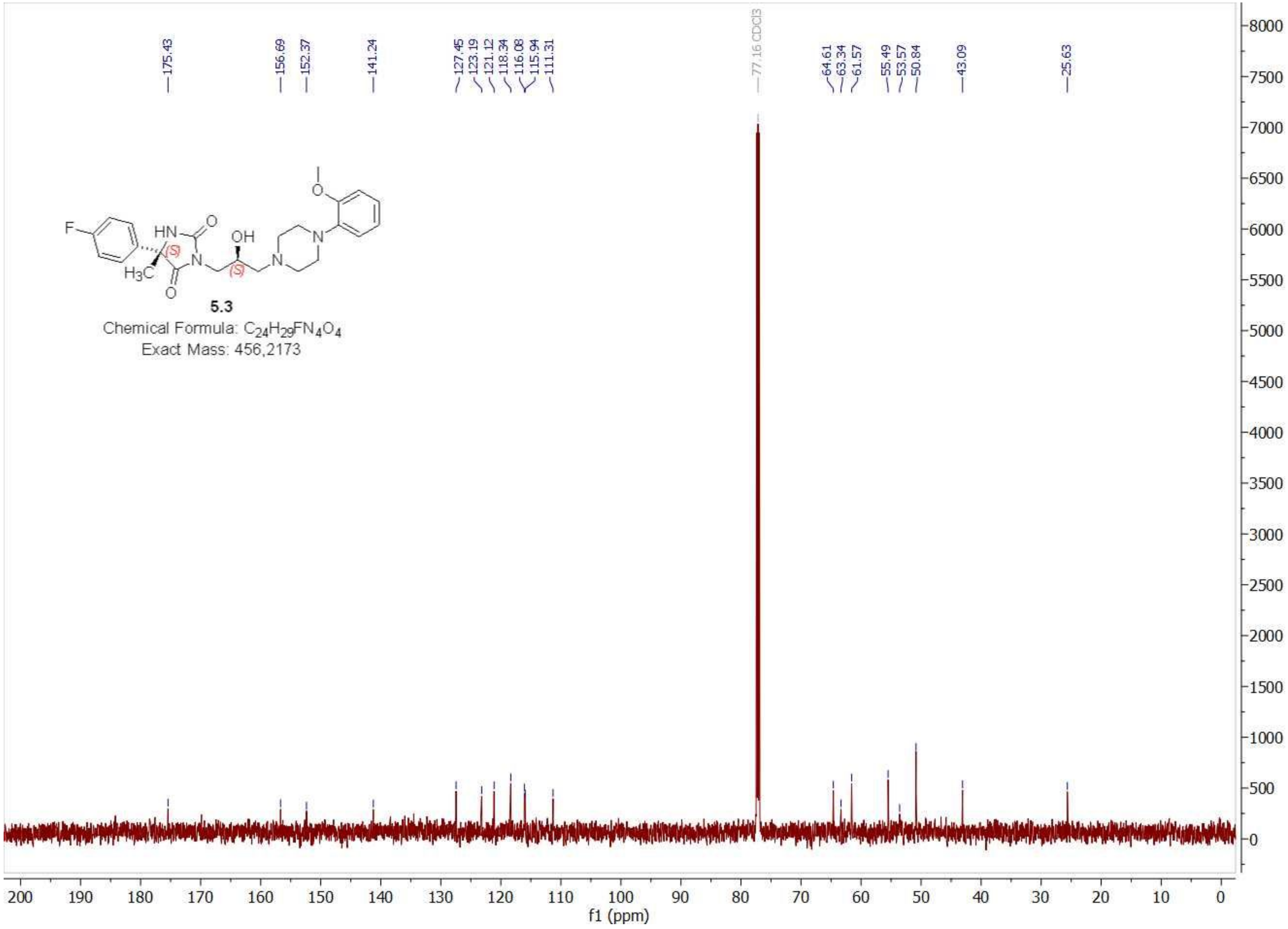

Figure S16. The ^{13}C NMR spectra of pure compound **5.3**

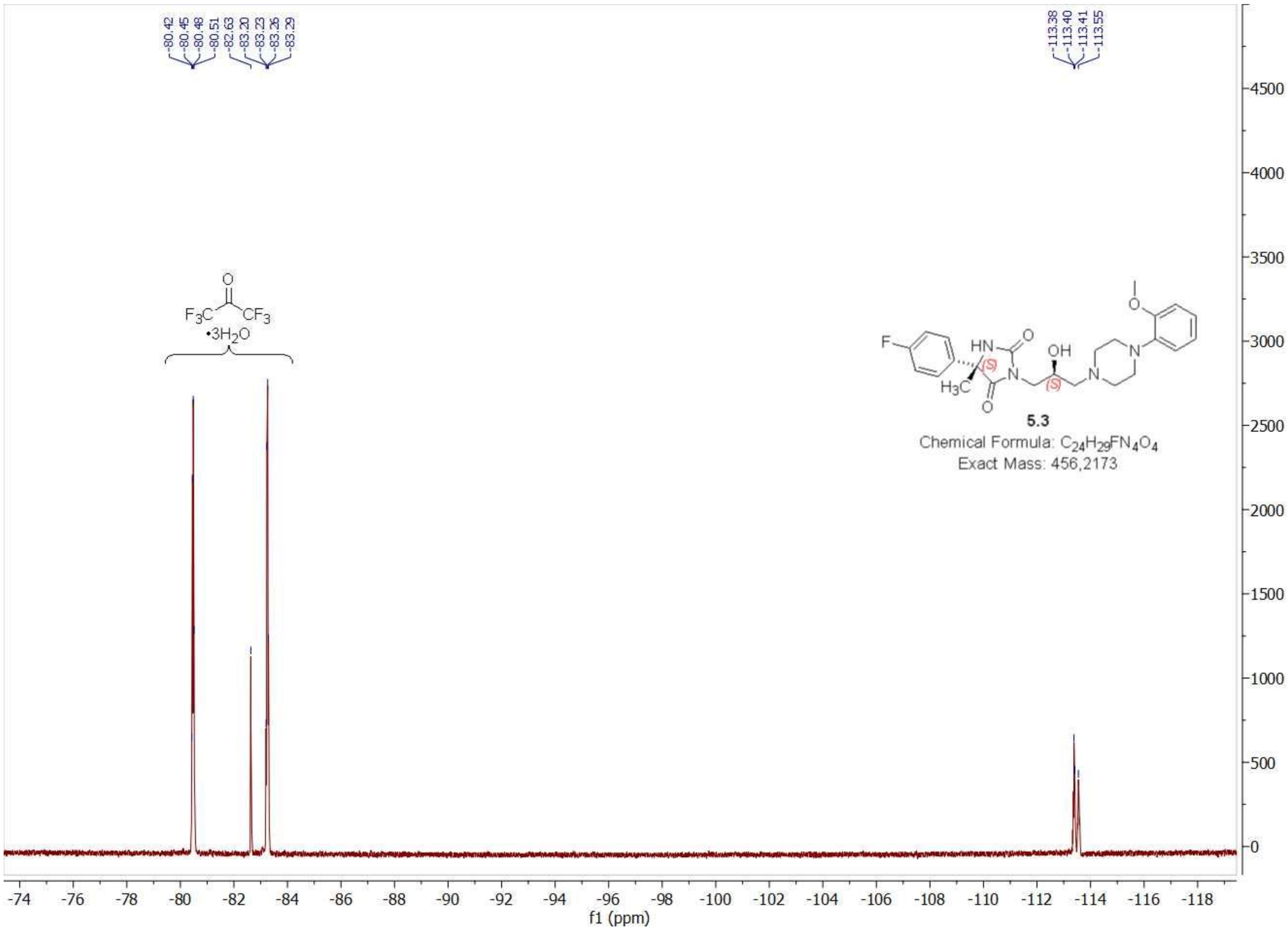

Figure S17. The ^{19}F NMR spectra of pure compound **5.3**

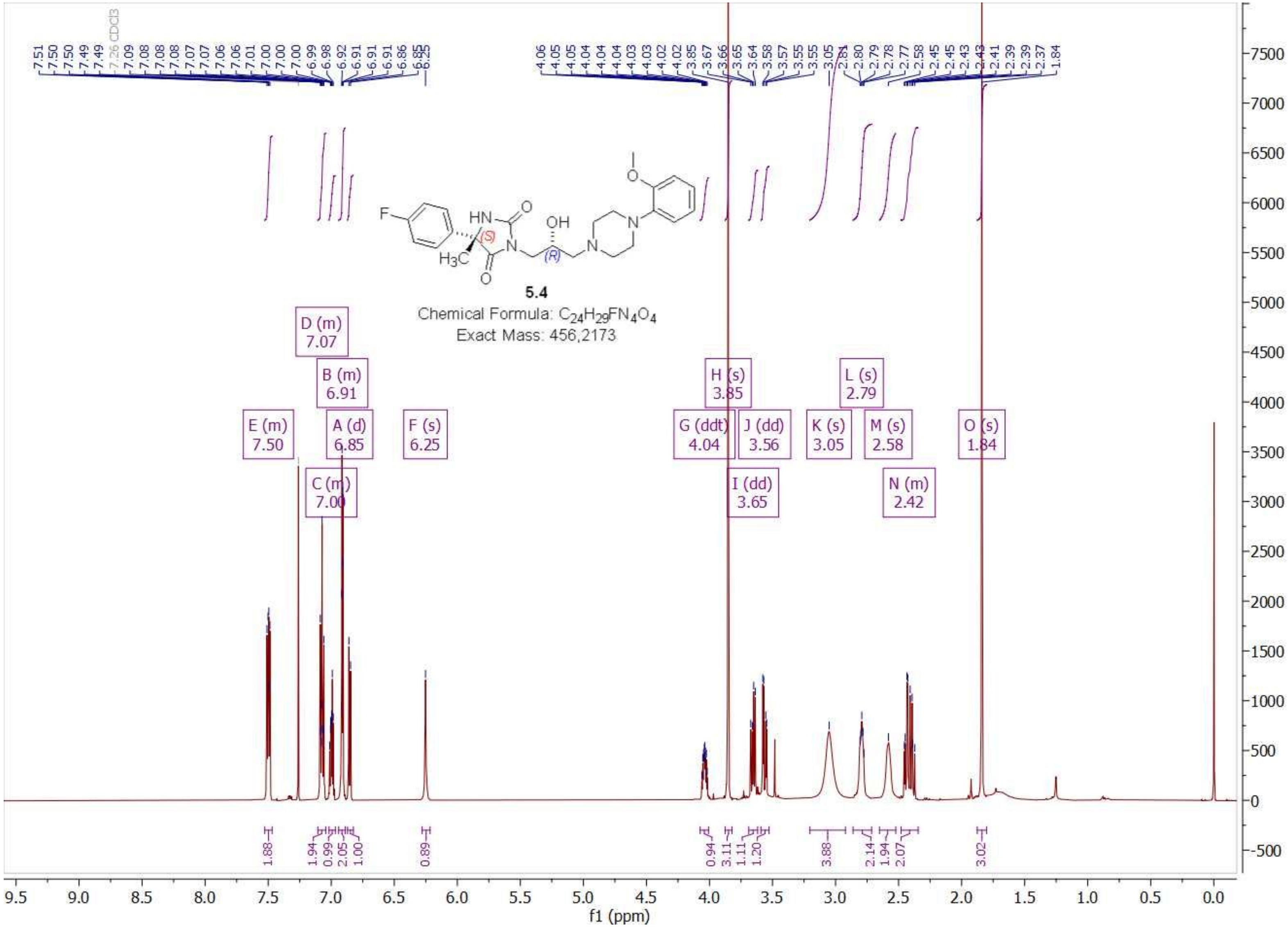

Figure S18. The ^{1}H NMR spectra of pure compound **5.4**

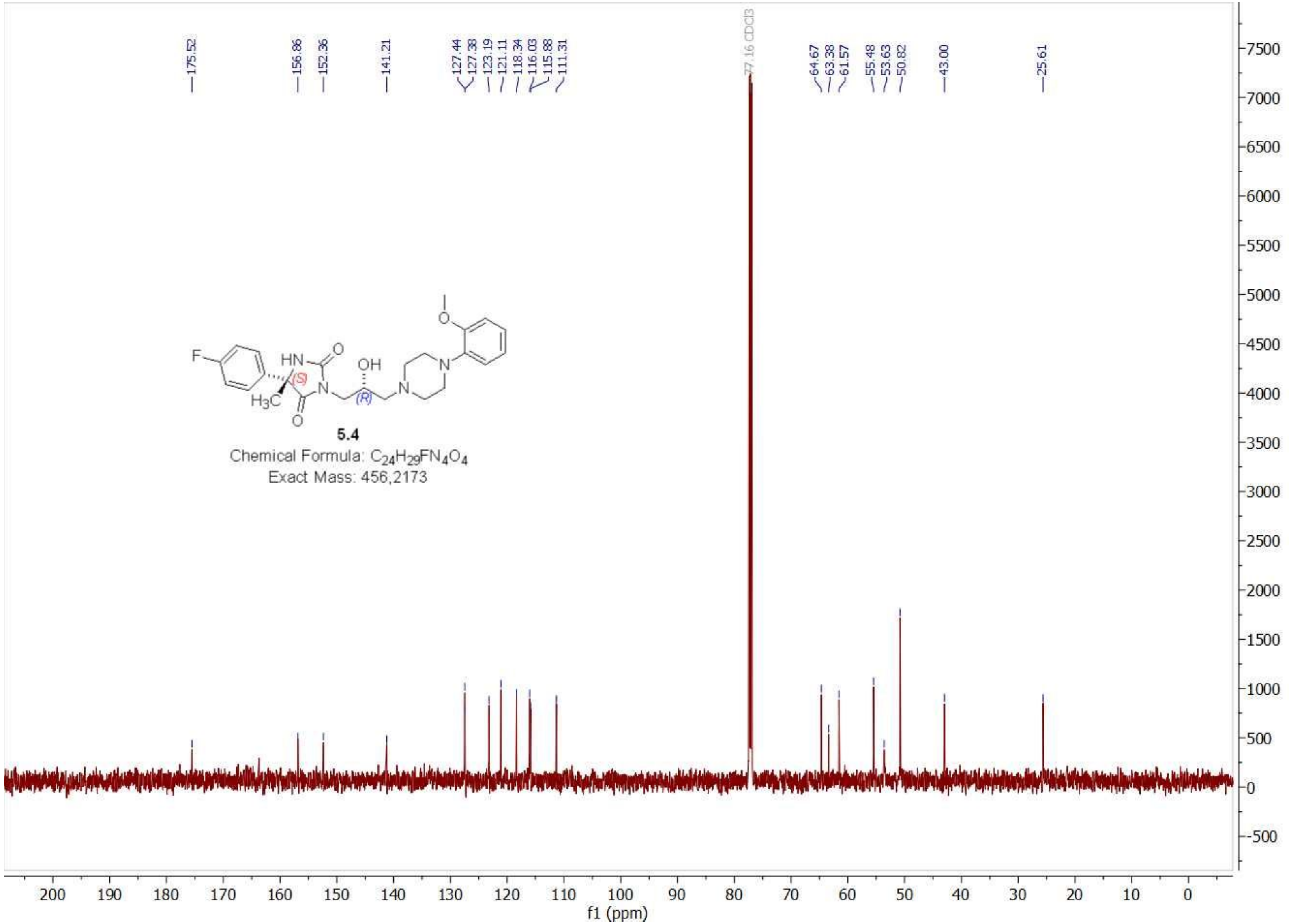

Figure S19. The ^{13}C NMR spectra of pure compound **5.4**

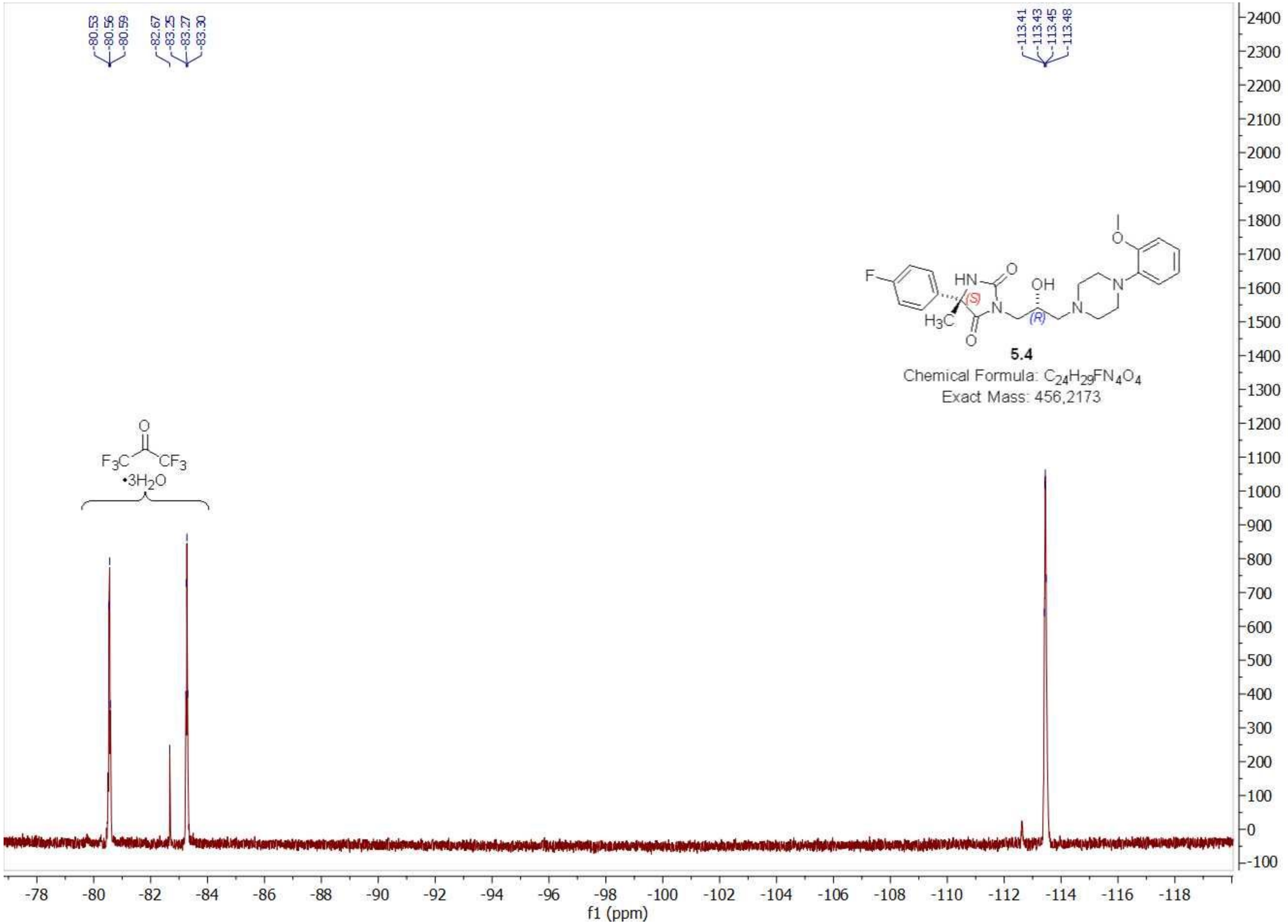

Figure S20. The ^{19}F NMR spectra of pure compound **5.4**

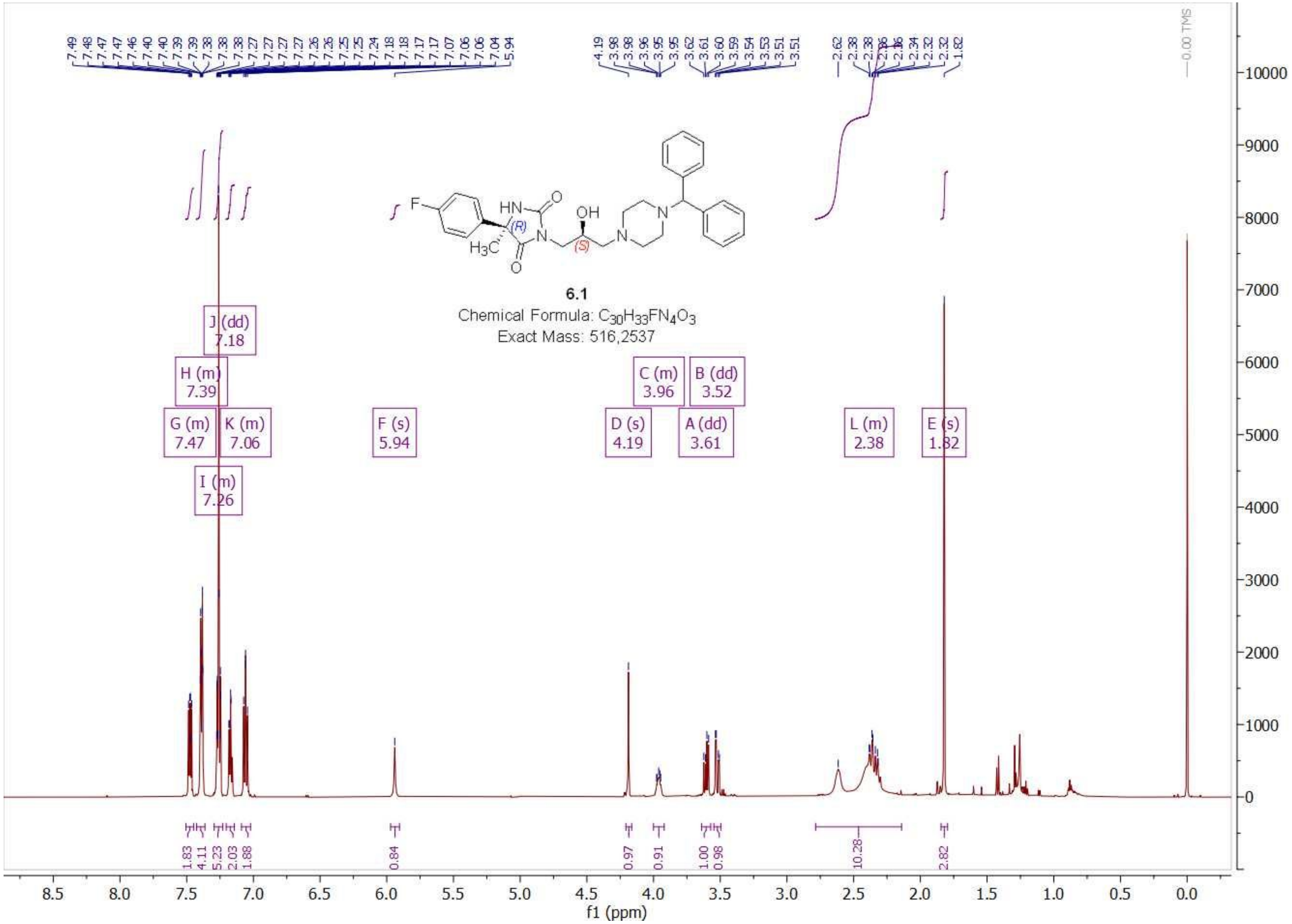

Figure S21. The ^{1}H NMR spectra of pure compound **6.1**

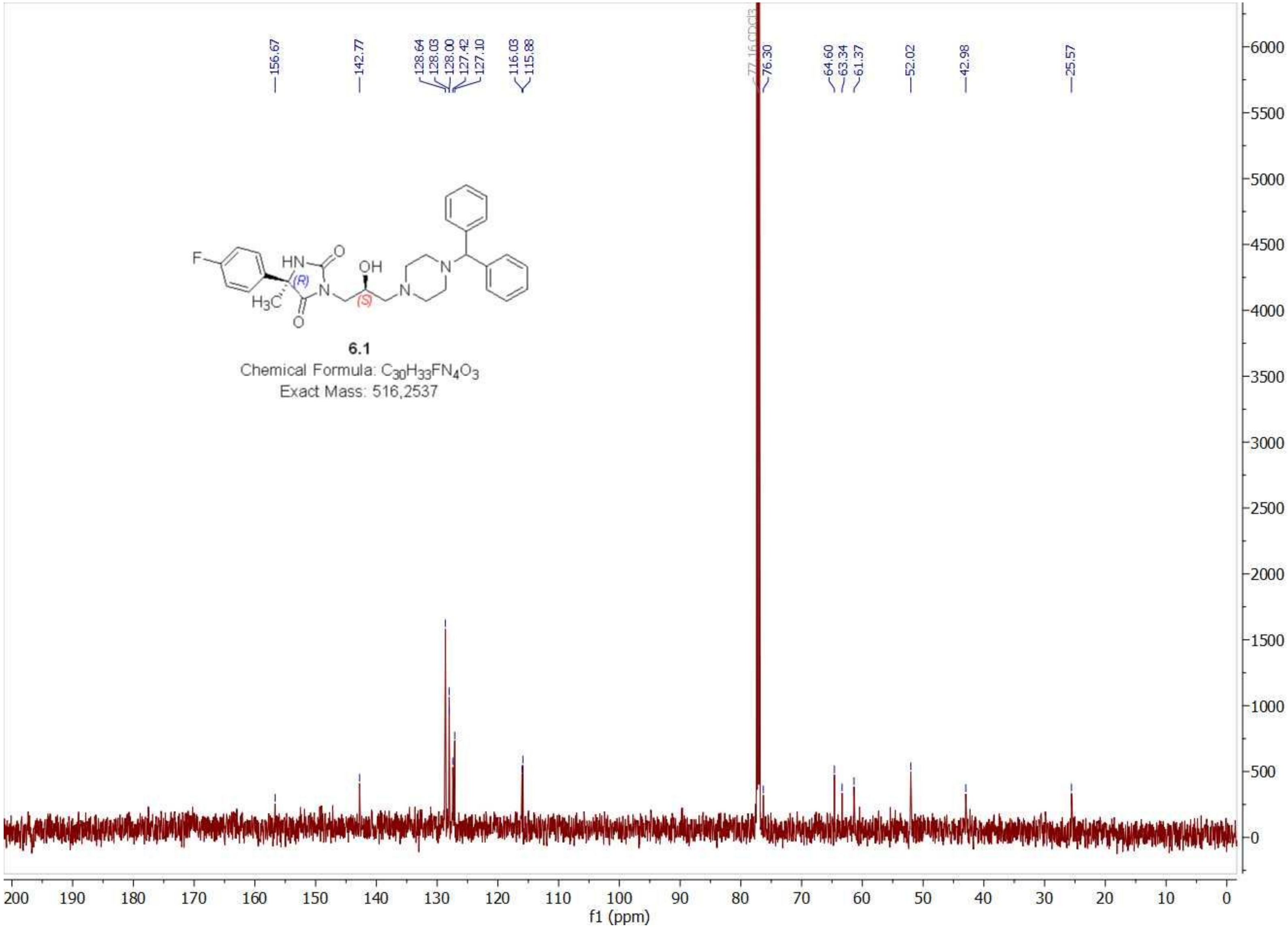

Figure S22. The ^{13}C NMR spectra of pure compound **6.1**

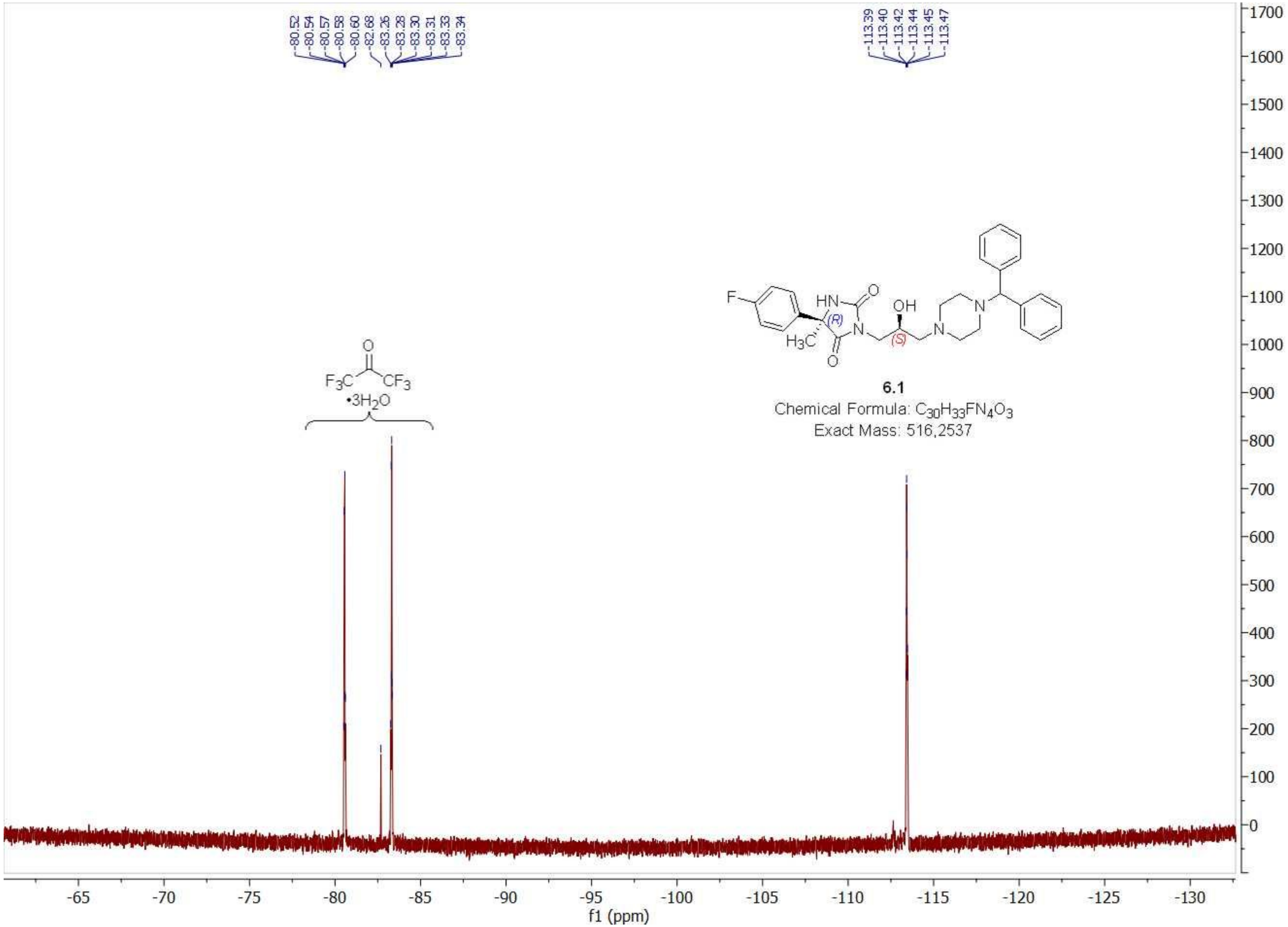

Figure S23. The ^{19}F NMR spectra of pure compound **6.1**

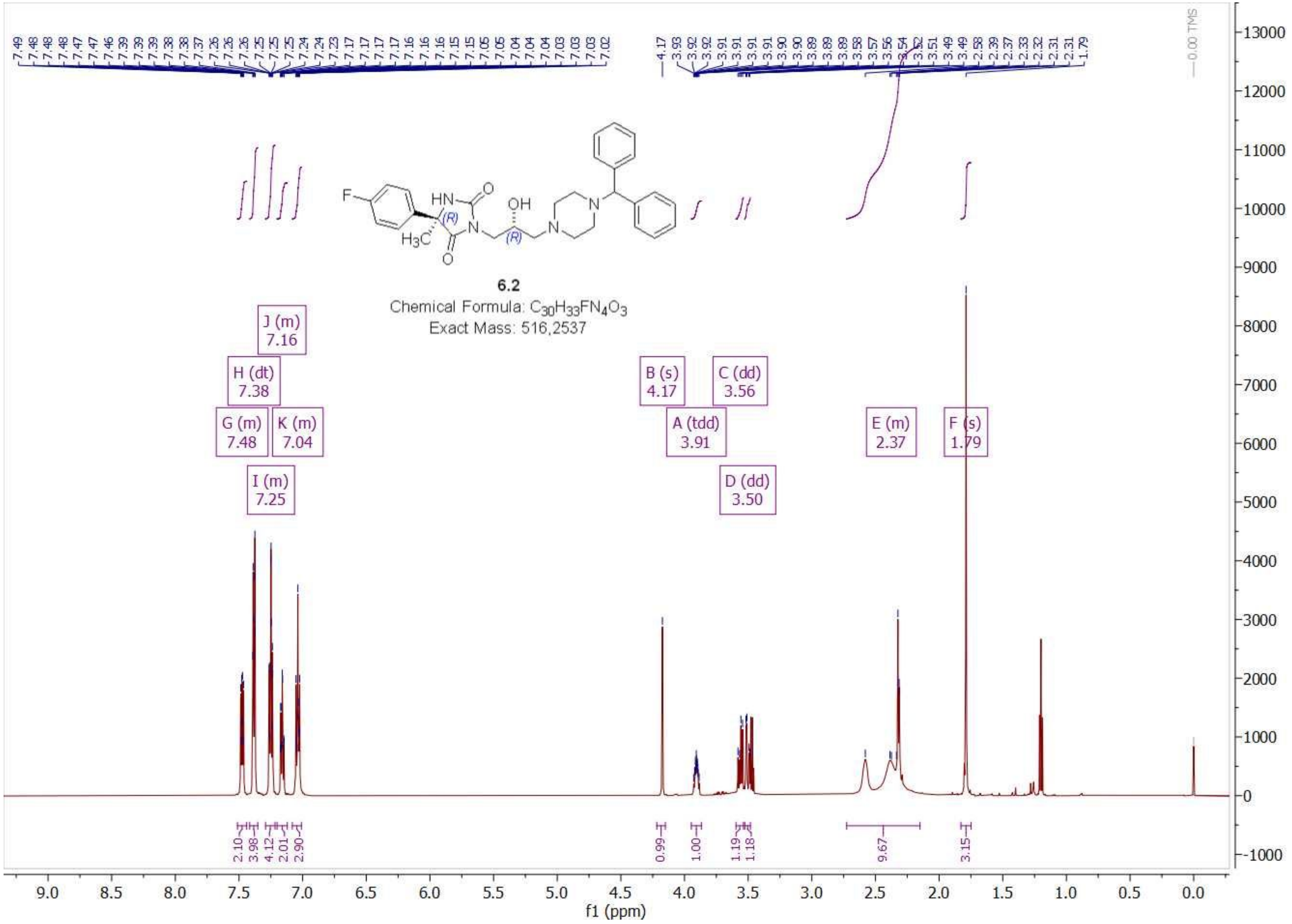

Figure S24. The ^{1}H NMR spectra of pure compound **6.2**

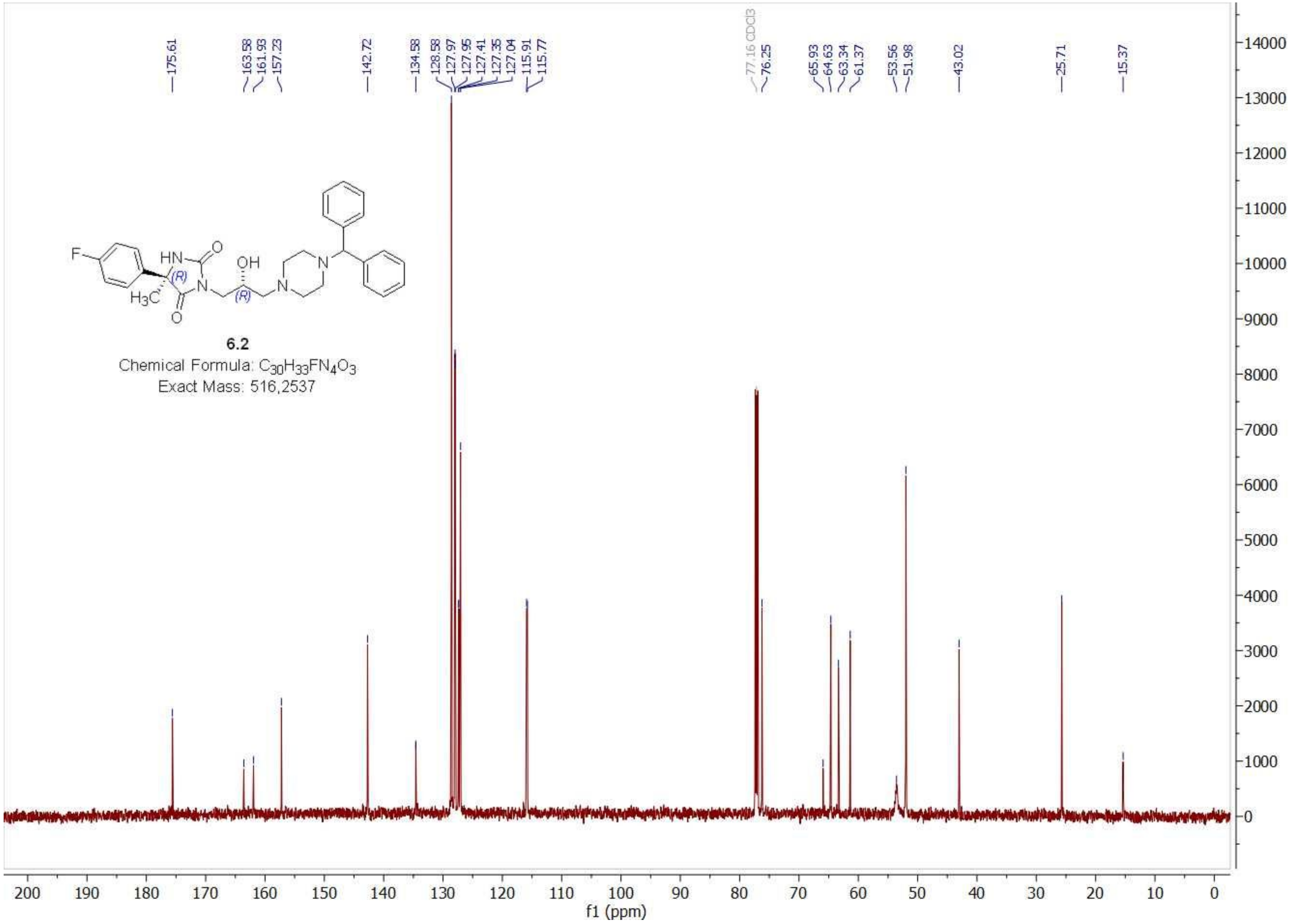

Figure S25. The ^{13}C NMR spectra of pure compound **6.2**

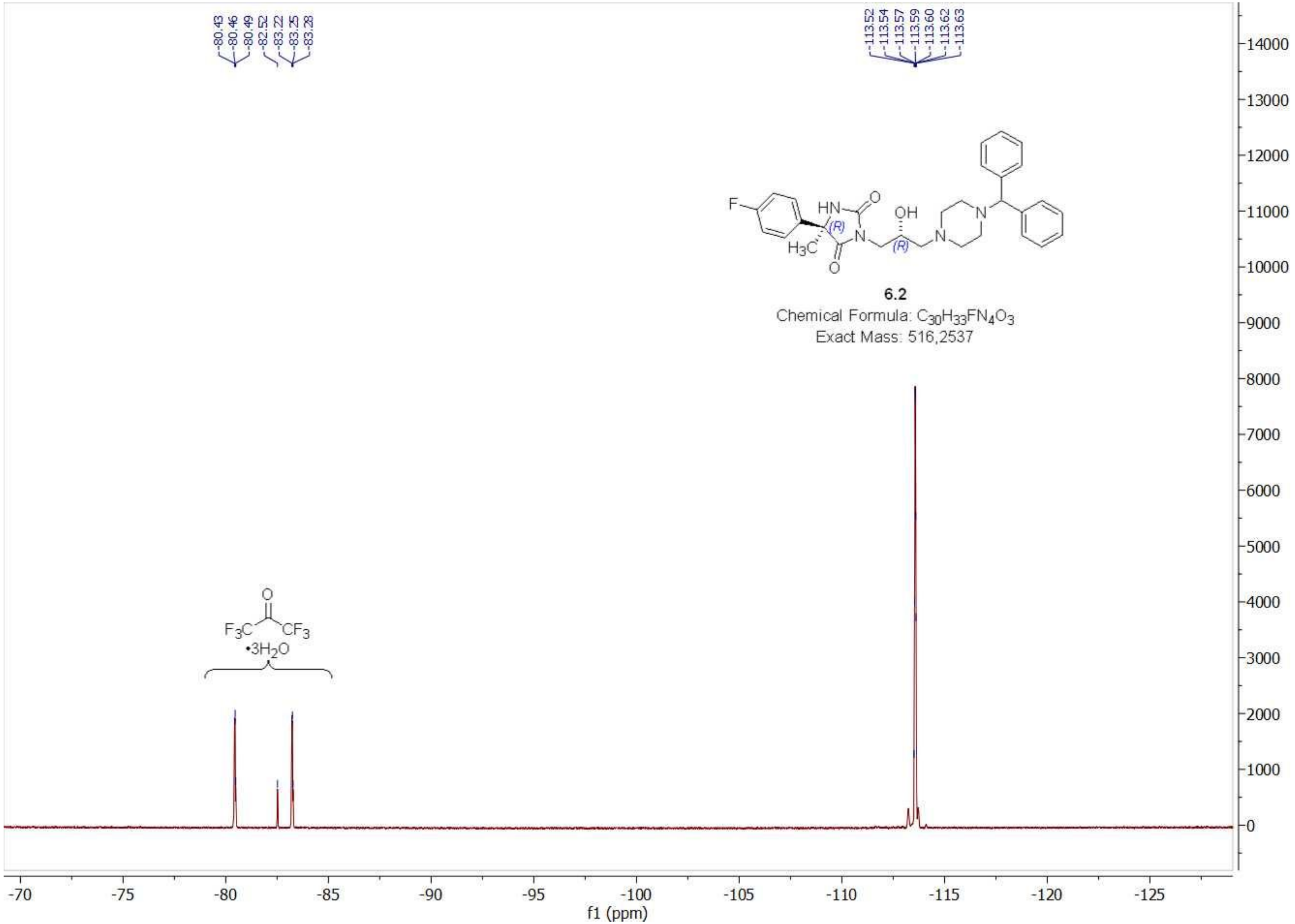

Figure S26. The ^{19}F NMR spectra of pure compound **6.2**

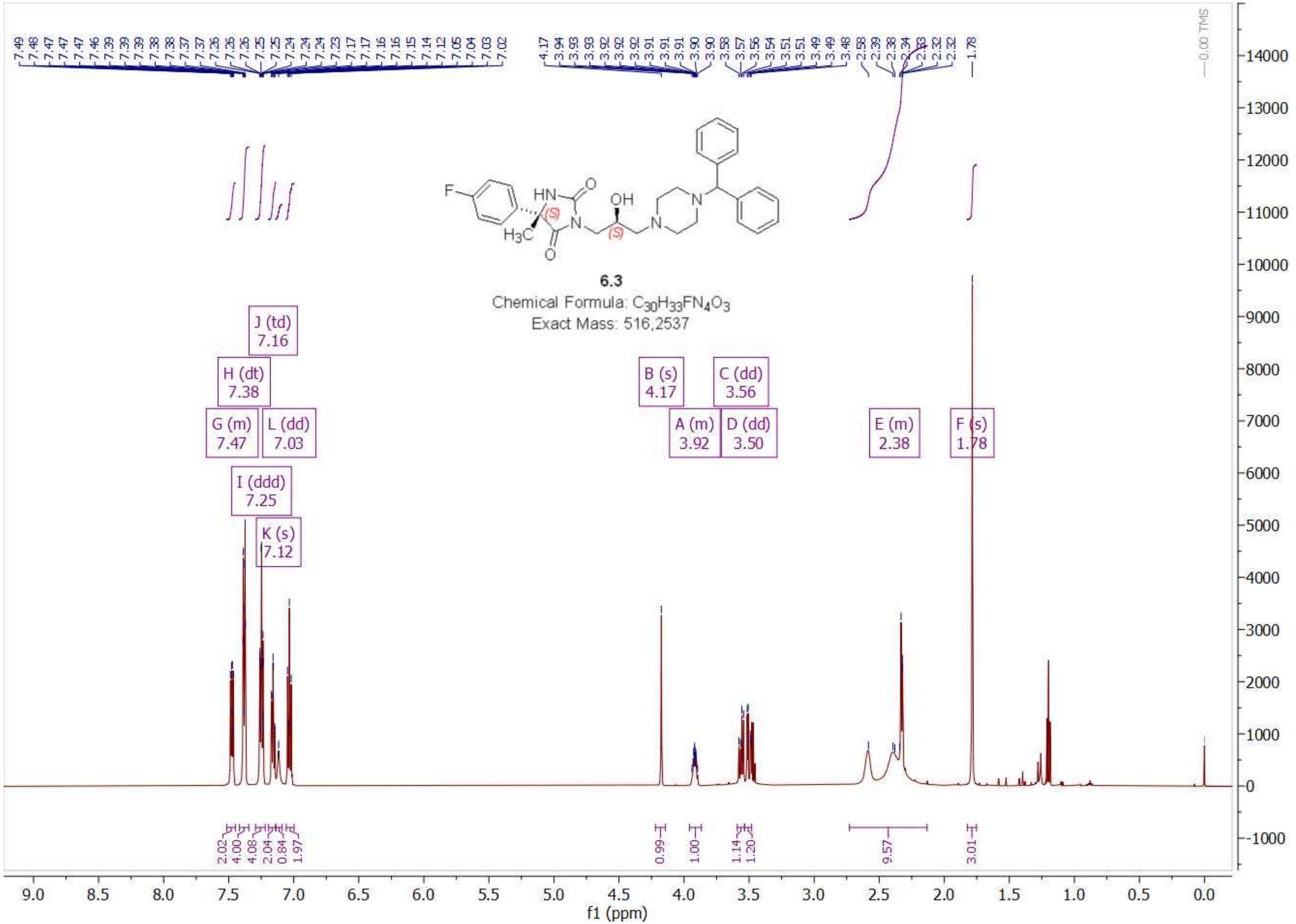

Figure S27. The ^{1}H NMR spectra of pure compound **6.3**

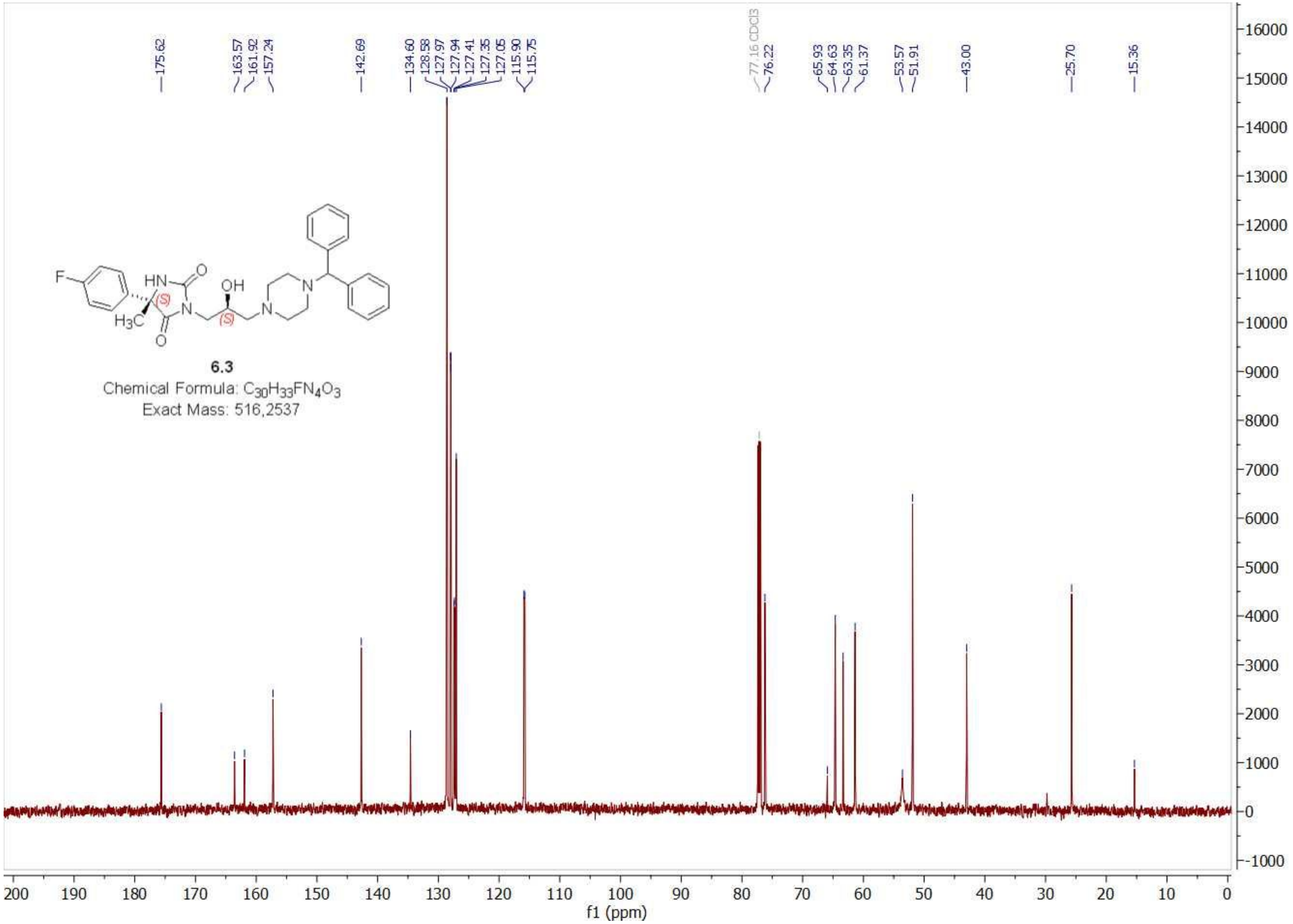

Figure S28. The ^{13}C NMR spectra of pure compound **6.3**

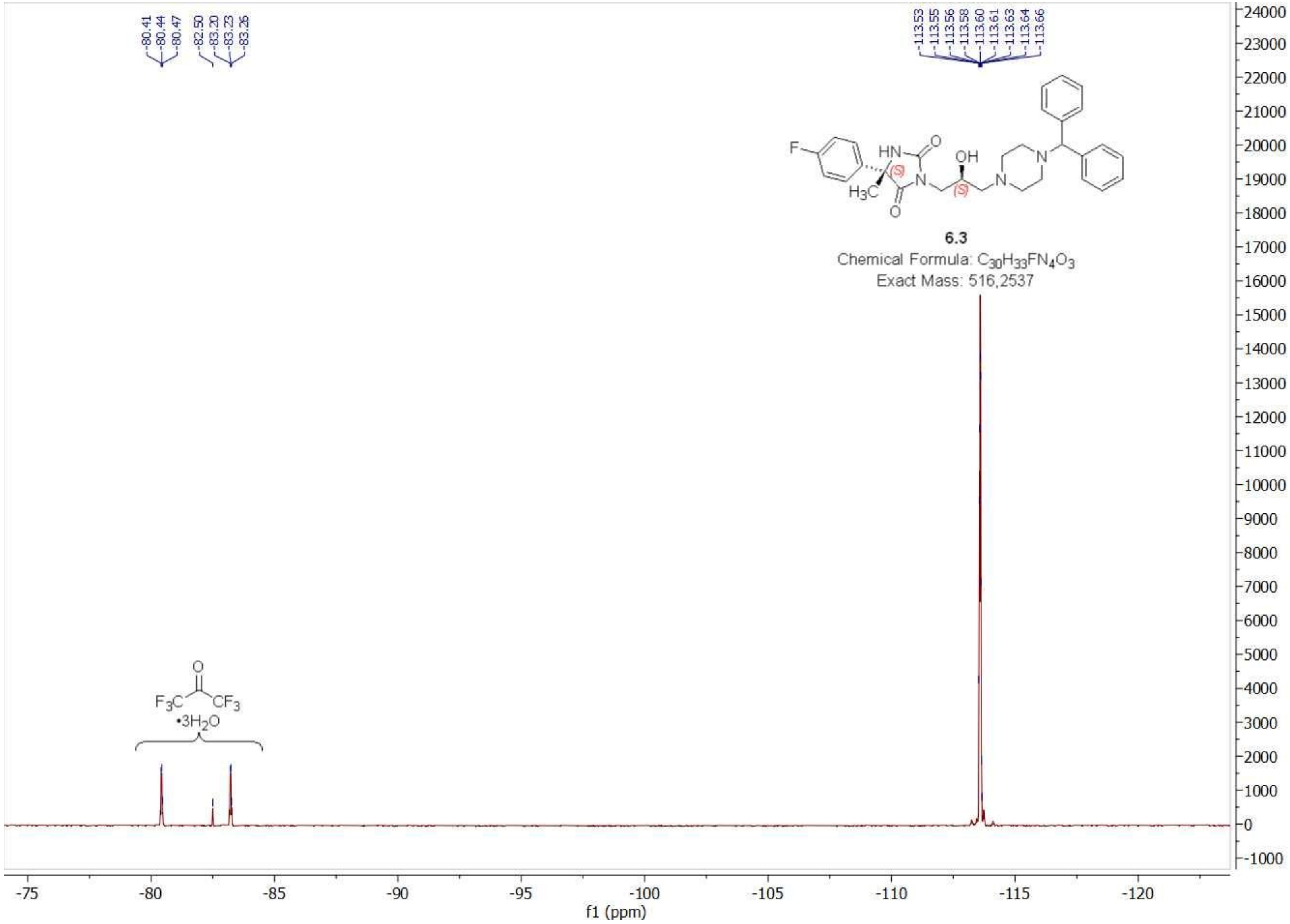

Figure S29. The ^{19}F NMR spectra of pure compound **6.3**

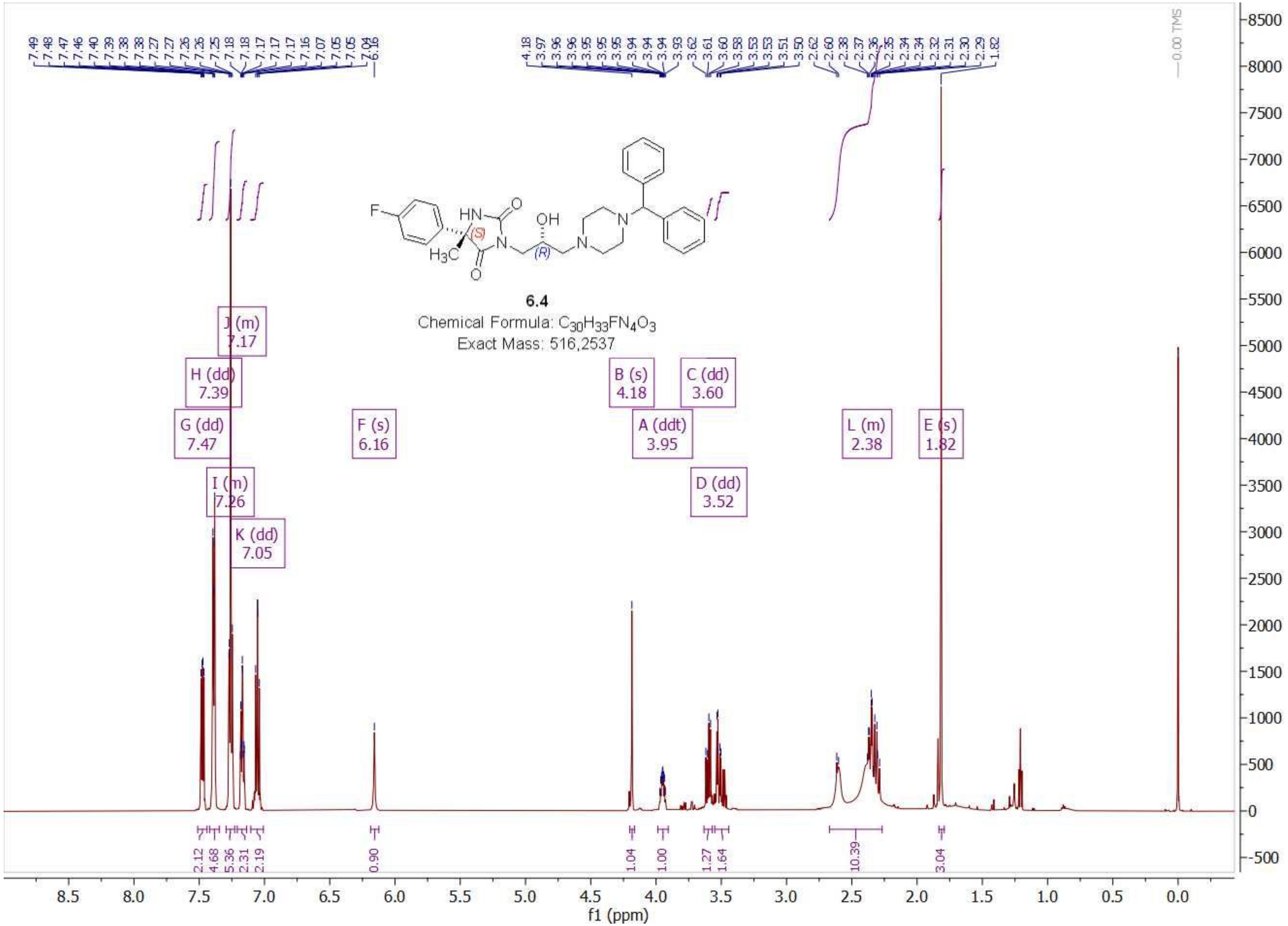

Figure S30. The ^{1}H NMR spectra of pure compound **6.4**

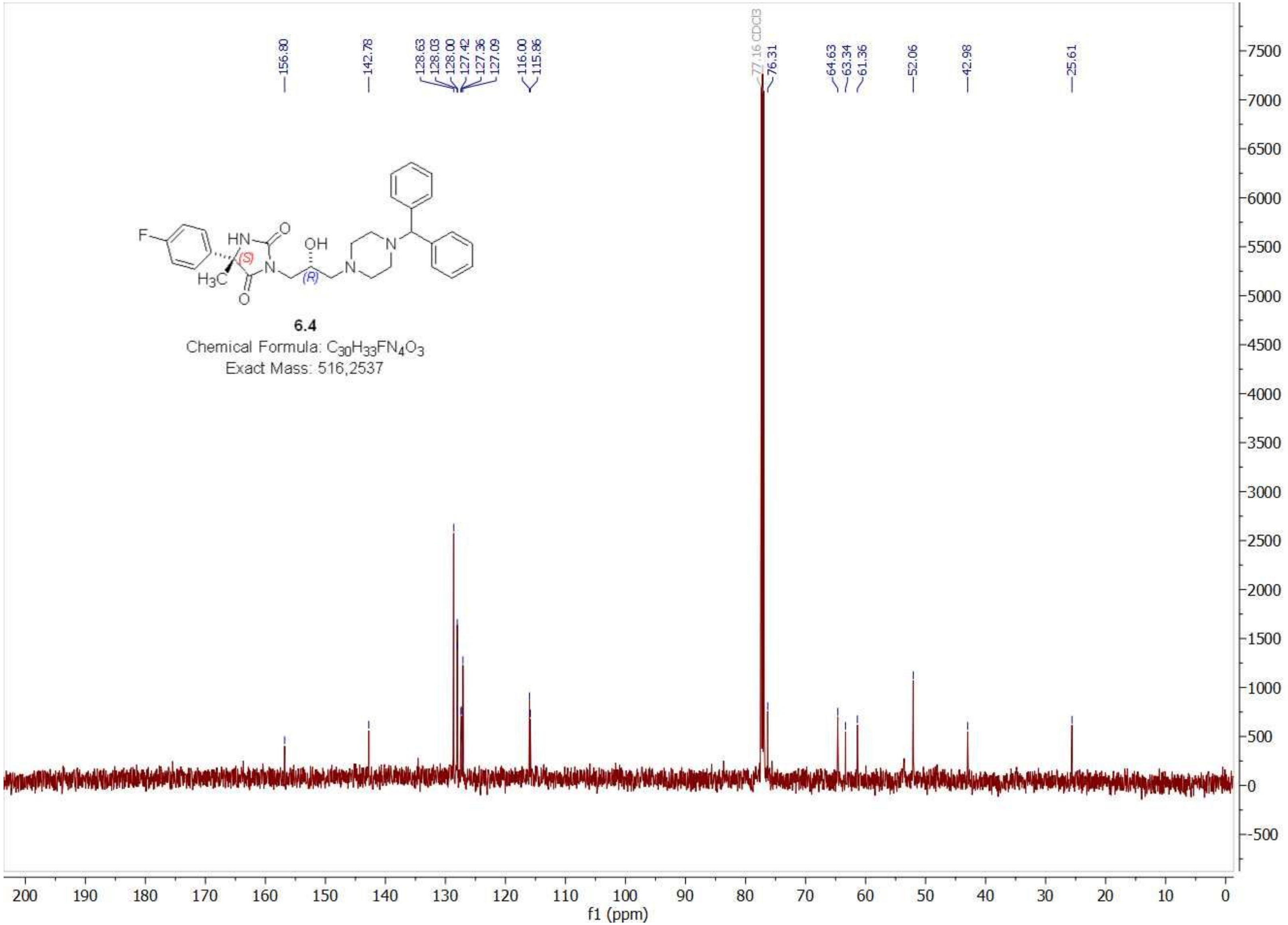

Figure S31. The ^{13}C NMR spectra of pure compound **6.4**

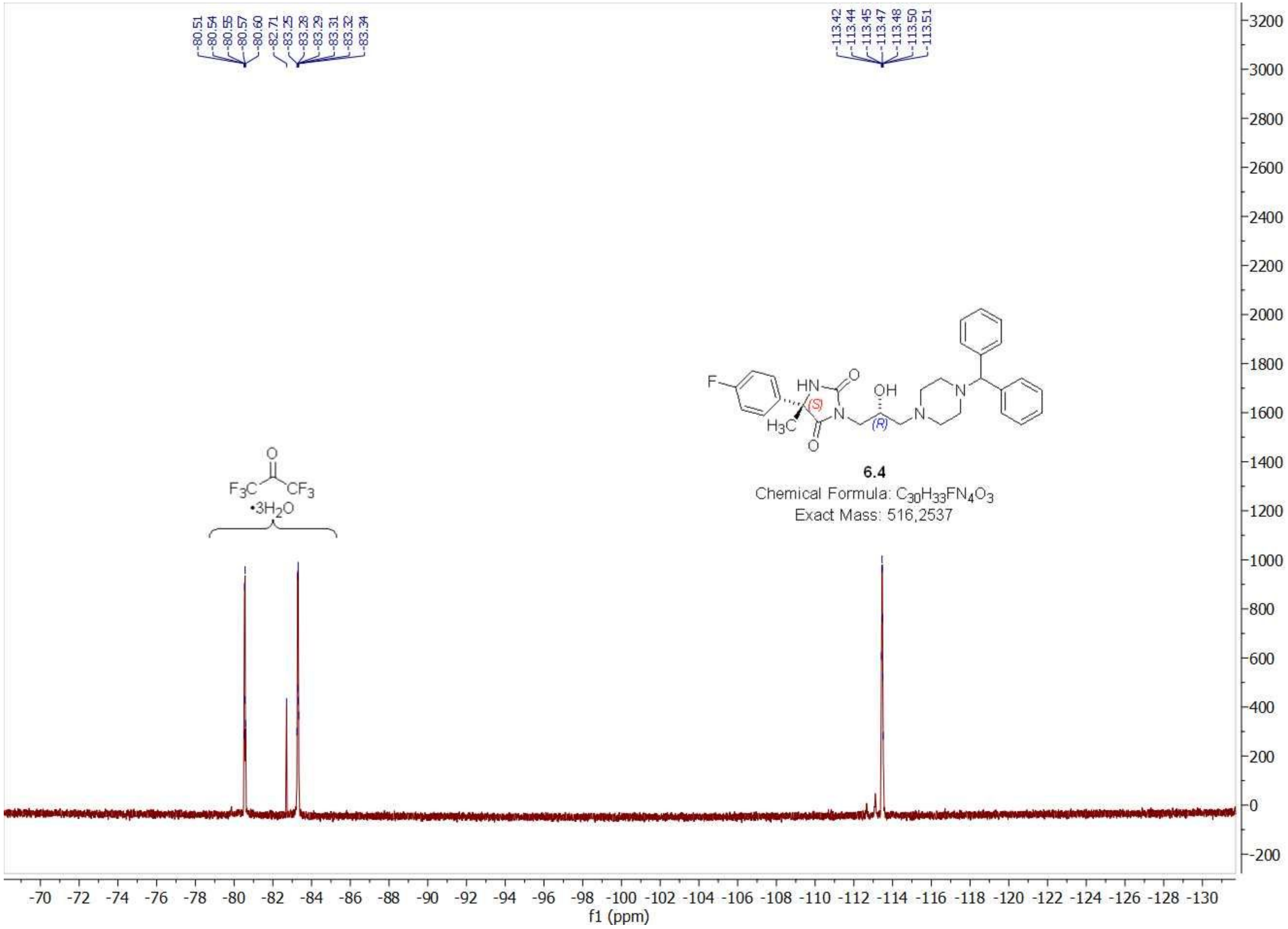

Figure S32. The ^{19}F NMR spectra of pure compound **6.4**

4. The extended NMR studies to elucidate the the differences between expected protons/carbons and observed in spectra

Due to the low solubility of compounds **3.1** and **3.2** in $CDCl_3$, the solvent of choice was MeOD-d_4. However, in protic solvents such as MeOD-d_4, hydantoin protons are exchanged with deuterium from the solvent OD, resulting in the disappearance of their signals on ^{1}HNMR (Figure 1).

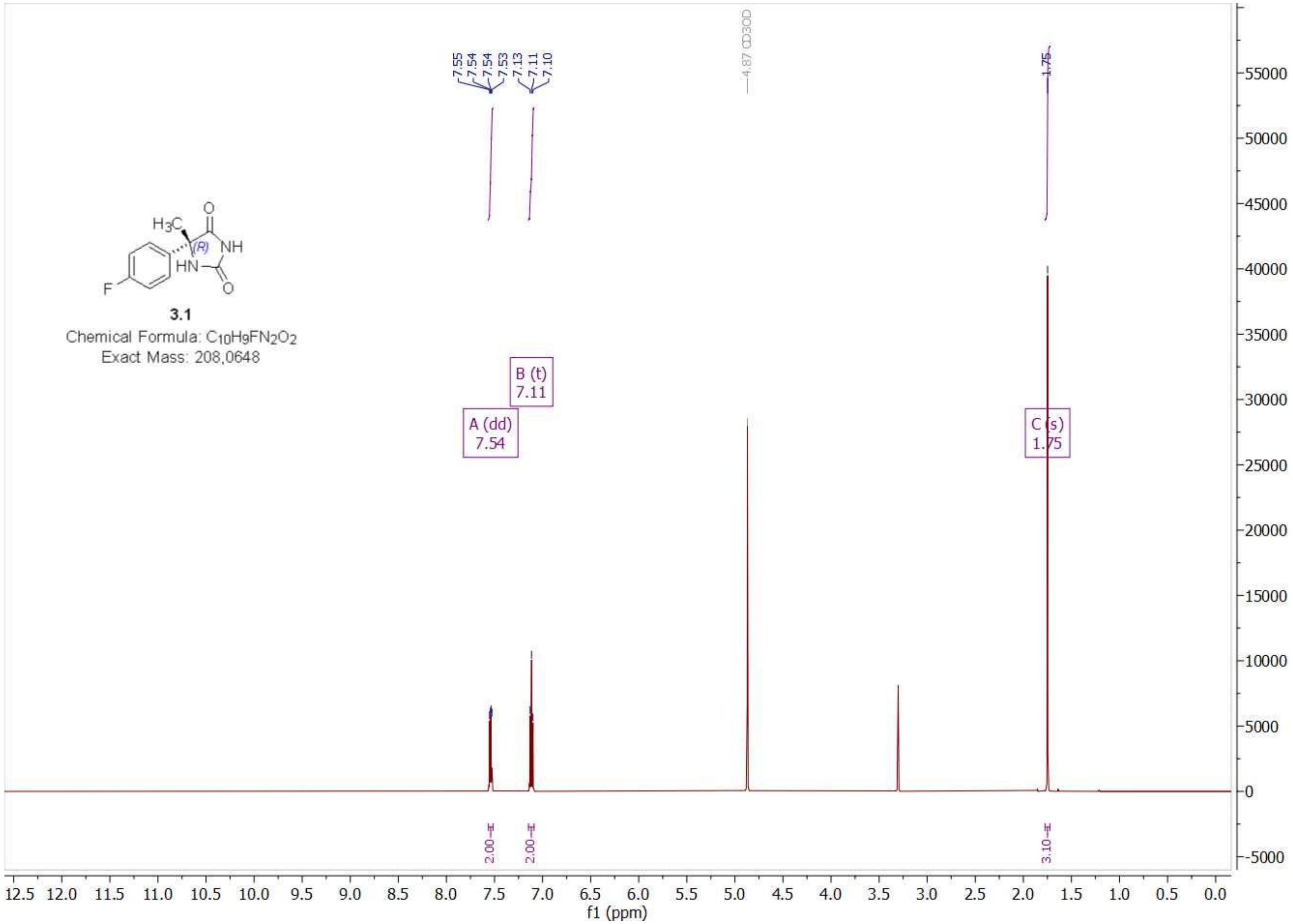

Figure S33. The ^{1}HNMR spectra of compound **3.1** in MeOD-d_4 provided in the manuscript

In the case of polar aprotic solvents such as MeCN-d$_3$ or DMSO-d$_6$, which are not prone to proton/deuterium exchange, the discussed signals are visible in the ^{1}H NMR spectra (Figures 2 and 3).

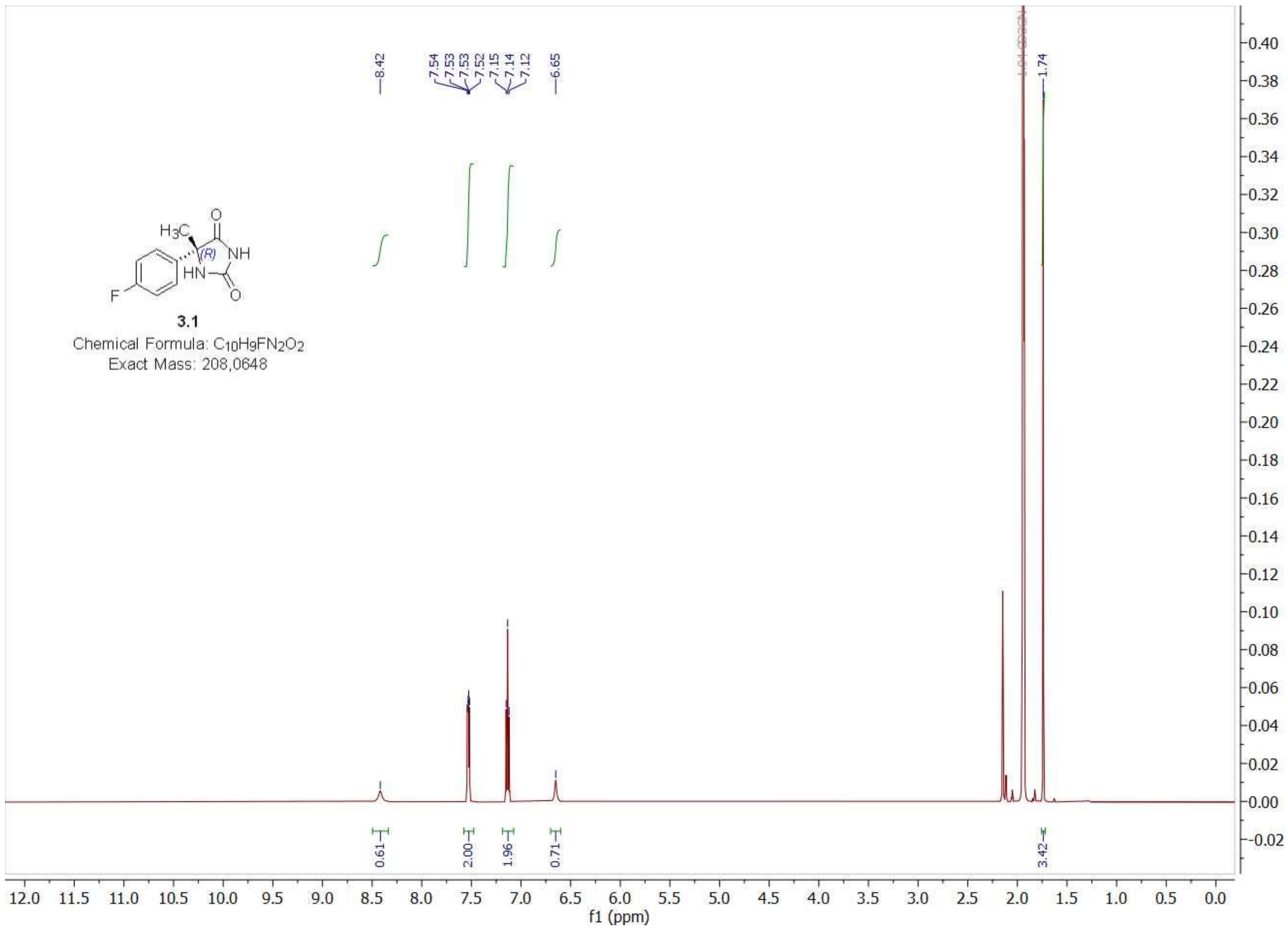

Figure S34. The ^{1}HNMR spectra of compound **3.1** in MeCN-d$_3$

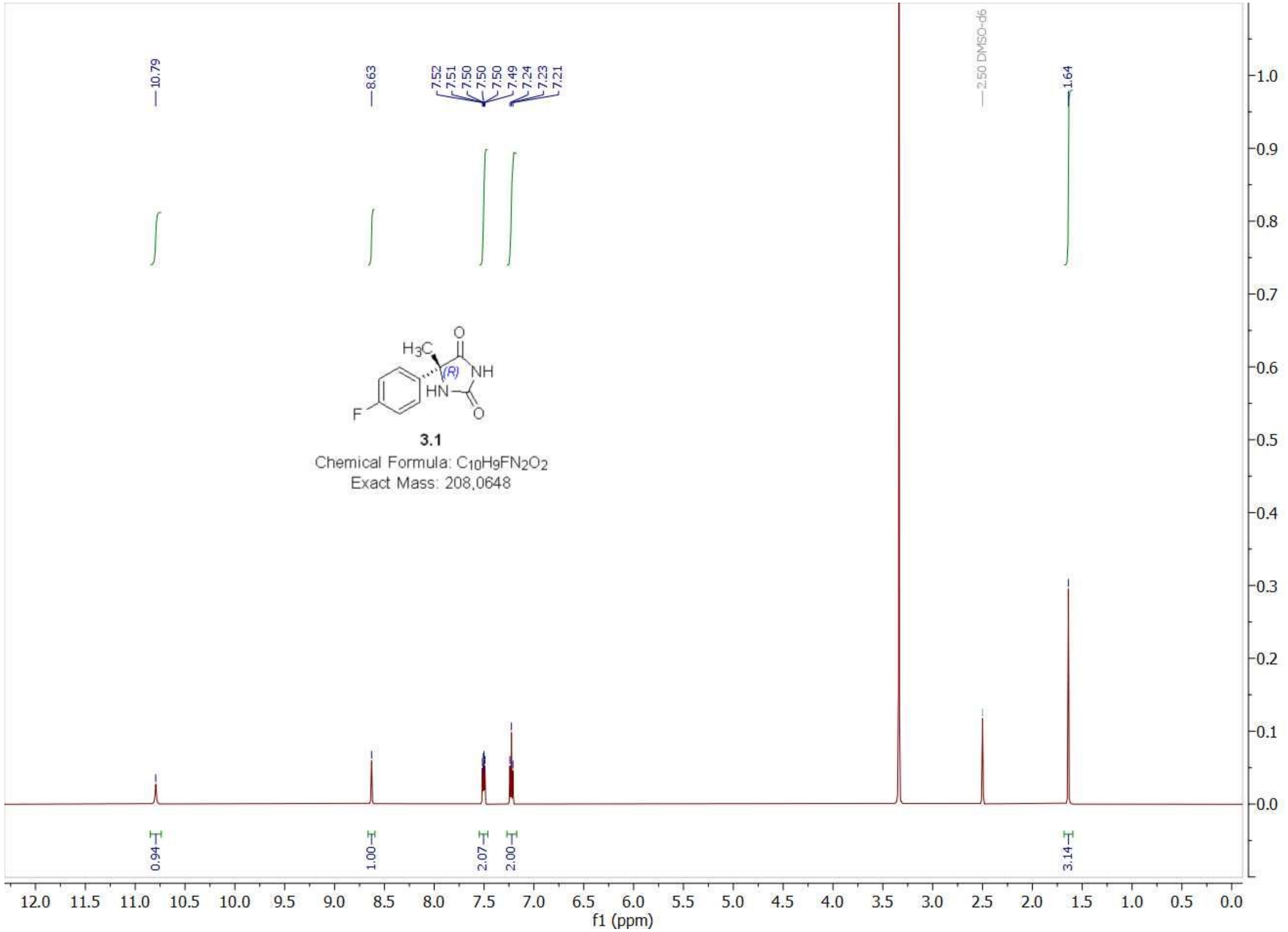

Figure S35. The ^{1}HNMR spectra of compound **3.1** in DMSO-d_6

The ^{1}HNMR signals of the amide (6.65 and 8.63 ppm) and imide (8.42 and 10.79 ppm) protons of compound **3.1** can be easily observed in MeCN-d_3 and DMSO-d_6, respectively (Figures 2 and 3).

The problem of redundant signal in the ^{13}CNMR spectra of compounds **3.1** and **3.2** is related to the presence of a fluorine atom in the structure. It is well known that ^{13}C and ^{19}F (spin = ½, 100% abundance) atoms are effectively coupled in ^{13}C NMR spectra. ^{13}C NMR spectra are usually not fluorine-19 decoupled, so that fluorine-related splitting patterns of carbon signals appear in ^{1}D ^{13}C NMR spectra (Figure 4). This was also the case for the ^{13}C NMR spectra provided in the manuscript. The common values of the coupling constant (J) observed for carbon signals as a function of the bond distance are as follows $^{1}J_{CF}$ = 245 Hz, $^{2}J_{CF}$ = 21 Hz, $^{3}J_{CF}$ = 8 Hz, $^{4}J_{CF}$ = 3 Hz). To confirm this, we performed an additional ^{13}CNMR experiment with fluorine-carbon decoupling (Figure 5).

Comparing the standard ^{13}CNMR (Figure 4) and the decoupled ^{13}CNMR (Figure 5), it can be seen that the signals at 164.8 and 163.2 ppm (Figure 4) merge into a signal at 164.0 ppm (Figure 5) with increased intensity (Figure 6). The same effect can be observed for the signals 136.9, 128.6 and 116.4 ppm. If we calculate the J coupling constants in Hz from the ppm shifts of the signals, we find 244 Hz for 164 ppm, 38 Hz for 116 ppm, 8 Hz for 128 ppm and 3 Hz for 136 ppm, which is in excellent agreement with theory.

We also performed the ^{1}HNMR spectra with hydrogen-fluorine decoupling (Figure 7). It can also be seen that the signal structures of 8.89 and 8.43 ppm simplify and become doublets instead of more complex multiplets.

The same effects are observed for all the compounds presented in the manuscript, since they all have at least one amide proton and a single fluorine atom attached in the para position of the benzene ring in the hydantoin moiety.

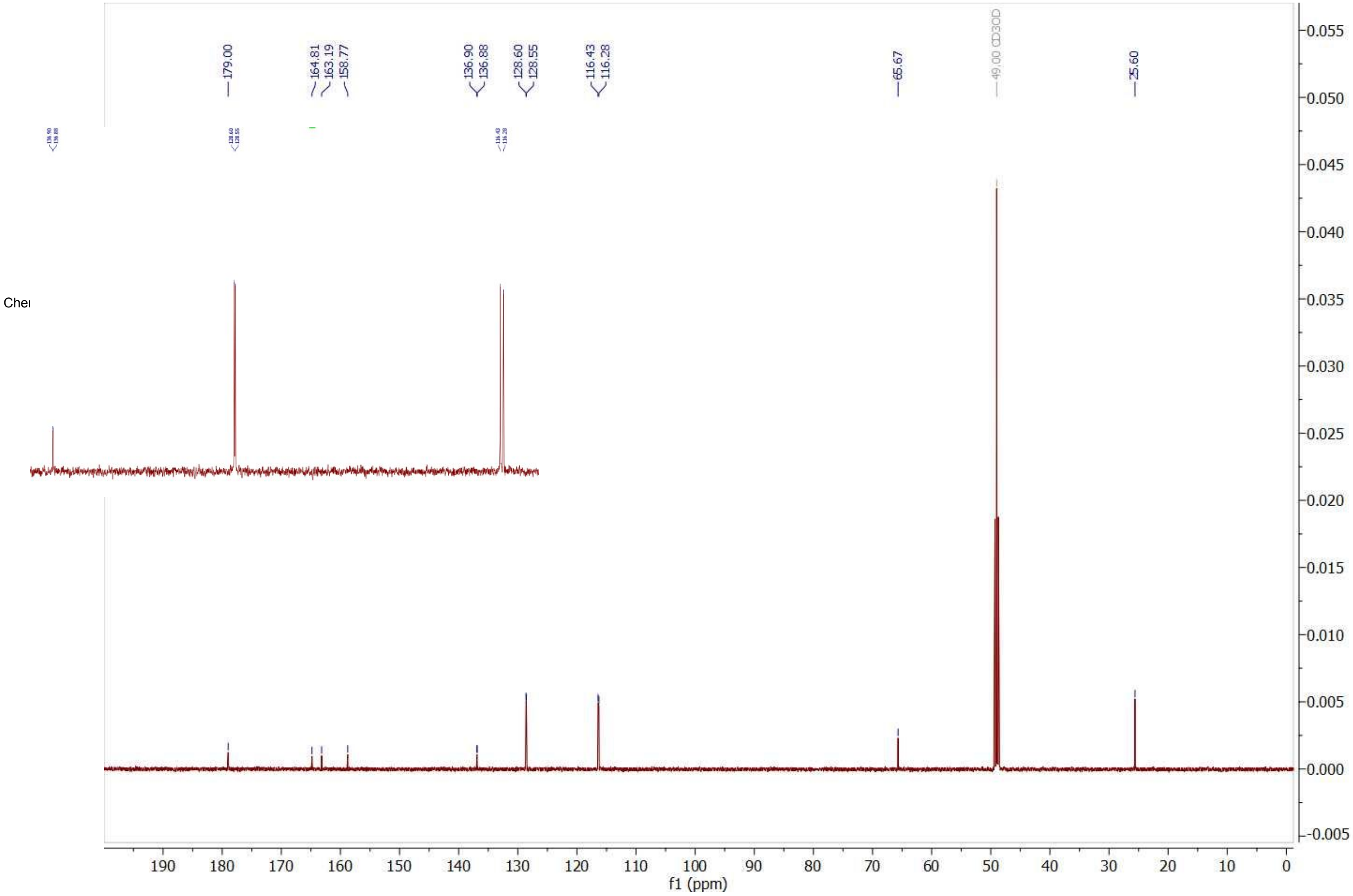

Figure S36. The ^{13}CNMR spectra of compound **3.1** *without* carbon-fluor decoupling.

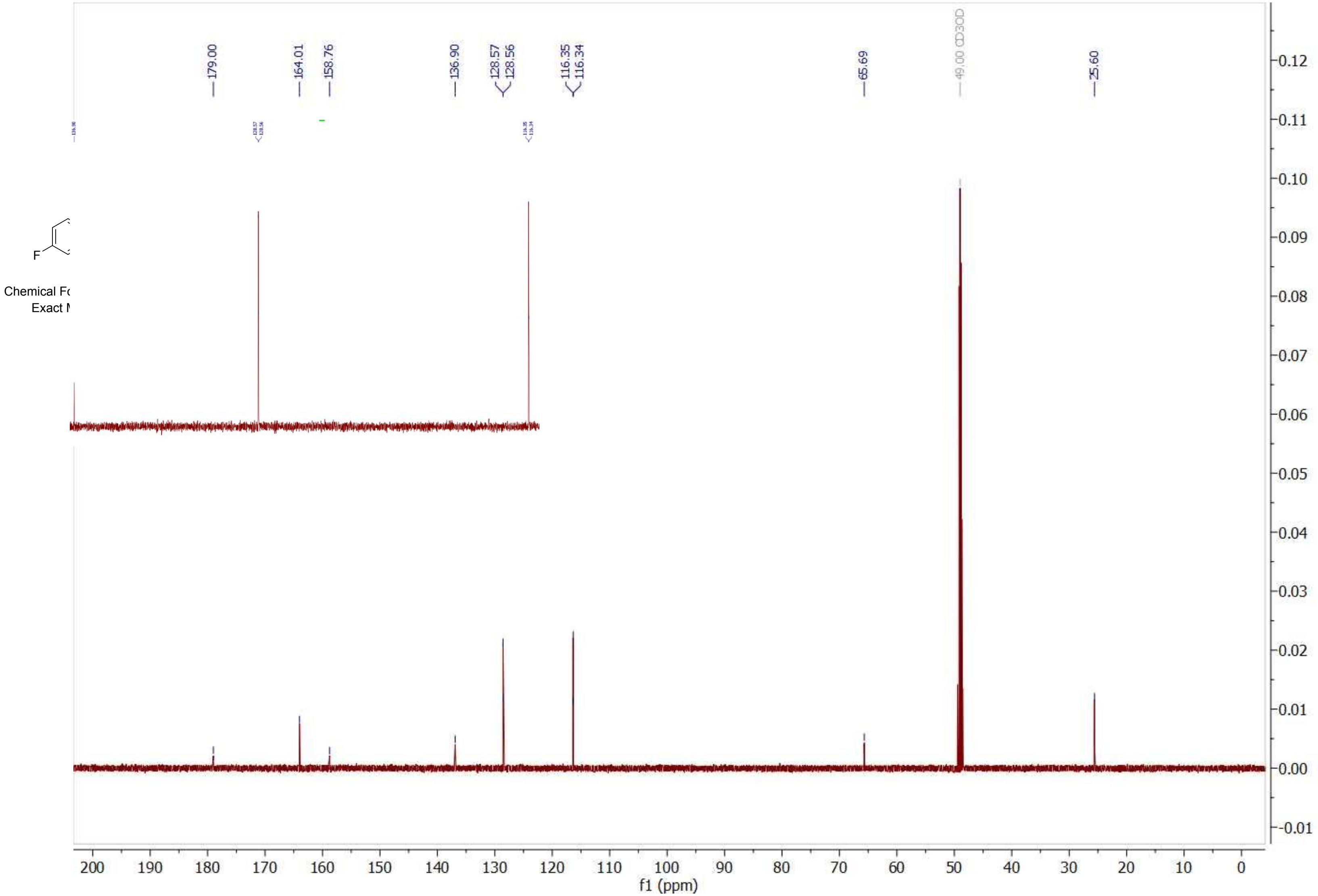

Figure S37. The ^{13}CNMR spectra of compound **3.1** *with* carbon-fluor decoupling.

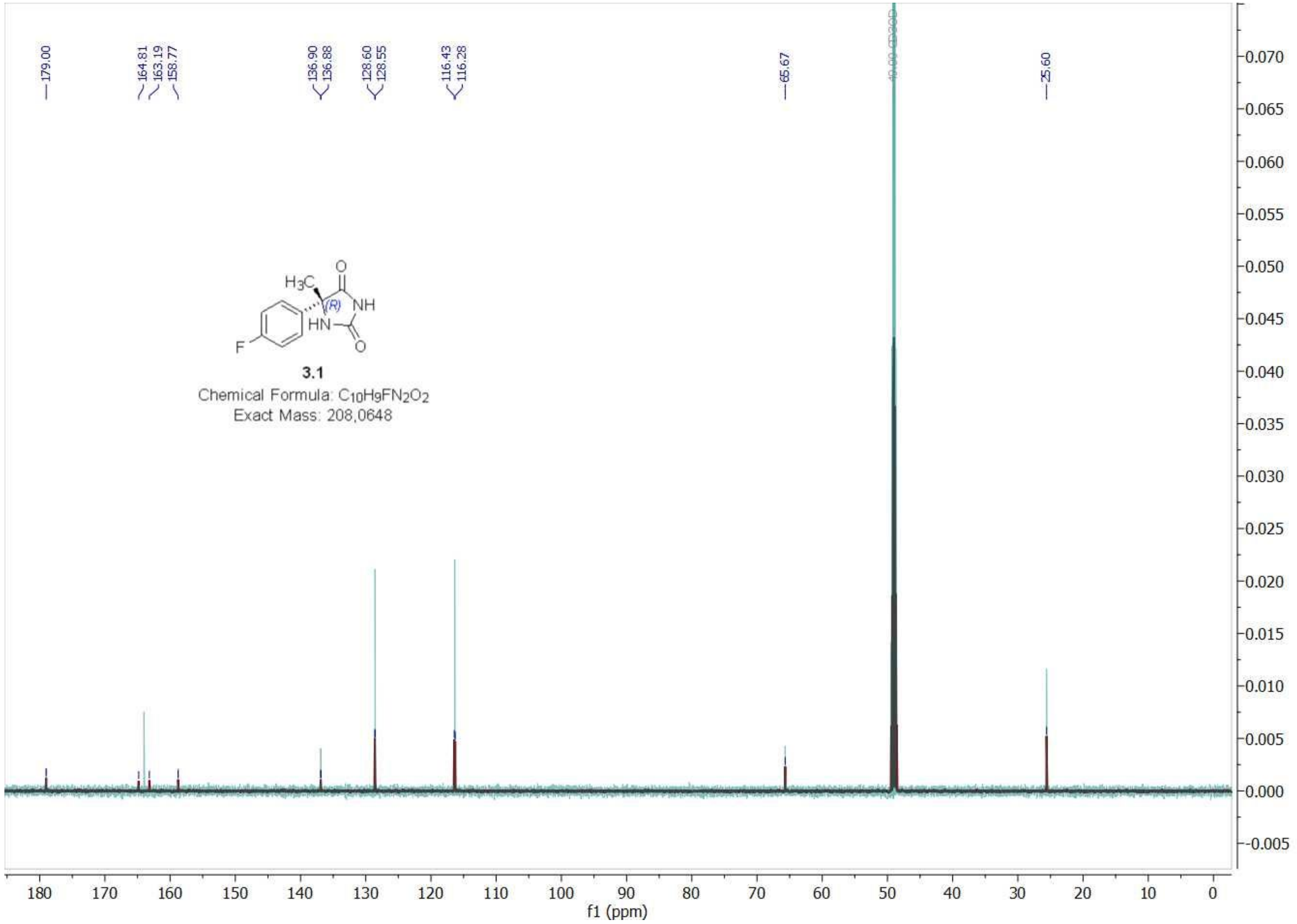

Figure S38. Stacking of both ^{13}CNMR spectres of compound **3.1** with/without carbon-fluor decoupling.

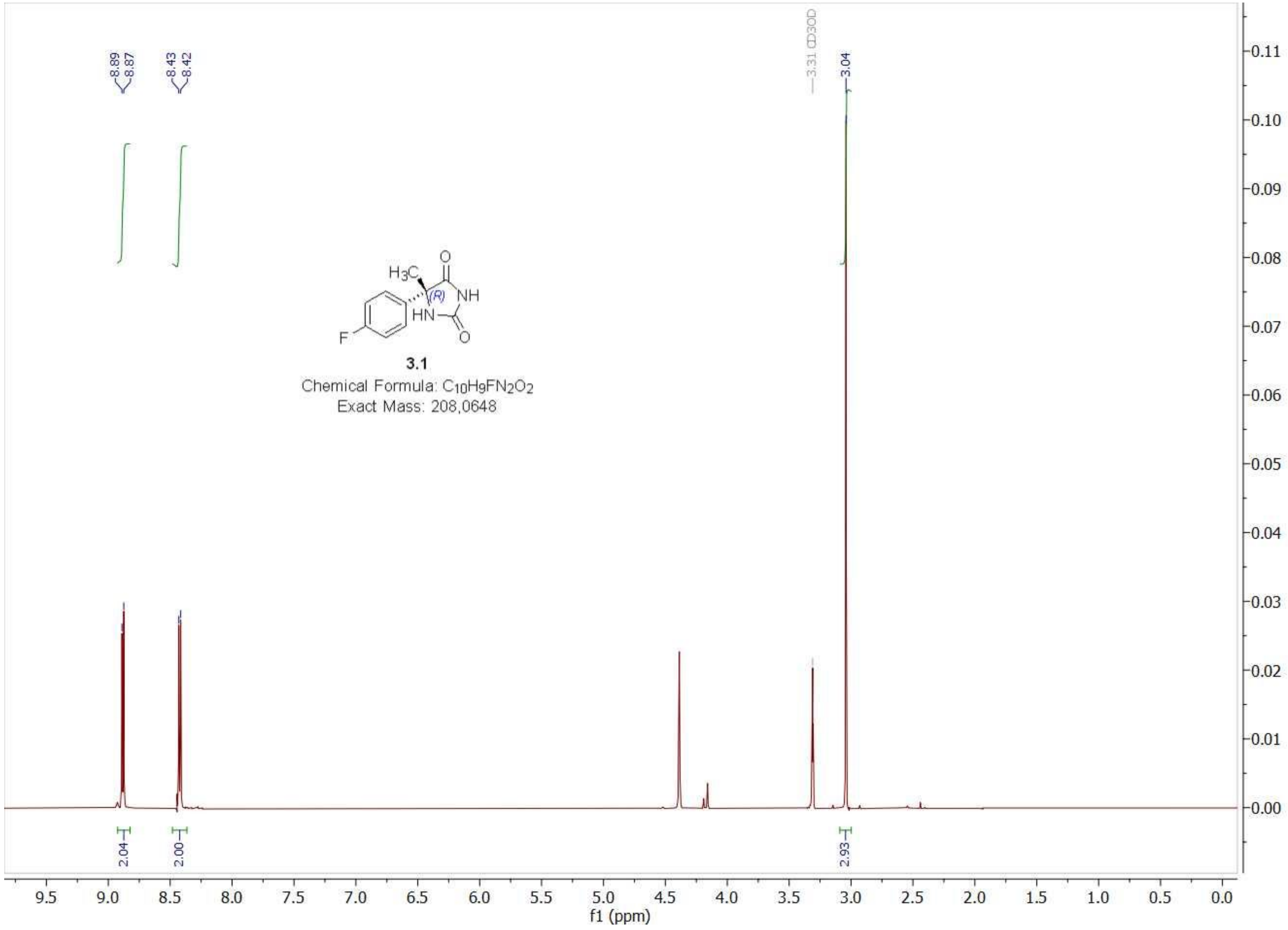

Figure S39. The ^{1}HNMR spectra of compound **3.1** *with* hydrogen-fluor decoupling.

5. HPLC data for intermediate and final compounds

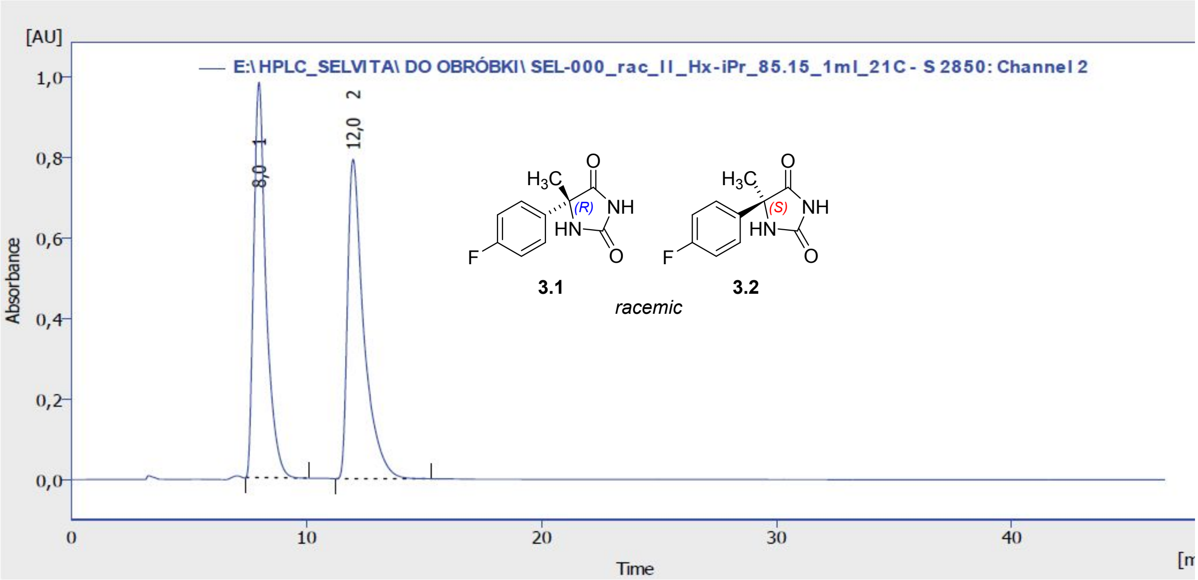

Result Table (Uncal - E:\HPLC_SELVITA\DO OBRÓBKI\SEL-000_rac_II_Hx-iPr_85.15_1ml_21C - S 2850: Cha

	Reten. Time [min]	Area [mAU.s]	Height [mAU]	Area [%]	Height [%]	W05 [min]	PDA Peak Purity	Compound Name
1	7,983	35740,026	981,473	48,4	55,3	0,55	910	
2	11,983	38179,169	793,308	51,6	44,7	0,68	919	
	Total	73919,195	1774,782	100,0	100,0			

Figure S40. The HPLC analysis for racemic mixture of **3.1** and **3.2**

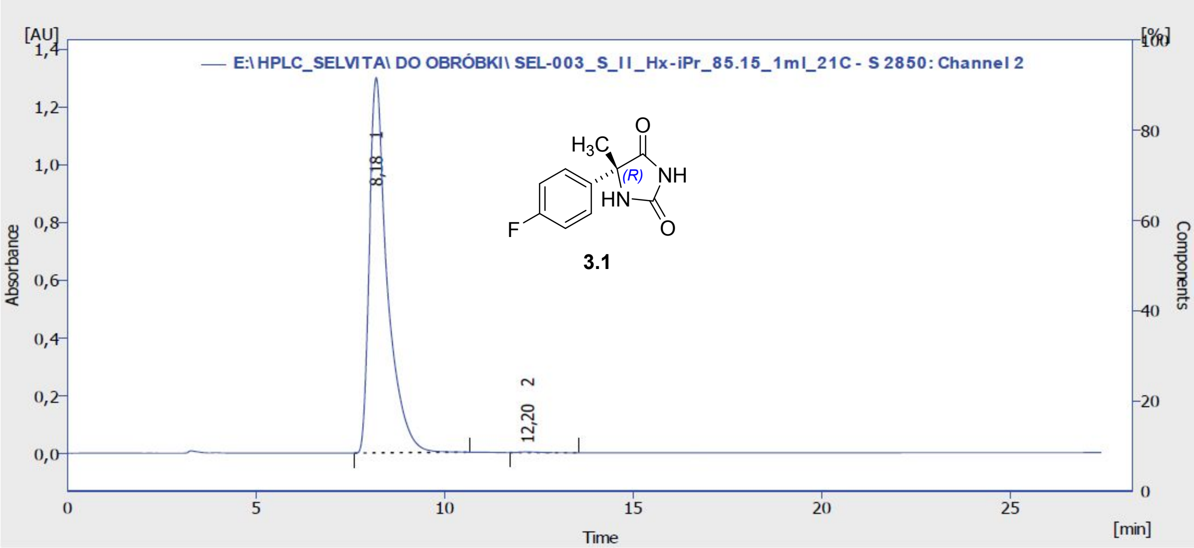

Result Table (Uncal - E:\HPLC_SELVITA\DO OBRÓBKI\SEL-003_S_II_Hx-iPr_85.15_1ml_21C - S 2850: Char

	Reten. Time [min]	Area [mAU.s]	Height [mAU]	Area [%]	Height [%]	W05 [min]	PDA Peak Purity	Compound Name
1	8,183	42917,452	1300,235	99,8	99,8	0,47	859	
2	12,200	86,527	2,426	0,2	0,2	0,52	998	
	Total	43003,979	1302,661	100,0	100,0			

Figure S41. The HPLC analysis for optically pure **3.1**

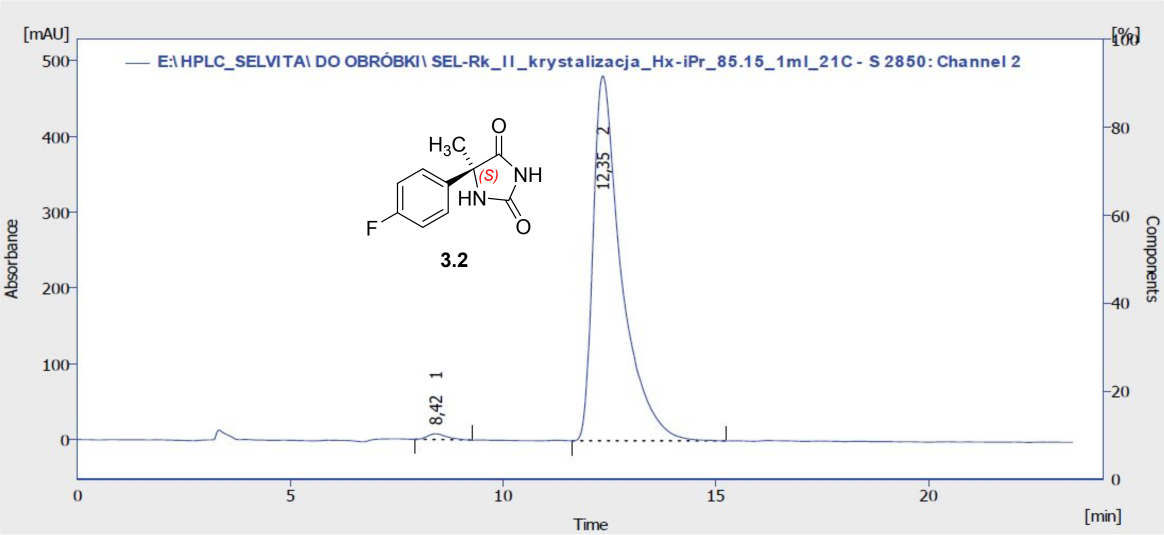

Result Table (Uncal - E:\HPLC_SELVITA\DO OBRÓBKI\SEL-Rk_II_krystalizacja_Hx-iPr_85.15_1ml_21C - S 2850:

	Reten. Time [min]	Area [mAU.s]	Height [mAU]	Area [%]	Height [%]	W05 [min]	PDA Peak Purity	Compound Name
1	8,417	248,737	7,661	1,1	1,6	0,50	926	
2	12,350	22277,845	481,756	98,9	98,4	0,67	850	
	Total	22526,582	489,417	100,0	100,0			

Figure S42. The HPLC analysis for optically pure **3.2**

Object name: Time table - 100% C, 0.8 mL, 254 - Created: 19-02-2002, 02:13
Path:

	Time min	Flow ml/min	Valve %A	Valve %B	Valve %C	Valve %D	D.In	WLength 1-A
1	0.00	0.80	0	0	100	0	1	254
2	120.00	0.80	0	0	100	0	1	254

Operator: PH
Object name: BAS-SEL-wzor_(80:20_0.5Et3N_0.8ml_29C)_Det1-A - Created: 09-04-2002, 03:36
Path: >Testy>Chromatogramy>Sebastian>AS-H>Selvita

	Ret.time [min]	Start [min]	End [min]	Area [mAU*min]	Height [mAU]	% Area	Width [min]
1	17.771	15.43	19.61	259.919	187.467	14.3402	1.094
2	20.938	19.61	23.56	607.254	383.232	33.5032	1.384
3	24.922	23.56	28.44	94.3	41.5342	5.2027	1.855
4	30.390	28.44	37.50	851.053	365.877	46.9540	2.185

Operator: PH
Object name: BAS-SEL-wzor (80:20 0.5Et3N 0.8ml 29C)_Det1-A - Created: 09-04-2002, 02:49
Path: >Testy>Chromatogramy>Sebastian>AS-H>Selvita

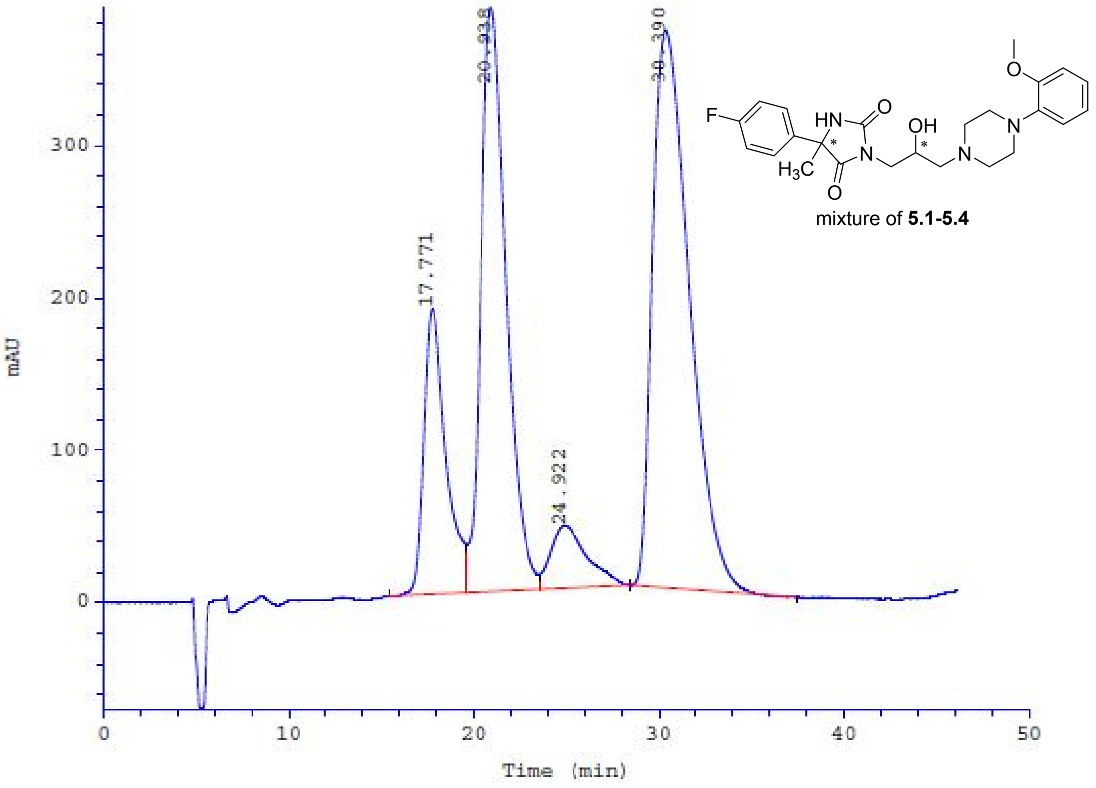

Page 1

Figure S36. The HPLC analysis for mixture of all isomers **5.1**; **5.2**; **5.3** and **5.4**

Object name: Time table - 100% C, 0.8 mL, 254 - Created: 19-02-2002, 02:13
Path:

	Time min	Flow ml/min	Valve %A	Valve %B	Valve %C	Valve %D	D.In	WLength 1-A
1	0.00	0.80	0	0	100	0	1	254
2	120.00	0.80	0	0	100	0	1	254

Operator: PH
Object name: BAS-SEL-13_(80:20_0.5Et3N_0.8ml_29C)_Det1-A - Created: 08-04-2002, 23:20
Path: >Testy>Chromatogramy>Sebastian>AS-H>Selvita

	Ret.time [min]	Start [min]	End [min]	Area [mAU*min]	Height [mAU]	% Area	Width [min]
1	18.736	16.30	19.67	32.0004	20.886	4.4233	1.176
2	20.820	19.67	23.74	673.608	435.67	93.1097	1.401
3	24.820	23.74	27.68	17.8478	8.84625	2.4670	1.535

Operator: PH
Object name: BAS-SEL-13 (80:20 0.5Et3N 0.8ml 29C)_Det1-A - Created: 08-04-2002, 22:18
Path: >Testy>Chromatogramy>Sebastian>AS-H>Selvita

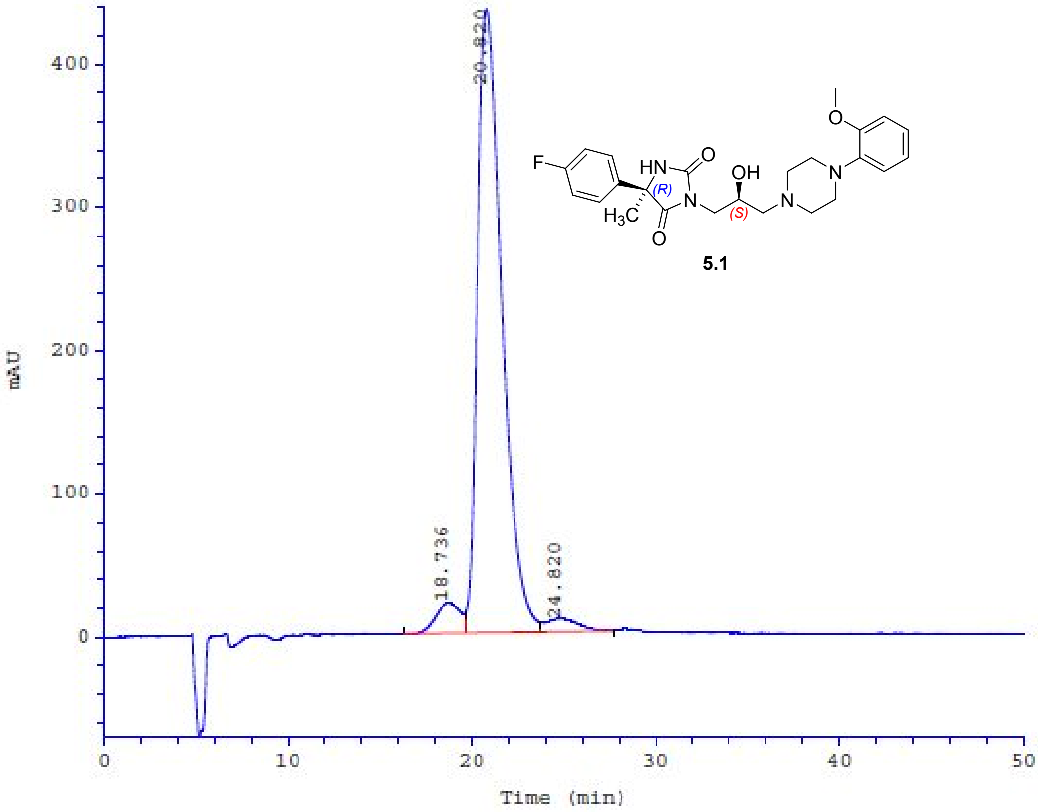

Page 1

Figure S37. The HPLC analysis for optically pure **5.1**

Object name: Time table - 100% C, 0.8 mL, 254 - Created: 19-02-2002, 02:13
Path:

	Time min	Flow ml/min	Valve %A	Valve %B	Valve %C	Valve %D	D.In	WLength 1-A
1	0.00	0.80	0	0	100	0	1	254
2	120.00	0.80	0	0	100	0	1	254

Operator: PH
Object name: BAS-SEL-11w_(80:20_0.5Et3N_0.8ml_29C)_Det1-A - Created: 09-04-2002, 01:48
Path: >Testy>Chromatogramy>Sebastian>AS-H>Selvita

	Ret.time [min]	Start [min]	End [min]	Area [mAU*min]	Height [mAU]	% Area	Width [min]
1	21.069	19.90	22.40	2.43099	2.04049	1.4482	1.100
2	24.786	22.64	29.25	165.432	79.3226	98.5518	1.915

Operator: PH
Object name: BAS-SEL-11w_(80:20_0.5Et3N_0.8ml_29C)_Det1-A - Created: 09-04-2002, 00:44
Path: >Testy>Chromatogramy>Sebastian>AS-H>Selvita

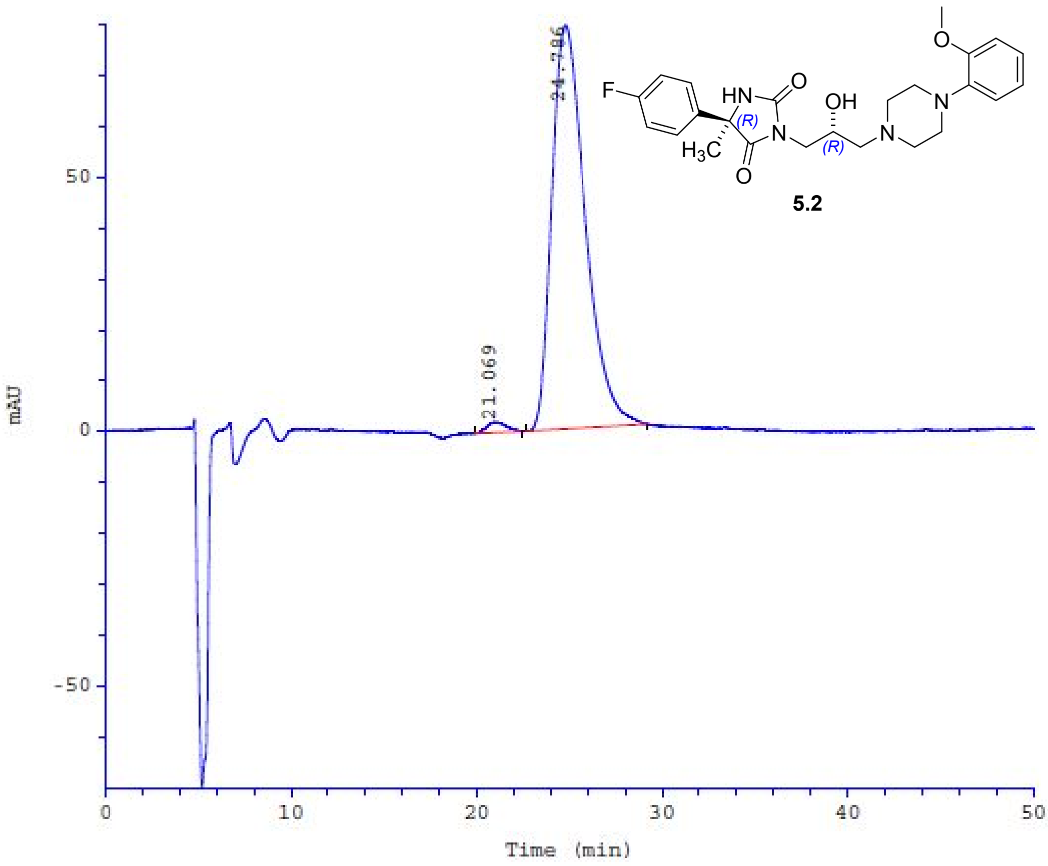

Page 1

Figure S38. The HPLC analysis for optically pure **5.2**

Object name: Time table - 100% C, 0.8 mL, 254 - Created: 19-02-2002, 02:13
Path:

	Time min	Flow ml/min	Valve %A	Valve %B	Valve %C	Valve %D	D.In	WLength 1-A
1	0.00	0.80	0	0	100	0	1	254
2	120.00	0.80	0	0	100	0	1	254

Operator: PH
Object name: BAS-SEL-12w_(80:20_0.5Et3N_0.8ml_29C)_Det1-A - Created: 09-04-2002, 04:18
Path: >Testy>Chromatogramy>Sebastian>AS-H>Selvita

	Ret.time [min]	Start [min]	End [min]	Area [mAU*min]	Height [mAU]	% Area	Width [min]
1	17.805	16.30	20.95	79.4557	67.0569	100.0000	1.073

Operator: PH
Object name: BAS-SEL-12w_(80:20_0.5Et3N_0.8ml_29C)_Det1-A - Created: 09-04-2002, 03:36
Path: >Testy>Chromatogramy>Sebastian>AS-H>Selvita

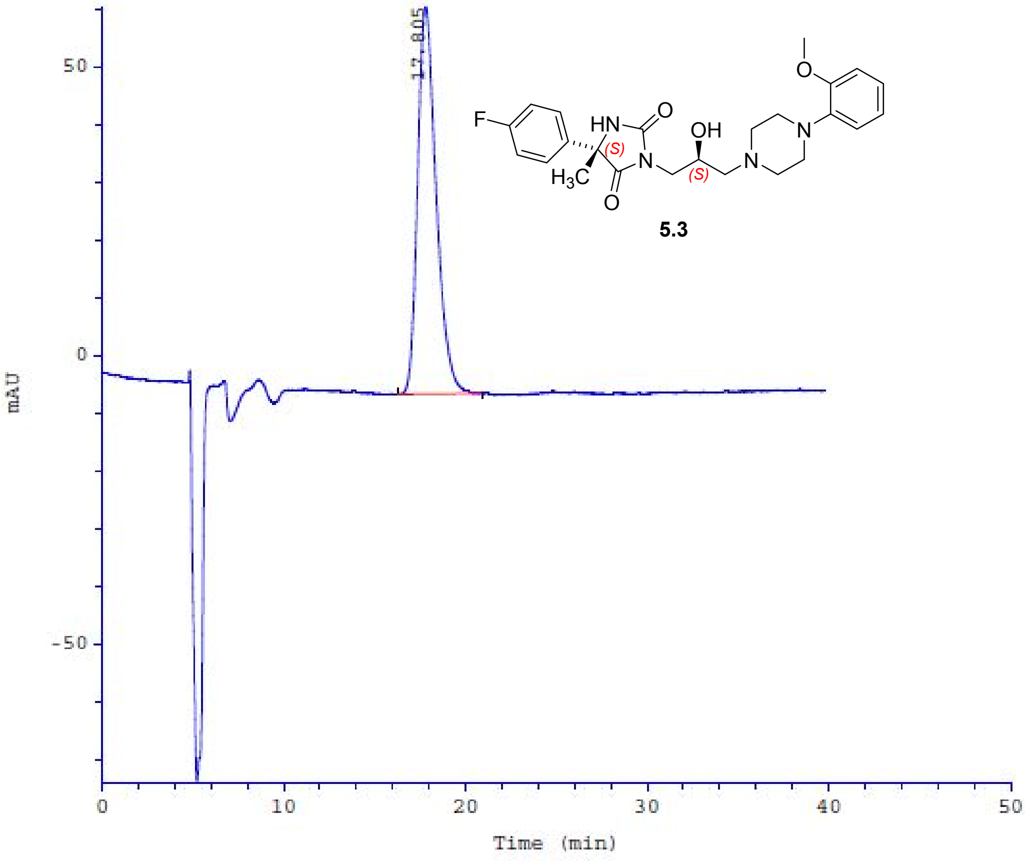

Page 1

Figure S39. The HPLC analysis for optically pure **5.3**

Object name: Time table - 100% C, 0.8 mL, 254 - Created: 19-02-2002, 02:13
Path:

	Time min	Flow ml/min	Valve %A	Valve %B	Valve %C	Valve %D	D.In	WLength 1-A
1	0.00	0.80	0	0	100	0	1	254
2	120.00	0.80	0	0	100	0	1	254

Operator: PH
Object name: BAS-SEL-14_(80:20_0.5Et3N_0.8ml_29C)_Det1-A - Created: 09-04-2002, 00:41
Path: >Testy>Chromatogramy>Sebastian>AS-H>Selvita

	Ret.time [min]	Start [min]	End [min]	Area [mAU*min]	Height [mAU]	% Area	Width [min]
1	17.818	16.42	18.86	10.8547	9.57152	4.6438	0.898
2	19.968	18.86	22.63	9.50035	4.46282	4.0644	1.680
3	25.052	23.04	27.45	4.24069	2.21971	1.8142	1.849
4	30.752	28.55	35.64	209.15	98.0957	89.4776	1.952

Operator: PH
Object name: BAS-SEL-14 (80:20 0.5Et3N 0.8ml 29C)_Det1-A - Created: 08-04-2002, 23:20
Path: >Testy>Chromatogramy>Sebastian>AS-H>Selvita

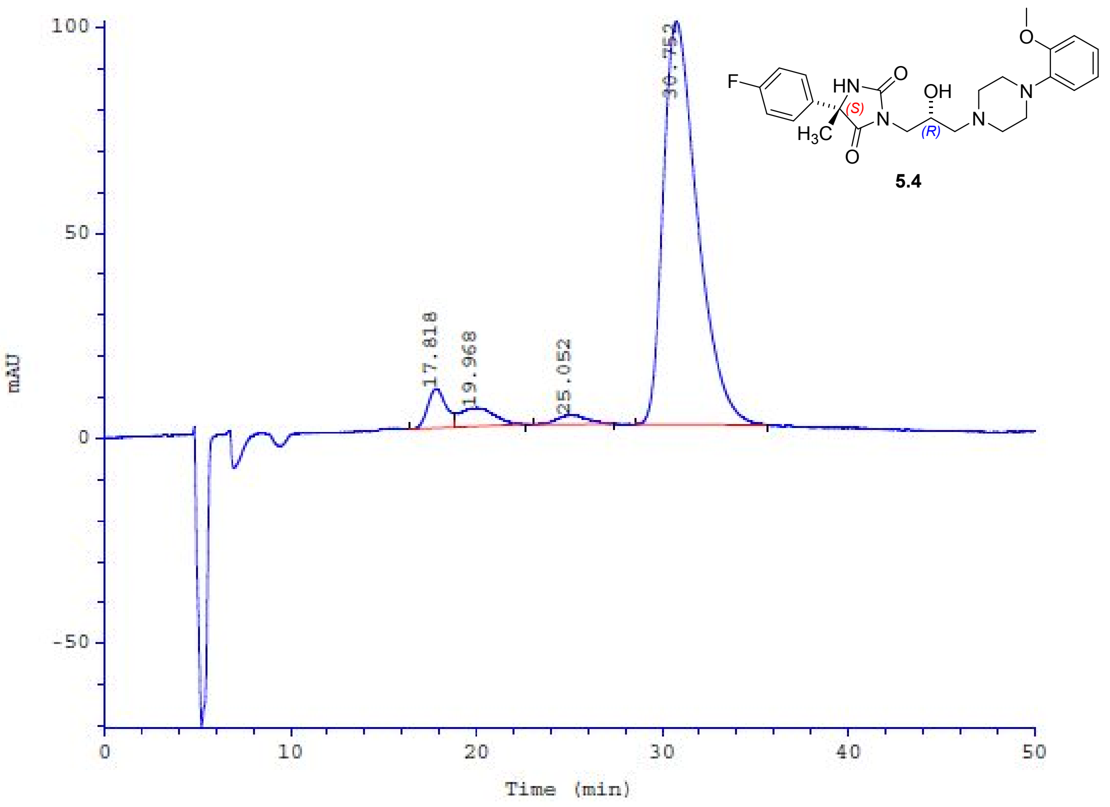

Page 1

Figure S40. The HPLC analysis for optically pure **5.4**

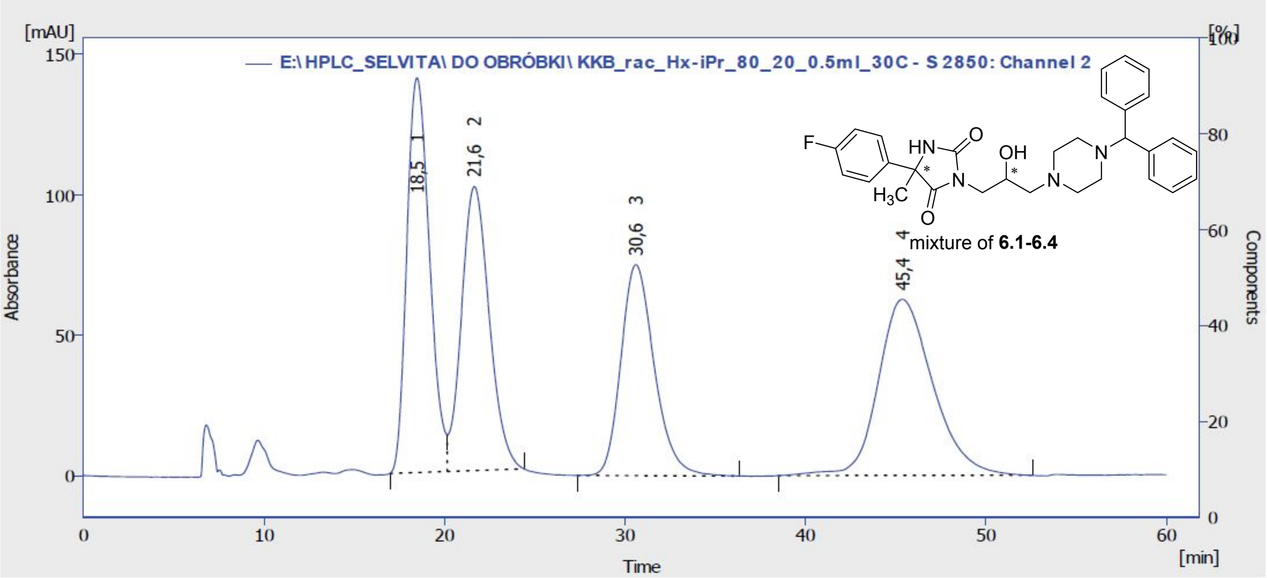

Result Table (Uncal - E:\HPLC_SELVITA\DO OBRÓBKI\KKB_rac_Hx-iPr_80_20_0.5ml_30C - S 2850: Chann

	Reten. Time [min]	Area [mAU.s]	Height [mAU]	Area [%]	Height [%]	W05 [min]	PDA Peak Purity	Compound Name
1	18,467	12261,937	140,520	26,7	37,0	1,38	998	
2	21,633	10844,025	101,110	23,6	26,6	1,67	999	
3	30,583	9670,982	75,113	21,0	19,8	1,95	815	
4	45,367	13205,847	62,691	28,7	16,5	3,17	854	
	Total	45982,791	379,434	100,0	100,0			

Figure S41. The HPLC analysis for mixture of all isomers **6.1**; **6.2**; **6.3** and **6.4**

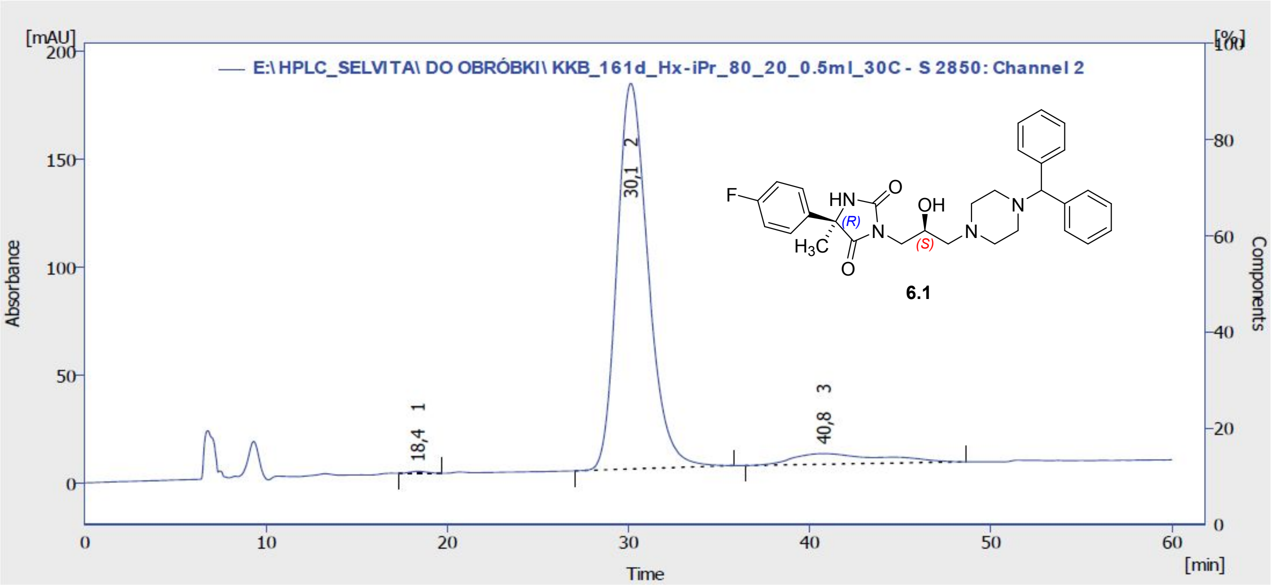

Result Table (Uncal - E:\HPLC_SELVITA\DO OBRÓBKI\KKB_161d_Hx-iPr_80_20_0.5ml_30C - S 2850: Chan

	Reten. Time [min]	Area [mAU.s]	Height [mAU]	Area [%]	Height [%]	W05 [min]	PDA Peak Purity	Compound Name
1	18,367	58,131	0,892	0,3	0,5	1,10	999	
2	30,117	21252,971	178,611	92,4	96,8	1,83	989	
3	40,767	1695,437	4,929	7,4	2,7	7,20	999	
	Total	23006,540	184,431	100,0	100,0			

Figure S42. The HPLC analysis for optically pure **6.1**

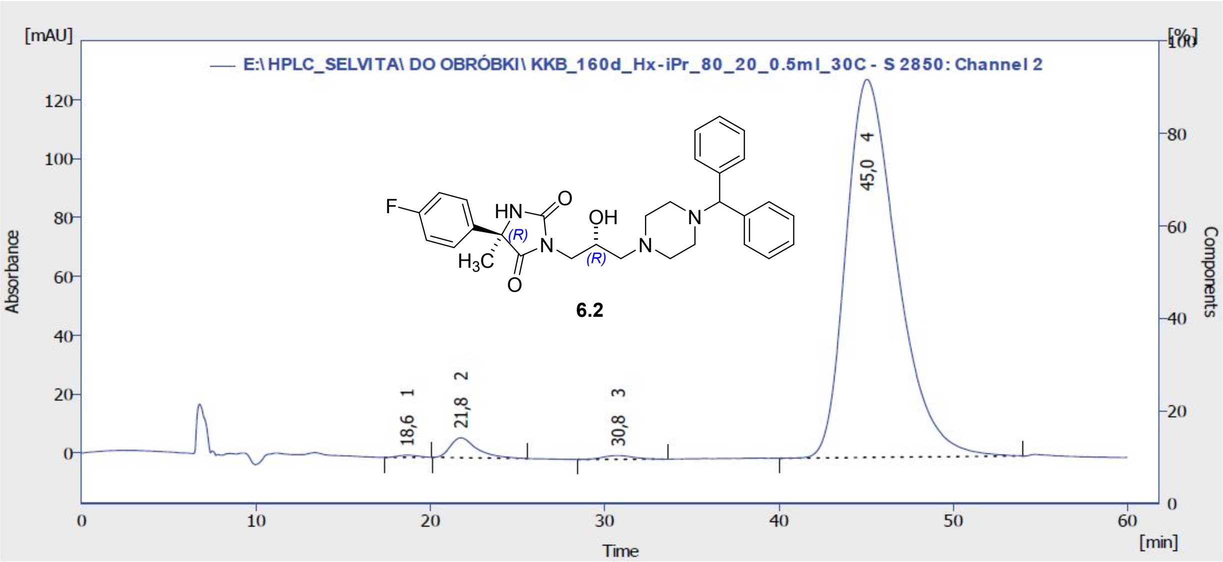

Result Table (Uncal - E:\HPLC_SELVITA\DO OBRÓBKI\KKB_160d_Hx-iPr_80_20_0.5ml_30C - S 2850: Chan

	Reten. Time [min]	Area [mAU.s]	Height [mAU]	Area [%]	Height [%]	W05 [min]	PDA Peak Purity	Compound Name
1	18,650	60,249	0,751	0,2	0,5	1,33	955	
2	21,750	717,074	6,754	2,7	4,9	1,53	884	
3	30,783	159,329	1,304	0,6	1,0	1,90	949	
4	45,050	26096,339	128,369	96,5	93,6	3,12	934	
	Total	27032,991	137,178	100,0	100,0			

Figure S43. The HPLC analysis for optically pure **6.2**

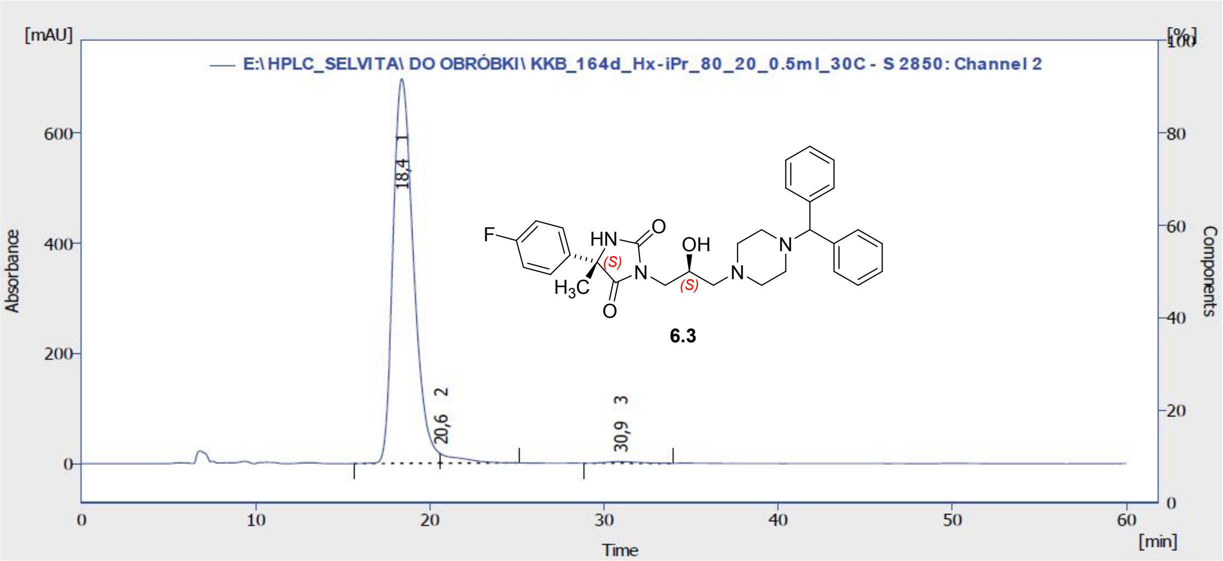

Result Table (Uncal - E:\HPLC_SELVITA\DO OBRÓBKI\KKB_164d_Hx-iPr_80_20_0.5ml_30C - S 2850: Chan

	Reten. Time [min]	Area [mAU.s]	Height [mAU]	Area [%]	Height [%]	W05 [min]	PDA Peak Purity	Compound Name
1	18,383	56947,397	696,738	97,0	97,2	1,28	958	
2	20,617	1383,636	16,984	2,4	2,4	1,30	996	
3	30,950	402,061	2,964	0,7	0,4	2,17	993	
	Total	58733,094	716,685	100,0	100,0			

Figure S43. The HPLC analysis for optically pure **6.3**

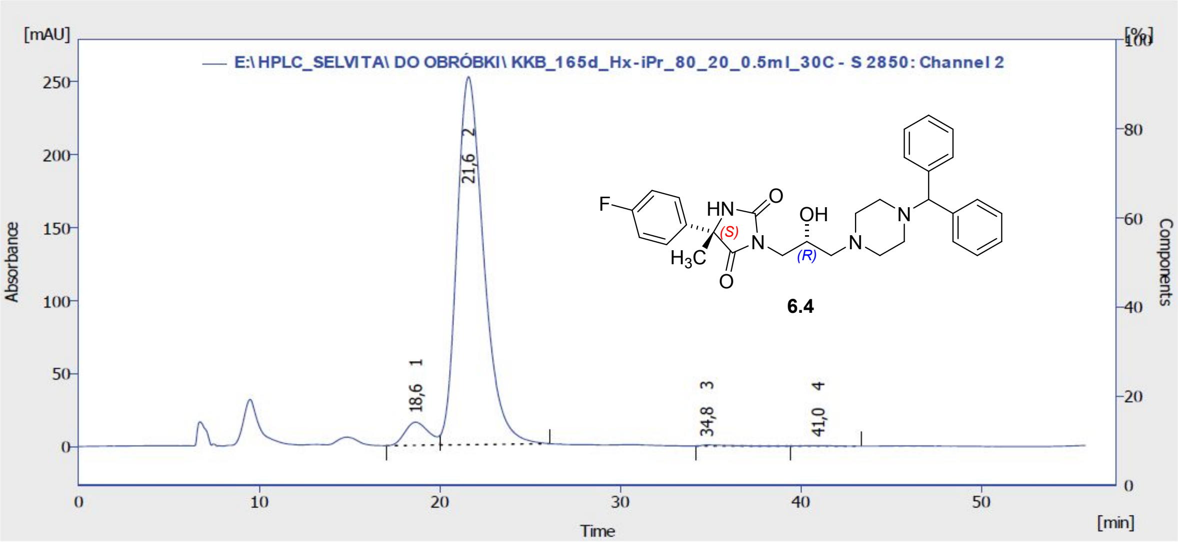

Result Table (Uncal - E: \HPLC_SELVITA \DO OBRÓBKI\KKB_165d_Hx-iPr_80_20_0.5ml_30C - S 2850: Chan

	Reten. Time [min]	Area [mAU.s]	Height [mAU]	Area [%]	Height [%]	W05 [min]	PDA Peak Purity	Compound Name
1	18,650	1467,861	15,902	5,3	5,9	1,52	994	
2	21,583	25985,672	251,905	94,2	93,7	1,58	993	
3	34,783	82,562	0,778	0,3	0,3	1,40	867	
4	40,967	38,272	0,275	0,1	0,1	2,40	913	
	Total	27574,366	268,860	100,0	100,0			

Figure S44. The HPLC analysis for optically pure **6.4**
